# Reduced Prevalence of Oral Human Papillomavirus (HPV) 4 Years after Bivalent HPV Vaccination in a Randomized Clinical Trial in Costa Rica

**DOI:** 10.1371/journal.pone.0068329

**Published:** 2013-07-17

**Authors:** Rolando Herrero, Wim Quint, Allan Hildesheim, Paula Gonzalez, Linda Struijk, Hormuzd A. Katki, Carolina Porras, Mark Schiffman, Ana Cecilia Rodriguez, Diane Solomon, Silvia Jimenez, John T. Schiller, Douglas R. Lowy, Leen-Jan van Doorn, Sholom Wacholder, Aimée R. Kreimer, for the CVT Vaccine Group

**Affiliations:** 1 Prevention and Implementation Group, International Agency for Research on Cancer, Lyon, France; 2 DDL Diagnostic Laboratory, Rijswijk, The Netherlands; 3 Division of Cancer Epidemiology and Genetics, National Cancer Institute, Bethesda, Maryland, United States of America; 4 Proyecto Epidemiológico Guanacaste, Fundación INCIENSA, Guanacaste, Costa Rica; 5 Division of Cancer Prevention, National Cancer Institute, Bethesda, Maryland, United States of America; 6 Laboratory of Cellular Oncology, National Cancer Institute, Bethesda, Maryland, United States of America; Karolinska Institutet, Sweden

## Abstract

**Background:**

Human papillomavirus (HPV) infection, particularly with type 16, causes a growing fraction of oropharyngeal cancers, whose incidence is increasing, mainly in developed countries. In a double-blind controlled trial conducted to investigate vaccine efficacy (VE) of the bivalent HPV 16/18 vaccine against cervical infections and lesions, we estimated VE against prevalent oral HPV infections 4 years after vaccination.

**Methods and Findings:**

A total of 7,466 women 18–25 years old were randomized (1∶1) to receive the HPV16/18 vaccine or hepatitis A vaccine as control. At the final blinded 4-year study visit, 5,840 participants provided oral specimens (91·9% of eligible women) to evaluate VE against oral infections. Our primary analysis evaluated prevalent oral HPV infection among all vaccinated women with oral and cervical HPV results. Corresponding VE against prevalent cervical HPV16/18 infection was calculated for comparison. Oral prevalence of identifiable mucosal HPV was relatively low (1·7%). Approximately four years after vaccination, there were 15 prevalent HPV16/18 infections in the control group and one in the vaccine group, for an estimated VE of 93·3% (95% CI = 63% to 100%). Corresponding efficacy against prevalent cervical HPV16/18 infection for the same cohort at the same visit was 72·0% (95% CI = 63% to 79%) (p versus oral VE = 0·04). There was no statistically significant protection against other oral HPV infections, though power was limited for these analyses.

**Conclusions:**

HPV prevalence four years after vaccination with the ASO4-adjuvanted HPV16/18 vaccine was much lower among women in the vaccine arm compared to the control arm, suggesting that the vaccine affords strong protection against oral HPV16/18 infection, with potentially important implications for prevention of increasingly common HPV-associated oropharyngeal cancer.

ClinicalTrials.gov, Registry number NCT00128661

## Introduction

A subset of oropharyngeal cancers (OPC) is caused by human papillomavirus (HPV) infection [1], with strong predominance of HPV16, which is detectable in about 90% of HPV-positive cases [2]. Evidence for the association between HPV and OPC has accumulated in recent years, and is based on extensive epidemiologic data and laboratory studies demonstrating molecular profiles indicative of high-risk HPV oncoprotein function [3].

HPV-positive OPC constitutes a distinct clinico-pathological entity with risk factors different from those for HPV-negative tumors. The incidence of OPC has increased significantly in the US [4], Australia [5], and several European countries [6]–[8], particularly in younger cohorts. In some areas, the increase in OPC has occurred despite declines in smoking and drinking, the main risk factors for HPV-negative OPC [4]. A recent study [9] showed that in the last 20 years, HPV detection in tumor specimens increased from 16% to 70% in the US. The authors estimated that in the next few decades, in the US, there will be more cases of HPV-positive OPC than of cervical cancer, where virtually all cases are attributable to HPV. In a report from Stockholm, Sweden [10], the incidence rate of HPV positive tonsillar cancers nearly doubled each decade between 1970 and 2007, while HPV negative tumors declined, leading the authors to suggest an epidemic of viral-induced carcinomas. The estimated number of new cases of OPC (including tonsils and base of tongue) is approximately 85 000 (ICD codes C01, C09-C10) per year in both sexes worldwide, with a male to female ratio of approximately 4∶1 [11].

Randomized trials have provided strong evidence for high efficacy of two virus-like particle (VLP) vaccines: the bivalent HPV16/18 vaccine (*Cervarix®,* GlaxoSmithKline Biologicals) [12], [13] and the quadrivalent HPV 6/11/16/18 vaccine (*Gardasil*™, Merck Sharp and Dohme) [14] against cervical [12], [14], vaginal and vulvar [14] infections and related diseases, and against anal HPV16/18 infections in women [15]. Among men, efficacy of the quadrivalent vaccine has been demonstrated against HPV-associated external genital lesions [16] and against anal HPV and intraepithelial neoplasia among men who have sex with men [17].

Oral anti-VLP antibodies are detectable in vaccinated subjects albeit at lower levels than those observed systemically [18], as is also true at the cervix [19]. Nonetheless, no studies have been reported on HPV vaccine efficacy (VE) in the oral cavity. Therefore, we evaluated efficacy of the bivalent vaccine to reduce oral HPV infection four-years following vaccination using data nested in our community-based double-blind randomized trial.

## Methods

### Ethics Statement

The protocol for this trial and supporting CONSORT checklist are available as supporting information; see Checklist S1 and Protocol S1.The trial was approved by institutional review boards of the National Cancer Institute in the US and the Instituto Costarricense de Investigación y Enseñanza en Nutrición y Salud (INCIENSA) in Costa Rica, and all participants signed IRB-approved consent forms. The trial is registered at clinicaltrials.gov, identifier NCT00128661.

### Study Procedures

This evaluation of VE against oral HPV infection was conducted in a randomized clinical trial initially designed to evaluate VE against persistent cervical HPV16/18 infection and precancerous lesions [13], [20], [21]. In 2004–2005, we invited a population sample of women aged 18–25 years from Guanacaste and Puntarenas, Costa Rica to participate. Women had to be good health, not pregnant or breastfeeding and using contraception during the vaccination period. 7 466 women were enrolled, representing 59·1% of eligible and 30·5% of women in the census [21].

At enrollment, a pelvic examination was performed on sexually experienced women, with collection of exfoliated cervical cells for liquid-based cytology and HPV DNA testing, and blood for HPV16/18 serology. Next, women were randomized in a blinded fashion to the bivalent vaccine or a hepatitis A vaccine (modified *Havrix®*, GSK Biologicals) as control. Both vaccines were formulated in three 0·5 ml doses and administered at enrollment, one and six months. Randomization was concealed for participants, clinical and laboratory staff and investigators throughout the study by using identical packaging and presentation of vaccines and coded labels, with vaccine allocation maintained by an independent Data Management Center. Additional details of this process have been previously reported [21]. Vaccines were assigned random identification numbers and each eligible participant was given the next available sequential number. Women not attending visits in allowable timeframes missed corresponding doses [21]. At annual follow-up visits, clinicians collected cervical cells for cytology and HPV testing from sexually active women, and those with abnormalities were referred for colposcopy and treatment as needed.

At the final blinded four-year study visit, after a new informed consent, a questionnaire was administered including oral and anal sexual behaviors and an oral specimen was collected by use of a 15-second rinse and 15-second gargle with 15 mL of commercially available alcohol-based mouth wash (Scope®, Procter and Gamble Company, Cincinnati, OH). This method of specimen collection [22] was chosen based on previous reports that a single mouthwash sample provides substantially larger amounts and higher molecular weight DNA than other methods of oral specimen collection [23], and that optimal specimen collection time is around 30 seconds after which point DNA recovery plateaus [24]. Specimens were kept between 2° and 8°Celsius until same-day processing at the local laboratory. The samples were concentrated by centrifugation (3000×g for 10 minutes) to obtain a pellet that was washed with 10 ml saline solution to remove residual mouthwash, re-centrifuged, and then resuspended in 1 ml of saline solution and frozen in liquid nitrogen tanks until testing.

For HPV DNA testing of oral and cervical specimens, DNA was extracted from each specimen via the MagNAPure LC DNA isolation procedure (Roche Diagnostics); 10 µl of extracted DNA were used for each PCR-reaction. All DNA samples were tested for the presence of HPV DNA by PCR amplification using the HPV SPF_10_ PCR-DEIA (DNA enzyme immunoassay)-LiPA_25_ (Line probe assay) version 1 system (Labo Biomedical Products, Rijswijk, The Netherlands). Briefly, this broad-spectrum PCR-based HPV DNA testing system uses SPF_10_ primers to amplify at least 57 HPV genotypes and the LiPA line detection system to genotype the following carcinogenic and non-carcinogenic HPVs [25], [26]: HPV 6, 11, 16, 18, 31, 33, 34, 35, 39, 40, 42, 43, 44, 45, 51, 52, 53, 54, 56, 58, 59, 66, 68/73, 70, and 74. To increase the sensitivity of type-specific detection of HPV16 and 18 using the SPF_10_ system, all specimens that were SPF_10_ PCR/DEIA-positive were tested for the presence of HPV16 or 18 using type-specific primers detected by the TS16 and TS18 DEIA system [27].

The first 300 samples collected were tested using multiple volumes (200 µl, 400 µl, 800 µl) for DNA extraction as part of our laboratory optimization phase. The remaining specimens were tested in three batches of approximately the same size using 400 µl for DNA extraction. While we did observe batch-associated differences in the proportion of individuals positive by DEIA who were negative by LiPA (in other words, HPV infections of unknown type), the fraction of specimens positive for HPV16/18 was constant across batches (between 0·2% and 0·4%, excluding pilot), as was the fraction of specimens positive for an oncogenic or non-oncogenic HPV type. Thus, the variation between the batches was only for HPVs of unknown types and not for HPV 16/18 or the other types analyzed. As part of quality control, the final batch was retested with HPV16 or 18 type-specific primers using the same technique as in the primary testing, adding two HPV16 and one HPV18 infections. We decided that the limited potential yield of re-testing the other batches did not justify the extensive testing effort and associated cost. In our primary analysis, all infections detected were included in the analysis. We also conducted a sensitivity analysis excluding specimens positive in the second test (see results).

Serum collected at enrollment was used to determine HPV16 and HPV18 serological status using a VLP-based direct ELISA, for detection of polyclonal antibodies (GlaxoSmithKline Biologicals, Rixensart, Belgium), as described previously [28].

### Statistical Analysis

Characteristics of women who accepted or declined the oral collection were compared using a chi-squared test for categorical variables. Among women who accepted, characteristics from the enrollment and four-year post-vaccination visits were compared by study arm. Median follow-up time was calculated and compared by arm using the Kruskal-Wallis test.

Before unblinding the data, we pre-specified our main objective for this analysis, which was an evaluation of VE against prevalent oral HPV16/18 infection approximately four years after the first vaccination among women with both oral and cervical HPV results available. Prevalence of oral HPV16/18 infections was the endpoint evaluated (defined as detection of either HPV16 or HPV18 or both in exfoliated cells from the oral cavity at the four-year study visit). VE against cervical HPV16/18 infections among the same women at the same time point are reported for comparison. Because this value-added component was introduced in response to the mounting evidence that HPV causes some oropharyngeal cancers, there was no pre-vaccination oral specimen obtained which would have allowed for exclusion from the analysis of women with prevalent oral HPV infection (as in a naïve cohort). To compensate, we pre-specified restricted cohorts in which to evaluate VE among women less likely to be exposed to HPV infection at vaccination, based on cervical HPV16/18 DNA or antibodies at enrollment. We also considered evaluating VE among women receiving fewer than three vaccine doses. However, given that only one subject in the vaccine arm had oral HPV16/18 detected 4 years after vaccination, these exploratory analyses became meaningless and were not formally conducted. We present in the results an estimate of VE among women who were HPV negative at the cervix.

Prevalences of oral and cervical HPV infections were expressed as number of infected women per 100 women vaccinated (stratified by vaccine arm). The complement of the ratios of the prevalence for the HPV and control arms constituted our VE estimates. We report asymptotic confidence intervals (95%CI) when cells had more than five events, and exact confidence limits otherwise [29], [30]. For analyses combining multiple HPV types, each woman was considered ‘positive’ if she harbored any of the types in question and ‘negative’ otherwise.

Oral and cervical VE estimates were compared using a GEE model [31] that accounts for correlation of oral/cervical infections within a woman. We also examined oral VE against other oncogenic HPV types and against HPV6/11, because of their association with laryngeal papillomatosis and the anatomical proximity of the larynx with the oral cavity.

At the time of this analysis, field work was on-going and individual information remained blinded. Thus, analyses were conducted by an external group, Information Management Systems (Rockville, MD), under the direction of the investigators. SAS 9.2 TS2M3 was used for analysis.

## Results

**Figure 1:**
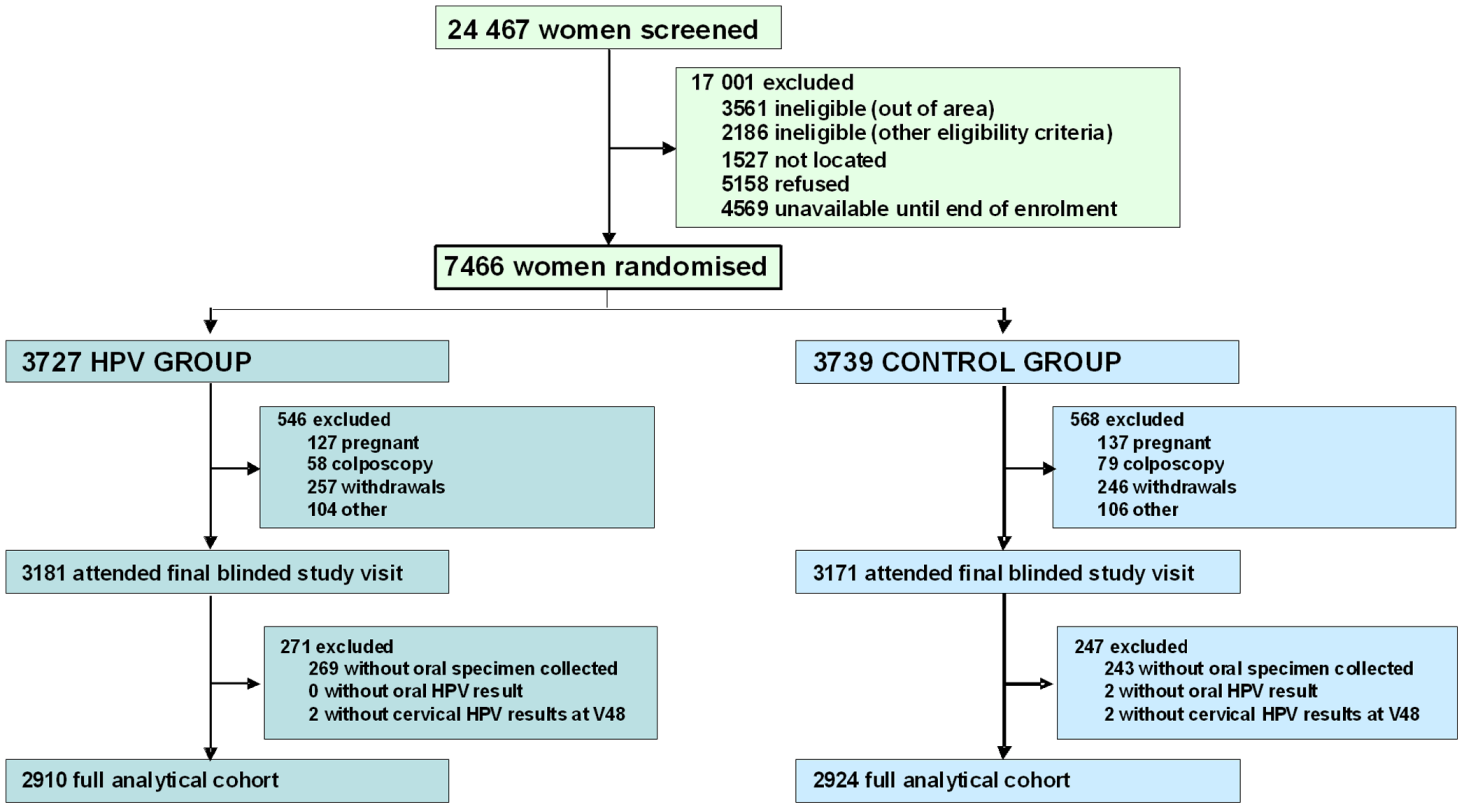
Consort diagram.

Of the 7 466 women randomized, 1 114 did not attend their four-year follow-up visit (Figure 1) and 6 352 attended the visit (3 181 HPV; 3 171 Control). 512 (269 HPV; 243 Control) women refused oral specimen collection, for an acceptance rate among eligible women of 91·9% (5 840 out of 6 352). After excluding two women with inadequate oral specimens and four women with unavailable cervical HPV results from the corresponding visit, the full analytic cohort comprised 5 834 women (2 910 HPV; 2 924 Control). The full cohort included all women vaccinated regardless of baseline cervical HPV DNA or serology results, treatment for cervical precancer or number of vaccine doses.

Percentages of women who accepted oral specimen collection were similar in both arms (91·5% vaccine and 92.3% control), although they were lower at one of the study clinics (Nicoya) (Table 1). Women with 4+ lifetime sexual partners, reporting oral and anal sex and positive for cervical HPV16/18 DNA at enrollment were significantly more likely to donate oral specimens.

**Table 1:** Proportion of women who accepted oral specimen collection among all women who attended the 4- year annual visit by selected characteristics.

Characteristic	Number of women*	Percent who accepted oral collection	p value∫		
**Age at Entry (in years)** ◊					0.59
18–19	1 859	91.6	.		
20–21	1 437	91.8	.		
22–23	1 340	92.8	.		
24–25	1 204	91.8	.		
**Study clinic**					<0.001
Liberia	1 661	92.6	.		
Nicoya	1 620	85.6	.		
Cañas	1 762	97.0	.		
Puntarenas	797	93.7	.		
**Lifetime number of vaginal sex partners at 4-year visit**					<0.001
0	324	84.4	.		
1	1 489	90.5	.		
2–3	2 007	92.4	.		
4+	2 020	94.0	.		
**Lifetime number of oral sex partners (reported at 4-year visit)**					<0.001
0	2 189	88.6	.		
1	2 105	93.3	.		
2+	1 516	95.3	.		
**Age at oral sex debut in tertiles (age)**					<0.001
Never Had Oral Sex	2 189	88.6	.		
1st tertile (11–19 years)	1 328	95.0	.		
2nd tertile (20–22 years )	1 216	93.3	.		
3rd tertile (23–32 years )	984	94.1	.		
**Lifetime number of anal sex partners (reported at 4-year visit)**					<0.001
0	4 768	90.9	.		
1	878	97.0	.		
2+	177	97.8	.		
**Cervical HPV16/18 DNA at Enrollment**					<0.001
Never had sex @ Enrollment	1 137	87.1	.		
Negative	4 184	92.8	.		
Positive	511	96.2	.		
**Vaccine Arm**				0.25	
Control (Havrix)	2 928	92.3	.		
HPV (Cervarix)	2 912	91.5	.		

* Unknown excluded from calculation.

∫ P value for the comparison of women who did and did not accept oral specimen collections.

◊ Two 17 yr olds are classified in the ‘18–19’ group and one 27 yr old is classified in the ‘24–25’ group.

Among women who agreed to oral specimen collection, there was balance in the HPV and control arms on enrollment characteristics: age at entry, number of clinic visits and self-reported vaginal, oral and anal sex, which were queried at the study visit corresponding to oral specimen collection, and on enrollment history of smoking and cervical HPV 16/18 DNA positivity (Table 2). Median follow-up time was 54·8 months for vaccine arm and 54·9 for control arm (p = 0·58).

**Table 2:** General characteristics of the analytic population by vaccination arm (N = 5834).

	HPV Arm		Control Arm	
Characteristic	Number of women*	Percent (Column)	Number of women*	Percent (Column)
**Age at Entry (in years)** ∫				
18–19	907	31.2	949	32.5
20–21	739	25.4	696	23.8
22–23	647	22.2	693	23.7
24–25	617	21.2	586	20.0
**Total number of clinic visits attended**				
1–2	11	0.4	13	0.4
3–5	1.846	63.4	1.795	61.4
6–8	784	26.9	813	27.8
9+	269	9.2	303	10.4
**Lifetime number of vaginal sex partners at 4-year visit**				
0	168	5.8	156	5.3
1	715	24.6	771	26.4
2–3	1.007	34.6	999	34.2
4+	1.020	35.1	998	34.1
**Lifetime number of oral sex partners (reported at 4-year visit)**				
0	1.070	37.0	1.117	38.4
1	1.081	37.3	1.021	35.1
2+	744	25.7	771	26.5
**Age at oral sex debut in tertiles (age)**				
Never Had Oral Sex	1.070	37.6	1.117	39.0
1st tertile (11–19)	660	23.2	667	23.3
2nd tertile (20–22)	610	21.4	604	21.1
3rd tertile (23–32)	505	17.8	478	16.7
**Lifetime number of anal sex partners (reported at 4-year visit)**				
0	2.356	81.2	2.407	82.5
1	463	16.0	414	14.2
2+	82	2.8	95	3.3
**Ever smoking (at enrolment)**				
No	405	13.9	400	13.7
Yes	2.504	86.1	2.519	86.3
**Study clinic** ◊				
Liberia	789	27.1	871	29.8
Nicoya	855	29.4	764	26.1
Cañas	880	30.2	878	30.0
Puntarenas	386	13.3	411	14.1
**Cervical HPV16/18 DNA at Enrollment**				
Never had sex @ Enrolment	551	19.0	584	20.0
Negative	2.117	72.8	2.063	70.7
Positive	239	8.2	272	9.3

* Unknown excluded from calculation.

∫ Two 17 yr olds are classified in the ‘18–19’ group and one 27 yr old is classified in the ‘24–25’ group.

◊ Chi square p value for difference by arm = 0.02.

Prevalence of detectable oral HPV at the four-year study visit in the control group was 5·4% including identifiable (typeable) and untypeable types, 1·7% for infection with typeable HPV types, 1·3% for infections with oncogenic HPV types and 0·8% for non-oncogenic types. HPV16 was the most common type detected among controls (0·4%). Additional analyses of the uncharacterized oral HPV types detected are on-going. Oral HPV prevalence in the control group was significantly higher among women who were HPV-DNA positive at the cervix (3.5%) compared to those who were negative (1.0%). There was also a statistically significant association with single marital status and increasing numbers of lifetime vaginal sex partners, but there was no clear association with self-reported oral or anal sex [32].

In the full cohort (Table 3), estimated VE against oral HPV16/18 infection approximately four-years after first vaccination was 93·3% (one infection in vaccine arm, 15 in control, 95%CI = 62·5% to 99·7%). Type-specific VE was 91·6% against HPV16 (one and twelve women in vaccine and control arm, respectively, 95% CI = 51·7% to 99·6%) and 100% against HPV18 (0 and 4 women in the vaccine and control arm, 95%CI = −12·0% to 100·0%). The corresponding VE against prevalent cervical HPV16/18 infection for the same cohort of women at the same visit was 72·0% (95%CI = 63·0% to 79·1%) (p *versus* oral HPV VE = 0·04). The VE estimate against cervical HPV16 was similar to that against HPV18.

**Table 3:** Estimated vaccine efficacy against oral and cervical HPV16 and 18 infections 4 years after vaccination.

Arm	Number of women	Number of women with infection*	Prevalence	95%CI	Vaccine efficacy	95%CI
**Oral Infections**						
**HPV16/18** ∫						
HPV	2910	1	0.0	0.0∶0.2		
Control	2924	15	0.5	0.3∶0.8	93.3%	62.5% to 99.7%
**HPV16**						
HPV	2910	1	0.0	0.0∶0.2		
Control	2924	12	0.4	0.2∶0.7	91.6%	51.7% to 99.6%
**HPV18**						
HPV	2910	0	0.0	0.0∶0.1		
Control	2924	4	0.1	0.0∶0.3	100%	−12.0% to 100%
**Cervical Infections**						
**HPV16/18** ∫						
HPV	2910	61	2.1	1.6∶2.7		
Control	2924	219	7.5	6.6∶8.5	72.0%	63.0% to 79.1%
**HPV16**						
HPV	2910	44	1.5	1.1∶2.0		
Control	2924	151	5.2	4.4∶6.0	70.7%	59.3% to 79.3%
**HPV18**						
HPV	2910	18	0.6	0.4∶1.0		
Control	2924	78	2.7	2.1∶3.3	76.8%	61.9% to 86.5%

* There was one woman with a mixed infection with HPV 16 and 18.

∫ P for arm* site interaction for VE against HPV 16/18 = 0.04.

The subject in the vaccine arm who had an oral HPV infection received only two vaccine doses, as did another 328 in the vaccine arm and 294 in the control arm. In addition, the HPV infection in this particular subject was only detected when her oral specimen was retested as part of quality control. Therefore, in the sensitivity analysis excluding HPV positive results from retested specimens, the VE against oral HPV16/18 infections was 100·0% (zero infection in the vaccine arm, thirteen in the control arm, 95% CI = 74·0% to 100·0%). When excluding women who were HPV 16/18 positive in the cervix at the enrolment visit, VE was 91.7% (95%CI = 52.3% to 99.6%).

There was no evidence of statistically significant protection against HPV31, 51, 52, 56, 39, or 6/11(Table 4). Estimated VE against HPV 31 (N = 8 total oral infections across both arms), the type for which cross-protection has been reported most consistently, was 39·7% (95% CI = −161·0 to 88·1%). Estimated VE against oncogenic types excluding HPV 16 and 18 was 13·2% (95% CI = −61·1, 53·6).and the VE against all oncogenic HPV types combined was 45·7% (95% CI = 6·9% to 69·0%).

**Table 4:** Estimated vaccine efficacy against oral infections with other HPV types.

Arm	Number of women	Number of women with infection	Prevalence	95%CI	Vaccine efficacy	95%CI
**HPV31**						
HPV	2910	3	0.1	0.0∶0.3		
Control	2924	5	0.2	0.1∶0.4	39.7%	−161.0% to 88.1%
**HPV51**						
HPV	2910	7	0.2	0.1∶0.5		
Control	2924	10	0.3	0.2∶0.6	29.7%	−86.9% to 74.7%
**HPV52**						
HPV	2910	3	0.1	0.0∶0.3		
Control	2924	7	0.2	0.1∶0.5	56.9%	−63.9% to 91.0%
**HPV56**						
HPV	2910	2	0.1	0.0∶0.2		
Control	2924	4	0.1	0.0∶0.3	49.8%	−183.2% to 93.6%
**HPV39**						
HPV	2910	3	0.1	0.0∶0.3		
Control	2924	1	0.0	0.0∶0.2	−201.4%	−7836.8% to 67.9%
**HPV6/11**						
HPV	2910	4	0.1	0.0∶0.3		
Control	2924	4	0.1	0.0∶0.3	−0.5%	−345.5% to 77.3%
**Other oncogenic**						
HPV	2910	19	0.7	0.4∶1.0		
Control	2924	22	0.8	0.5∶1.1	13.2%	−61.1% to 53.6%
**All oncogenic**						
HPV	2910	20	0.7	0.4∶1.0		
Control	2924	37	1.3	0.9∶1.7	45.7%	6.9% to 69.0%

## Discussion

In this first report evaluating efficacy of an HPV vaccine against oral infection, we observed, as part of a randomized trial of the bivalent vaccine among young women in Costa Rica, a 93·3% reduction of prevalent oral HPV 16/18 infection in the vaccine arm compared to the control arm approximately four years after vaccination.

Because our randomized trial was not specifically designed to evaluate VE against oral HPV infections, we had no baseline information on oral HPV status from study subjects, and we had to rely on HPV prevalence four years after vaccination rather than incidence of new infections. However, the VE estimate from a study restricted to HPV negative women at baseline would likely have been higher than the observed VE of 93%. Vaccination is known to be ineffective against established infections [20], and therefore inclusion of women already infected at baseline would tend to attenuate the VE estimates. For example, VE against prevalent cervical HPV16/18 infection at the same visit increased from 72.0% (95%CI = 63.0% to 79.1%, table 3) to 80.4% (95%CI = 72.4% to 86.4%) when excluding infections present at enrollment. Thus, although we recognize that the lack of insight into incident oral HPV infections is an important limitation of this analysis, we consider that the strong reduction in oral HPV 16/18 prevalence 4 years after vaccination is unlikely to be explained by this aspect of the study design.

There is limited knowledge about natural history of oral HPV infection, and the quantitative relationship between one-time detection of HPV in oral exfoliated cells and risk of future OPC is not established. In this context, our study does not constitute direct evidence that the vaccine prevents OPC. However, the high VE against oral HPV16/18 infection supports the possibility that vaccination may reduce risk of HPV-positive OPC, in particular HPV 16, the type most commonly associated with this cancer.

Although surrogate clinical endpoints, such as CIN2 or worse, were used to establish VE against cervical cancer, leading to licensing and mass vaccination programs, that approach is not possible with OPC because it lacks established precursor lesions. Direct evaluation of VE against OPC seems impractical, because given the relative rarity of both infection and OPC and the probably long interval between infection and the occurrence of cancer, such evaluation would require large studies and probably decades to complete. However, additional studies using virologic outcomes may further define the potential utility of HPV vaccines in prevention of these cancers in men and women.

We believe this study constitutes a valid randomized evaluation of VE against prevalent oral infections because the study and laboratory testing were blinded and there was balance by arm on demographic characteristics and risk factors for oral HPV acquisition. More than 90% of women agreed to donate oral specimens and valid HPV results were obtained on all but two of them. Although women who donated an oral specimen had evidence of more sexual activity than the relatively small number of women who did not, the balance by arm on all relevant characteristics is reassuring. In addition, the low prevalence of oral HPV16 infection in our control group (0·4%) was similar to its reported prevalence among healthy subjects in low-risk populations reported in a pooled analysis of 18 studies [33] and comparable to a prevalence of 0·3% reported among women 14–69 years old in a recent large survey in the US [34]. As is typically noted, the prevalence of cervical HPV16 detection in our control group was an order of magnitude higher (5·2%).

VE against prevalent oral HPV16/18 infections was significantly higher than against corresponding cervical HPV16/18 infections (p = 0·04) in the same cohort without excluding enrollment prevalent infections. This is consistent with the possibility that most oral infections were incident, with very few prevalent at enrollment and persisting for four years. Further, oral sex tended to start later than vaginal sex (data not shown), which would also result in a larger fraction of oral infections being acquired after vaccination compared to cervical infections. However, we did not see a clear association of oral HPV infection with oral sex.

Until now, there have been no data on efficacy of any of the HPV vaccines for prevention of oral HPV infection, and this remains the case in men. However, it is likely that the protection we observed among women will also be present in men, as VE of both vaccines has been demonstrated against HPV infections among men and women at all mucosal sites evaluated. Our results suggest that administration of the HPV vaccine will guard against oral infection by the HPV types responsible for the vast majority of HPV-related OPC, and open the possibility of primary prevention of these increasingly common malignancies.

## Supporting Information

Checklist S1 **CONSORT checklist.**
(DOC)

Protocol S1 **Trial protocol.**
(PDF)
