# Supplementary material for: Reduced Prevalence of Oral Human Papillomavirus (HPV) 4 Years after Bivalent HPV Vaccination in a Randomized Clinical Trial in Costa Rica
Source: PLoS One. 2013 Jul 17;8(7):e68329. doi: 10.1371/journal.pone.0068329 (PMC3714284; doi:10.1371/journal.pone.0068329)
Supplement: Protocol S1 — Trial protocol. (PDF) [file pone.0068329.s002.pdf]

Title: **A double blind, controlled, randomized, phase III study of the efficacy of an HPV16/18 VLP vaccine in the prevention of advanced cervical intraepithelial neoplasia (CIN2, CIN3, adenocarcinoma *in situ* [AIS] and invasive cervical cancer) associated with HPV16 or HPV18 cervical infection in healthy young adult women in Costa Rica**

**Study Vaccines:**	Candidate vaccine: HPV16/18 virus-like particles with AS04 adjuvant
**Control vaccine:**	Investigational formulation of Havrix® containing 720 ELISA units of Hepatitis A antigen
**NCI Protocol number:**	04-C-N191
**GSK Protocol number:**	580299/009 (HPV-009)
**Investigational New Drug (IND) number:**	BB-IND 7920 (held by GSK Biologicals)
**Version/Date:**	Final version: 12 May 2004<br>Amendment 1: 29 March 2005<br>Amendment 2: 31 October 2005<br>Amendment 3: 19 May 2006<br>Amendment 4: 13 November 2008<br>***Amendment 5: 10 September 2009***
**Coordinating authors:**	Allan Hildesheim, NCI Principal Investigator and Rolando Herrero, Costa Rica Principal Investigator
**Medical Monitor:**	Mark Schiffman, NCI
**Contributing authors:**	Sholom Wacholder, NCI Statistician<br>Douglas Lowy, NCI Virologist<br>John Schiller, NCI Virologist

Sponsor Signatory Approval

**NCI Protocol number:**	04-C-N191
**GSK Protocol number:**	580299/009 (HPV-009)
**IND number:**	BB-IND 7920 (held by GSK Biologicals)
**Date of approval:**	Final Version: 12 May 2004
	Amendment 1: 29 March 2005<br>Amendment 2: 31 October 2005<br>Amendment 3: 19 May 2006<br>Amendment 4: 13 November 2008<br>***Amendment 5: 10 September 2009***
**Title:**	A double blind, controlled, randomized, phase III study of the efficacy of an HPV16/18 VLP vaccine in the prevention of advanced cervical intraepithelial neoplasia (CIN2, CIN3, adenocarcinoma *in situ* [AIS] and invasive cervical cancer) associated with HPV16 or HPV18 cervical infection in healthy young adult women in Costa Rica
**NCI Sponsor signatory:**	Allan Hildesheim, Ph.D.<br>Environmental Epidemiology Branch<br>Division of Cancer Epidemiology and Genetics (DCEG)<br>National Cancer Institute<br>6120 Executive Boulevard, Room 7062<br>Rockville, MD 20852-7234<br>Tel: 301-496-1691<br>Fax: 301-402-0916<br>Email: hildesha@exchange.nih.gov
**Signature:**	______________________________
**Date:**	______________________________
**GSK Sponsor signatory:**<br>**(Amended: 13 Nov 2008)**	Dominique Descamps<br>Director, Clinical Development<br>HPV vaccines, GlaxoSmithKline Biologicals
**Signature:**	______________________________
**Date:**	______________________________

For internal use only
{cars_md5_line_0 }
{cars_md5_line_1 }
{cars_md5_line_2 }
{cars_md5_line_3 }

Investigator Agreement

**NCI Protocol number:**	04-C-N191
**GSK Protocol number:**	580299/009 (HPV-009)
**IND number:**	BB-IND 7920 (held by GSK Biologicals)
**Title:**	A double blind, controlled, randomized, phase III study of the efficacy of an HPV16/18 VLP vaccine in the prevention of advanced cervical intraepithelial neoplasia (CIN2, CIN3, adenocarcinoma in situ [AIS] and invasive cervical cancer) associated with HPV16 or HPV18 cervical infection in healthy young adult women in Costa Rica

I agree:

- To assume responsibility for the proper conduct of the study at this site.
- To conduct the study in compliance with this protocol and with any mutually agreed future protocol amendments.
- Not to implement any changes to the protocol without agreement from the sponsors (NCI and GSK Biologicals) (Amended: 29 Mar 2005) and prior review and written approval from the Institutional Review Board (IRB) or Independent Ethics Committee (IEC), except where necessary to eliminate an immediate hazard to the subjects, or for administrative aspects of the study (where permitted by all applicable regulatory requirements).
- That I am thoroughly familiar with the appropriate use of the vaccine(s), as described in this protocol, and any other information provided by the sponsor, including, but not limited to, the following: the current Investigator's Brochure (IB) or equivalent document, IB supplement (if applicable), prescribing information (in the case of a marketed vaccine) and/or Master Data Sheet (if the Master Data Sheet exists and serves as reference document for the vaccine in the case of a marketed vaccine).
- That I am aware of, and will comply with, "Good Clinical Practices" (GCP) and all applicable regulatory requirements.
- That I have been informed that certain regulatory authorities require the sponsor (GSK Biologicals) (Amended: 29 Mar 2005) to obtain and supply, as necessary, details about the investigator's ownership interest in the sponsor or the investigational product, and more generally about his/her financial ties with the sponsor (GSK Biologicals) (Amended: 29 Mar 2005). GSK Biologicals will use and disclose the information solely for the purpose of complying with regulatory requirements.

Hence I:

- Agree to supply GSK Biologicals with any necessary information regarding ownership interest and financial ties (including those of my spouse and dependent children).
- Agree to promptly update this information if any relevant changes occur during the course of the study and for 1 year following completion of the study.
- Agree that GSK Biologicals may disclose any information it has about such ownership interests and financial ties to regulatory authorities.
- Agree to provide GSK Biologicals with an updated Curriculum Vitae and other FDA required documents.

Investigator name: Rolando Herrero, MD

_______________________________ _______________
Investigator signature Date

For internal use only
{cars_md5_line_0 }
{cars_md5_line_1 }
{cars_md5_line_2 }
{cars_md5_line_3 }

List of Abbreviations

AE	Adverse event
AER	Adverse Event Report form
AGC	Atypical glandular cells (endocervical, endometrial, or glandular; favor neoplastic or not otherwise specified)
AIS	Adenocarcinoma *in situ*
$Al(OH)_3$	Aluminum hydroxide
AS04	Investigational adjuvant consisting of aluminum salts and MPL®. Formerly called SBAS4
ASC	Atypical squamous cells (includes ASC-H and ASC-US)
ASC-H	Atypical squamous cells – cannot exclude high-grade squamous intraepithelial lesion
ASC-H+	Atypical squamous cells – cannot exclude high-grade squamous intraepithelial lesion that are positive for HPV by HCII Probe B
ASC-US	Atypical squamous cells of undetermined significance
ASC-US+	Atypical squamous cells of undetermined significance that are positive for HPV by HCII Probe B
ATP	According to protocol
BBI	Boston Biomedica Inc.
BB-IND	Biologics Bureau-Investigational New Drug Application
BLA	Biologic License Application
°C	Degrees Centigrade
CCR	Center for Cancer Research
CFR	Code of federal regulations
CIF	Colposcopy Intake Form
CIN	Cervical intraepithelial neoplasia

CIN2+	Cervical intraepithelial neoplasia grades 2/3, adenocarcinoma *in situ*, and invasive cervical cancer
CIS	Carcinoma *in situ*
CONIS	Scientific Research National Council of the Ministry of Health, Costa Rica
CRA	Clinical Research Associate
CRF	Case Report Form
CRVT	Costa Rican Vaccine Trial data management system
CTA	Clinical Trials Agreement
DCEG	Division of Cancer Epidemiology and Genetics
DHHS	Department of Health and Human Services
DMC	Data management center
DNA	Deoxyribonucleic acid
DSMB	Data and Safety Monitoring Board
E6/E7	Non-structural HPV proteins
EIA	Enzyme Immunoassay
EISR	Expedited Investigator Safety Report
ELISA	Enzyme-linked immunoabsorbent assay
EL.U	ELISA Units
ER	Emergency Room
ES	Initial Visit Eligibility Screener form
EU/mL	ELISA Units per millilitre
FDA	Food and Drug Administration
FUNIN	Foundation for the Costa Rican Institute for Research and Training in Nutrition and Health
FWA	Federal-wide assurance
GCP	Good clinical practices

GMP	Good manufacturing practices
GMT	Geometric mean titer
GSK	GlaxoSmithKline
HAV	Investigational formulation of Hepatitis A virus Havrix® vaccine
HBsAg	Hepatitis B surface antigen
HCII	Hybrid Capture® 2 test (Digene Corporation, Gaithersburg, MD, USA)
HLA	Human leukocyte antigen
HPV	Human papillomavirus
HSIL	High-grade squamous intraepithelial lesion
HV2	Home Visit Reactogenicity Monitoring form
IB	Investigator Brochure
ICD-10	International Classification of Diseases – 10
ICF	Informed Consent Form
ICH	International Conference on Harmonization
ID	Identification
IEC	Independent Ethics Committee
IMS	Information Management Services, Inc.
INCIENSA	Costa Rican Institute for Research on Nutrition and Health
IND	Investigational New Drug Application
INDSR	Investigation New Drug Safety Report (Amended: 29 Mar 2005)
IRB	Institutional Review Board
ITT	Intent to treat
kg	Kilogram
L1 proteins	Papillomavirus structural proteins
LBA	Lineblot assay

LEEP	Loop Electrosurgical Excisional Procedure
LiPA	Line probe assay
LSIL	Low-grade squamous intraepithelial lesion
MA	Massachusetts, USA
MATLAB®	Matrix Laboratory (Mathworks, Inc.)
μg	Microgram
MD	Maryland, USA or Doctor of Medicine
mg	Milligram
mL	Milliliter
mm	Millimeter
MPL®	Monophosphoryl lipid A (Corixa Corporation, Seattle, Washington, USA [Formerly Ribi])
MSDS	Material Safety Data Sheet (Amended: 29 Mar 2005)
NCI	National Cancer Institute
NCSS	Number Cruncher Statistical System
NIH	National Institutes of Health
PASS	Power analysis and sample size program
PBMC	Peripheral blood mononuclear cells
PCR	Polymerase chain reaction
PES	Post-Enrollment Eligibility Screener form
PGMY	PCR-based primers
PI	Principal Investigator
PLA	Product License Application
POD	Peroxidase
QA	Quality assurance

QC	Quality control
RBC	Red blood cells
RI	Rhode Island, USA
RNA	Ribonucleic acid
SAE	Serious adverse event
SAS	Statistical Analysis System
SIL	Squamous intraepithelial lesion
SOP	Standard operating procedure
SPF	PCR-based primers
TMB	Tetra methyl benzidine
US or USA	United States of America
UT	Utah, USA
VAR	Vaccine Administration and Reactogenicity Monitoring form
VLP	Virus-like particle

Glossary of Terms

Adverse event	Any untoward medical occurrence in a patient or clinical investigation subject, temporally associated with the use of a medicinal product, whether or not considered related to the medicinal product.<br><br>An AE can therefore be any unfavorable and unintended sign (including an abnormal laboratory finding), symptom, or disease (new or exacerbated) temporally associated with the use of a medicinal product.
Blinding	A procedure in which one or more parties to the trial are kept unaware of the treatment assignment in order to reduce the risk of biased study outcomes. When the subjects, the investigator, and the sponsor staff who are involved in the treatment or clinical evaluation of the subjects and review/analysis of data are unaware of the treatment assignments, as is the case for the present trial, the study is double blind.
Eligible	Qualified for enrolment into the study based upon strict adherence to inclusion/exclusion criteria.
Evaluable	Meeting all eligibility criteria, complying with the procedures defined in the protocol, and, therefore, included in the according-to-protocol (ATP) analysis.
Investigational product	A pharmaceutical form of an active ingredient or placebo being tested or used as a reference in a clinical trial, including a product with a marketing authorization when used in a way different from the approved form, or when used for an unapproved indication, or when used to gain further information about an approved use.
Medical Monitor	An individual medically qualified to assume the responsibilities of the sponsor (GSK Biologicals) especially in regards to the ethics, clinical safety of a study and the assessment of adverse events.
Protocol amendment	ICH defines a protocol amendment as “A written description of a change(s) to or formal clarification of a protocol.” This is further detailed to include a change to an approved protocol that affects the safety of subjects, scope of the investigation, study design, or scientific integrity of the study.

Protocol administrative change	A protocol administrative change addresses changes to only logistical or administrative aspects of the study. N.B. Any change that falls under the definition of a protocol amendment (see definition above) must be prepared as an amendment to the protocol.
Randomization	Process of random attribution of treatment to subjects in order to reduce bias of selection.
Solicited adverse event	Adverse events (AEs) to be recorded as endpoints in the clinical study. The presence/occurrence/intensity of these events is actively solicited from the subject or an observer during a specified post-vaccination follow-up period.
Subject	Term used throughout the protocol to denote an individual that has been contacted in order to participate in the clinical study, either as a recipient of the investigational product(s) or as a control.
Unsolicited adverse event	Any adverse event (AE) reported in addition to those solicited during the clinical study. Also any “solicited” symptom with onset outside the specified period of follow-up for solicited symptoms will be reported as an unsolicited adverse event.

TABLE OF CONTENTS

PAGE

SYNOPSIS....16

1. INTRODUCTION....22
  1.1. Background....22
  1.2. The Need for an HPV Vaccine....25
  1.3. Defining Cervical Abnormalities....25
  1.4. Summary....26

2. STUDY OBJECTIVES....27
  2.1. Primary Objective....27
  2.2. Secondary Objectives....27
  2.3. Tertiary Objectives....28
  2.4. Ancillary Analyses & Studies....29

3. METHODS....29
  3.1. Overview of the Trial....29
  3.2. Study Population....37
  3.3. Eligibility Criteria....37
    3.3.1. Inclusion Criteria....37
    3.3.2. Exclusion Criteria....38
    3.3.3. Deferral Criteria....40
    3.3.4. Elimination criteria from the ATP analyses during the study....41
  3.4. Contraindications to Subsequent Vaccination....41
  3.5. Trial Arms & Randomization....42
  3.6. Study Vaccines....43
  3.7. Selection of an Investigational Formulation of *Havrix* as the Control Vaccine....44
  3.8. Central Storage of Vaccine....45
  3.9. Concomitant Medications/Vaccination....46
  3.10. Vaccination Route and Schedule....46
  3.11. Clinic Visits During Vaccination Phase (0, 1, 6, & 7 months)....46
  3.12. Active Follow-up of Participants (Months 12-48)....48
  3.13. Trial Endpoints....49
    3.13.1. Primary Endpoint....49
    3.13.2. Secondary Endpoints....49
    3.13.3. Tertiary and Exploratory Endpoints (Amended: 13 Nov 2008)....51
  3.14. Adverse Events Monitoring....51
  3.15. Clinical Procedures....54
    3.15.1. Overview....54
  3.16. Enrollment Physical Examination....54
    3.16.1. Pelvic Examinations....54
    3.16.2. Referral for Colposcopic Evaluation....56
    3.16.3. Exiting Women from the Trial (Amended: 13 Nov 2008)....56
  3.17. Other Procedures and Data Collection....57
    3.17.1. Overview....57

3.17.2. Interview Questionnaires 58
3.17.3. Blood Collection 58
3.17.4. Extra-Cervical Collection 59
3.18. Biospecimen Processing, Storage, & Shipment 59
3.19. Laboratory Tests 61
3.19.1. Cytology 61
3.19.2. Histology 62
3.19.3. PCR typing of HPV DNA from *PreservCyt* specimens 63
3.19.4. PCR typing of HPV DNA from histopathological specimens 63
3.19.5. HPV DNA Testing by *Hybrid Capture 2* (HCII) 64
3.19.6. Chlamydia trachomatis & Neisseria gonorrhoeae Testing (Hybrid Capture 2, HCII) 65
3.19.7. Serology Testing by ELISA 65
3.19.8. V5/J4 Monoclonal Inhibition Enzyme Immunoassay (EIA) Antibodies on a Subset of Subjects 66
3.19.9. Other Assays 66
3.20. Efforts to Maximize Participation/Retention 66

4. STATISTICAL ANALYSIS PLAN & POWER 67
4.1. Overview 67
4.2. Primary Analysis for Efficacy 69
4.2.1. Baseline Characteristics 69
4.2.2. Exclusion from Analysis Based on Pre-Enrollment Testing 70
4.2.3. According to Protocol (ATP) Analysis 70
4.2.4. Intent to Treat (ITT) Analysis 70
4.3. Secondary Analyses for Efficacy 70
4.3.1. Duration of Protection 70
4.3.2. Protection against Any Oncogenic HPV Types 71
4.3.3. Protection against HPV16 or HPV18 Associated CIN2+ Among Women with no Evidence of Exposure to HPV16 or HPV18 at Entry 71
4.3.4. Protection against Persistent HPV16 or HPV18 Infection 71
4.3.5. Vaccine Immunogenicity 71
4.4. Evaluation of Adverse Events (AEs) 71
4.5. Other Analyses 72
4.6. Interim Analysis 73
4.7. Power to Determine Vaccine Efficacy 74

5. OVERSIGHT BY THE DATA AND SAFETY MONITORING BOARD & ADVISORY BOARD 76

6. HUMAN SUBJECTS PROTECTION 77

7. VACCINE MANUFACTURING & QUALITY CONTROL 78

8. COLLABORATORS AND CONSULTANTS 79

9. PROJECTED TIMETABLE 80

10. REFERENCES 81

11. APPENDICES 85

3.3.4. Elimination criteria from the ATP analyses during the study 158
3.16.3. Exiting Women from the Trial (Amended: 13 Nov 2008) 159

LIST OF APPENDICES

PAGE

Appendix A CLINICAL MANAGEMENT ALGORITHMS 86

Appendix B RANDOMIZATION PROCEDURES 91

Appendix C HPV PCR TESTING ALGORITHM FOR HPV16 & HPV18 DETERMINATION 93

Appendix D CRITERIA FOR DEFINING ADVERSE EVENTS (AEs), SERIOUS AEs (SAEs), AND PROCEDURES FOR AND TIMING OF AE REPORTING 94

Appendix E HPV16/18 VLP & HAVRIX MSDSs 108

Appendix F PROTOCOL AMENDMENTS AND MODIFICATIONS 122

LIST OF TABLES

PAGE

Table 1 Expected Trial Sample Size (Amended: 13 Nov 2008) 31

Table 2 Timeline of Costa Rican HPV16/18 VLP Vaccine Trial Activities (Amended: 13 Nov 2008) 33

Table 3 Vaccine Dosage and Administration 46

Table 4 Intervals Between Study Visits (Amended: 13 Nov 2008) 47

Table 5 Types of Biological Specimens Proposed and Expected Storage Requirements (Amended: 13 Nov 2008) 60

Table 6 Power to Determine Vaccine Efficacy in the Costa Rican Trial 75

LIST OF FIGURES

PAGE

Figure 1 The Cervical Cancer Continuum 23

Figure 2 Diagrammatic Overview of the Costa Rican HPV16/18 VLP Vaccine Trial 32

Figure 3 Expedited Reporting – Personnel Communication Flow Chart (Amended: 13 Nov 2008) 107

SYNOPSIS

**Title**	A double blind, controlled, randomized, phase III study of the efficacy of an HPV16/18 VLP vaccine in the prevention of advanced cervical intraepithelial neoplasia (CIN2, CIN3, AIS, and invasive cervical cancer) associated with HPV16 or HPV18 cervical infection in healthy young adult women in Costa Rica
**Study population**	Healthy young adult women between and including 18-25 years of age at the time of first vaccination
**Rationale**	Cervical cancer is one of the most common cancers among women worldwide. Approximately 400,000 new cases are diagnosed each year, resulting in about 200,000 deaths. Cervical infection with one of a dozen or so oncogenic human papillomavirus (HPV) types is now known to be the cause of nearly all cervical tumors, with a single HPV type, HPV16, accounting for approximately half of the cases worldwide and an additional type, HPV18 accounting for an additional 10-20% of cases.<br><br>Although cytological screening programs have been in place for several decades, these programs have not been effective at controlling cervical cancer in developing countries, where the vast majority of cervical cancer cases are diagnosed. In developed countries such as the United States, where screening programs have been successful, the costs associated with screening and subsequent colposcopic evaluation and treatment are very high (>$5 billion annually). An effective vaccine against HPV, the virus that causes cervical cancer, is needed.<br><br>Pre-clinical studies have convincingly demonstrated the potential prophylactic efficacy of virus-like particle (VLP) based HPV vaccines discovered at the National Cancer Institute (NCI). The efficacy of the VLP-based vaccines in animal trials has been shown to be due to their correct three-dimensional conformation and to their ability to induce strong neutralizing antibody responses. Human safety and immunogenicity trials conducted in the United States and elsewhere have suggested that VLP-based vaccines are safe and that they induce a strong immune response. Recent data further indicate that vaccination with VLP-based HPV vaccines protects against persistent HPV infections [Koutsky, 2002].<br><br>Based on these promising findings, the NCI and the Foundation for the Costa Rican Institute for Research and Training in Nutrition and Health (FUNIN) plan to conduct a double-blinded, controlled phase III pivotal efficacy trial in Guanacaste, Costa Rica, to evaluate the prophylactic efficacy of the HPV16/18 L1 papillomavirus structural protein VLP-based vaccine manufactured and developed by GlaxoSmithKline (GSK) Biologicals/MedImmune Inc. The vaccine is formulated with investigational adjuvant AS04 (previously SBAS4) comprised of aluminum hydroxide ($Al(OH)_3$) and monophosphoryl lipid A (MPL®).

Rationale (continued)

The trial will enroll approximately 15,000 healthy young women ages 18-25 that will be randomly assigned to one of two arms. Participants will be vaccinated three times intramuscularly over a six month period (i.e., at 0, 1, and 6 months) with a 40 µg dose of the HPV16/18 L1 VLP vaccine or with 720 enzyme-linked immunoabsorbent assay (ELISA) units of GSK Biologicals' (Amended: 29 Mar 2005) hepatitis A vaccine, inactivated (Havrix®) prepared as an investigational formulation. Each woman will be followed with yearly clinic visits and pelvic exams for four years to obtain information on long-term adverse events (AEs) and to determine whether vaccination protects against the development of incident, histopathologically confirmed cervical intraepithelial neoplasia (CIN) 2+ (defined as advanced cervical intraepithelial neoplasia that includes CIN2, CIN3, adenocarcinoma *in situ* [AIS], and invasive cervical cancer) associated with HPV16 or HPV18 cervical infections. The six secondary objectives of this trial are listed below. In keeping with the mission of NCI to produce hypothesis-generating data, numerous exploratory tertiary objectives and ancillary studies are also planned.

Objectives

Primary

To demonstrate efficacy of the candidate vaccine compared with control in the prevention of histopathologically confirmed CIN2+ associated with an HPV16 or HPV18 cervical infection post dose 3 (from Month 6 to Month 48), in young adult women negative for HPV deoxyribonucleic acid (DNA) by polymerase chain reaction (PCR) at Months 0 and 6 for the corresponding HPV type.

CIN2+ is defined as CIN2, CIN3, AIS, or invasive cervical cancer.

Secondary

1. To evaluate the safety of the candidate vaccine in young adult women in the entire randomized cohort, regardless of their initial HPV16/18 DNA (by PCR) status, and also stratified according to initial status. This will be done by strict monitoring and documentation of adverse events.
2. To evaluate the duration of protection conferred by the candidate vaccine against cervical infection with HPV16 or HPV18 post dose 3 (from Month 6 to Month 48), in young adult women negative for HPV DNA (by PCR) at Months 0 and 6 for the corresponding HPV type.
3. To evaluate the efficacy of the candidate vaccine compared with control in the prevention of CIN2+ associated with cervical infection by any oncogenic HPV type, post dose 3 (from Month 6 to Month 48), in young adult women negative for HPV DNA (by PCR) at Months 0 and 6 for the corresponding HPV type.

   CIN2+ is defined as CIN2, CIN3, AIS, or invasive cervical cancer.
4. To evaluate the efficacy of the candidate vaccine compared with control in the prevention of histopathologically confirmed CIN2+ associated with an HPV16 or HPV18 cervical infection post dose 3 (from Month 6 to Month 48) detected within the lesional component of the cervical tissue specimen by PCR, in young adult women negative for HPV DNA (by PCR) at Months 0 and 6 for the corresponding HPV type and seronegative by ELISA at Month 0 for the corresponding HPV type.

CIN2+ is defined as CIN2, CIN3, AIS, or invasive cervical cancer.

Objectives (continued)

5. To evaluate the efficacy of the candidate vaccine compared with control in the prevention of persistent HPV16 or HPV18 cervical infection post dose 3 (from Month 6 to Month 48), in uninfected young adult women negative for HPV DNA (by PCR) at Months 0 and 6 for the corresponding HPV type.

   Persistent HPV16 or HPV18 cervical infection is defined as detection of the same HPV type (by PCR) in cervical samples from all consecutive evaluations over approximately 12 months.

6. To evaluate the vaccine immunogenicity (by ELISA and V5/J4 monoclonal antibody inhibition enzyme immunoassay [EIA]) in a subcohort of (Amended: 19 May 2006) 600 subjects enrolled.

Study Design

Experimental design: Phase III, single center study, active control, with two parallel groups

Study site: Single center with up to five satellite sites. Sites located in one region of Costa Rica (Guanacaste Province and adjacent areas, population ~300,000)

Enrollment: Population-based, ages 18-25, N= ~15,000 (of ~20,000 who will be invited, based on 2000 census data). Randomization of 7,500 women will provide a sufficient study size to evaluate the main trial objectives. (Amended: 13 Nov 2008). The expected enrollment period is 18 (Amended: 31 Oct 2005) months.

Treatment allocation: Randomized (1:1); randomization via sequential numbering of vaccines

Blinding: Double-blind

Treatment groups: Two groups

- **Vaccine**: 40 µg HPV16/18 VLPs/50 µg MPL®/500 µg aluminum as $Al(OH)_3$
- **Control**: Investigational formulation of Havrix® (Hepatitis A Vaccine, Inactivated; Manufactured by GlaxoSmithKline Biologicals, Rixensart, Belgium) [720 ELISA units of antigen; 500 µg $Al(OH)_3$]

Study visits: Average of seven scheduled study visits per subject (8 scheduled visits per subject in the immunogenicity cohort)

Vaccination schedule: Three doses of control or vaccine administered on a 0, 1, 6-month schedule

Cytologic assessments: Cervical liquid-based cytology evaluations at Month 0, 12, 24, 36 and 48

HPV assessments: HPV DNA assessments on cervical cell suspensions collected for cytology at Months 0, 12, 24, 36, and 48. In addition, there will be HPV DNA assessments on cervical cell suspensions collected for cytology at 6 monthly intervals in those women who are being followed for abnormal cytology (see next bullet point). Moreover, all subjects will submit a self-collected cervicovaginal sample at Month 6.

Study Design (continued)

Management of women with abnormal cytology: Women detected as having evidence of a low-grade squamous intraepithelial lesion (LSIL) (regardless of HPV testing results) or atypical squamous cells of undetermined significance (ASC-US) concomitant with detection of an oncogenic HPV type by Hybrid Capture II (HCII) will have cytology performed at 6 monthly intervals (rather than yearly intervals). Those whose ASC-US/oncogenic HPV positive lesion or LSIL persists for 2 evaluations (whether consecutive or intermittent) will be referred for colposcopy for evaluation and excisional treatment, if warranted. Those with 3 consecutive normal cytology evaluations will return to an annual cytology evaluation schedule. For purposes of this management strategy, women with ASC-US whose concomitant specimen tests negative for HPV will be considered normal and followed at annual intervals.

Women detected as having evidence of a high-grade squamous intraepithelial lesion (HSIL), atypical glandular cells (endocervical, endometrial, or glandular; favor neoplastic or not otherwise specified) (AGC), atypical squamous cells, cannot exclude HSIL (ASC-H), or cancer will be immediately referred for colposcopic evaluation and excisional treatment, if warranted.

At the Month 48 visit we will apply the same criteria applied throughout the trial to define women who require colposcopic evaluation. Specifically, all women with evidence of cancer, HSIL, glandular lesions, ASC-H, or persistent ASC-US+/LSIL will be referred to colposcopy. Close-out of individual study participants will only occur after colposcopy referral, for women who require such referral.

It should be noted, that after participant close-out, participants will be offered additional clinical care if needed. Case management after study close-out will be defined under a separate protocol. (Amended: 13 Nov 2008)

Safety assessments:

- In all subjects during the 60-minute (Amended: 31 Oct 2005) post-vaccination period, solicited local AEs (pain, redness, swelling and temperature at the injection site) and solicited general AEs with new onset or aggravation since vaccination (fever, fatigue, myalgia, arthralgia, gastrointestinal symptoms, headache, rash [other than urticaria] and urticaria) will be assessed.
- In 10% of subjects randomly selected, on one day from Day 3 to Day 6 post-vaccination (day of vaccination is Day 0), local and solicited general AEs will be collected during a home visit.
- In all subjects, non-serious AEs that are reported spontaneously or through solicitation at every study visit will be collected throughout the trial.
- In all subjects, serious adverse events (SAEs) and pregnancy outcomes will be collected throughout the trial.

Study Design (continued)

Immunogenicity subcohort: A subcohort (Amended: 19 May 2006) of 600 subjects enrolled, regardless of the study site at which they are enrolled, will have blood samples taken at Months 0, 6, 7, 12, 24, 36 and 48 for immunogenicity assessments. The immunogenicity subcohort will be comprised of these 600 subjects.

Note: All other (Amended: 19 May 2006) subjects enrolled will have blood drawn at Months 0, 6, 12, 24, 36, and 48 (and at Months 18, 30, and 42 if seen at those time intervals) to allow exploratory analyses and ancillary studies by NCI.

Type of study: Self-contained, Investigational New Drug (IND).

Data collection: Conventional (paper) case report form (CRF). In most instances

the CRF will act as a source document.

Duration of the study: A total of 48 months of follow-up per subject is planned for all subjects. Although participants will each be followed for 48 months, final analysis of CIN2+ will be triggered when one of the following set of conditions is met:

- 48 months of follow-up per participant is achieved, or
- At least 30 confirmed cases of CIN2+ associated with HPV16 or HPV18 cervical infection that are eligible for the ATP analysis are observed and the last woman recruited into the study has completed a minimum follow-up period of two years, pending review and approval of the final statistical analysis plan by the DSMB. (Amended: 29 Mar 2005)

Total enrollment was 7,466, which is lower than initial estimates. The final accrual of 7,466 provides 91% power to detect a vaccine efficacy of 80% for the outcome of CIN2+. (Amended: 13 Nov 2008)

- Should analysis occur prior to completion of 48 months of follow-up (under the second scenario above), subjects will continue to be followed for the full 48 months and an annex report will be issued to describe any additional data collected.

Number of Subjects

Target 15,000 subjects enrolled and randomly assigned to one of two arms, 7,500 per group (vaccine/control). 13,000 subjects are expected to be evaluable for the primary According to Protocol (ATP) analysis (6,500 evaluable subjects per group).

Endpoints

Primary

Histopathologically confirmed CIN2+ associated with HPV16 or HPV18 infection detected by PCR in the preceding cervical cytology specimen in young adult women negative for HPV DNA by PCR at Months 0 and 6 for the corresponding HPV type.

CIN2+ is defined as CIN2, CIN3, AIS or invasive cervical cancer.

Preceding cervical cytology means the last cervical cytology specimen collected before the histopathology specimen was obtained.

Secondary

1. Safety

- Occurrence and intensity of solicited local AEs and occurrence (either onset or aggravation) of solicited general AEs including urticaria within 60 (Amended: 31 Oct 2005) minutes after vaccination.
- Occurrence and intensity of solicited local AEs and solicited general AEs on a 10% random subset of participants on one day from Day 3 to Day 6.
- after each vaccination and over all vaccinations combined.
- Occurrence of unsolicited AEs and SAEs throughout the entire study (Month 0 up to Month 48).
- Outcome of all pregnancies.

2. Time to occurrence of incident cervical infection with HPV16 or HPV18 (by PCR).
3. Histopathologically confirmed CIN2+ associated with infection by any oncogenic HPV type (including HPV types 16, 18, 31, 33, 35, 39, 45, 51, 52, 56, 58, 59, 66, and 68), detected by PCR in the preceding cervical

cytology specimen in young adult women negative for HPV DNA (by PCR) at Months 0 and 6 for the corresponding HPV type.

4. Histopathologically confirmed CIN2+ associated with HPV16 or HPV18 infection detected by PCR within the lesional component of the cervical tissue specimen in young adult women negative for HPV DNA (by PCR) at Months 0 and 6 for the corresponding HPV type and seronegative by ELISA at Month 0 for the corresponding HPV type.

5. Persistent HPV16 or HPV18 cervical infection, defined as detection of the same HPV type (by PCR) in cervical samples from all consecutive evaluations over approximately 12 months from Month 6 to Month 48.

(Another way of describing this endpoint is when a subject has 2 or more cervical cytology specimens positive for the same HPV type, either HPV16 or HPV18, over an approximately 12-month period without any intervening negative specimen. The term approximately is used because ATP visit intervals may vary as shown in Table 4 in Section 3.11).

6. HPV16 and HPV18 ELISA and V5/J4 monoclonal antibody inhibition EIA titers in the 600 subjects enrolled into the immunogenicity subcohort. (Amended: 19 May 2006)

1. INTRODUCTION

1.1. Background

DCEG has a longstanding interest in studies of HPV and cervical neoplasia. In the 1980's two case-control studies were conducted, one in the United States and one in Latin America, to examine risk factors associated with cervical cancer. The Latin American study was the first large-scale epidemiological study to demonstrate an association between HPV infection and cervical cancer [Reeves, 1989]. Subsequently, three cohort studies (two in the United States and one in Costa Rica) were established to examine the natural history of HPV and cervical neoplasia [Schiffman, 1993, Herrero, 2000, The Atypical Squamous Cells of Undetermined Significance/Low-Grade Squamous Intraepithelial Lesions Triage Study (ALTS) Group, 2000]. These studies have jointly provided critical data linking HPV infection to the development of cervical cancer and its precursor lesions (known cytologically as LSIL for low-grade squamous intraepithelial lesions and HSIL for high-grade SIL; and known histopathologically as condyloma and CIN1-3 for cervical intraepithelial neoplasia grades 1-3 – see Figure 1). In fact, it is now understood that LSIL is the cytological manifestation of HPV infection [Schiffman, 1998]. It is also known that while the majority of HPV infections are self-limited, persistent infection with one of about 15 oncogenic HPV types is required for the development of nearly all cases of cervical cancer and its immediate precursor, HSIL [Herrero, 2000, Munoz, 2003, Bosch, 1995]. HPV16 is the most important type, accounting for about half of all cancer cases, while HPV18 accounts for another 10-20% of all cases [Bosch, 1995]. Available evidence suggests that infections with different HPV types are independent and that infection with one type does not modulate infections with other HPV types [Herrero, 2000, Liaw, 2001]. Prevention of CIN and cervical cancer (one of the most common cancers among women worldwide) is therefore theoretically possible via prevention of oncogenic HPV infection and/or its associated disease.

One of the cohort studies sponsored by DCEG is being conducted in Guanacaste, Costa Rica, among 10,000 adult women [Herrero, 2000, Herrero, 1997, Schiffman, 1999]. Started in 1993, this population-based cohort was designed to elucidate the origins of HSIL and cervical cancer. Response rates during the enrollment phase of this study were 93% and over the past seven years close to 95% of women selected for active follow-up have continued to participate. This makes it an attractive region in which to evaluate the efficacy of a vaccine developed to prevent HPV infection and SIL, not only because of the high compliance of this rural population, but also because of the high rates of cervical neoplasia in Guanacaste (despite rates of HPV infection comparable to those in the US – a phenomenon likely due to differences between the two countries in screening frequency and quality) and our understanding of the expected distribution and rates of HPV infections and cervical neoplasia.

Figure 1 The Cervical Cancer Continuum

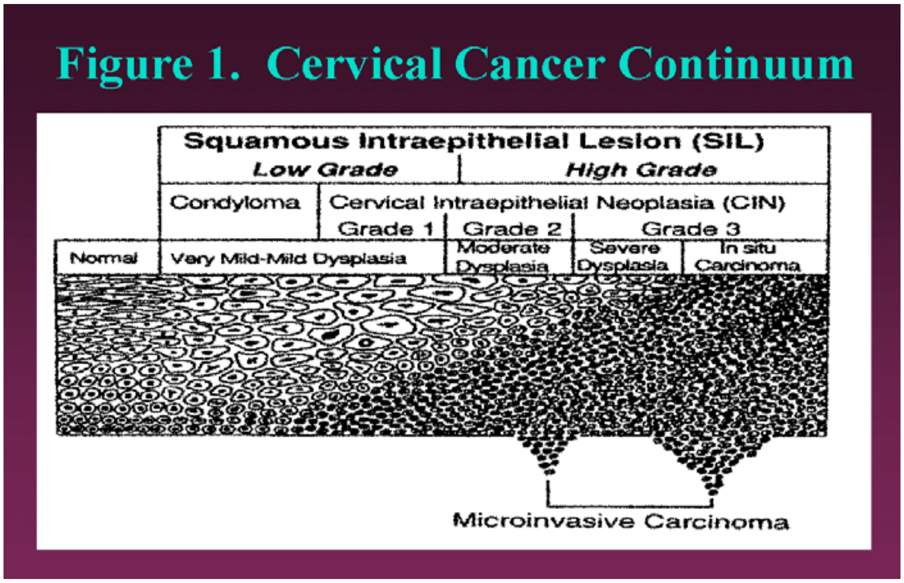

In parallel with the epidemiological work conducted by DCEG in the 1980's and 90's, investigators in the Center for Cancer Research (CCR) at NCI developed an *in vitro* method to produce papillomavirus structural proteins (L1 proteins) capable of self-assembling into viral particles (called L1 virus-like particles or L1 VLPs) [Kirnbauer, 1994]. These particles consist of the outer coat of the virus in its correct three-dimensional configuration but are free of nucleic acids and are therefore noninfectious. When used as vaccines in animal studies, L1 VLPs were shown to induce high levels of neutralizing antibodies against the papillomavirus L1 structural protein and to protect against direct experimental challenge with the virus [Breitburd, 1995, Kirnbauer, 1996, Suzich, 1995]. It has further been demonstrated that protective antibody levels are seen for at least one year following vaccination and that systemic inoculation induces not only circulating antibodies but also antibodies in the genital tract [Christensen, 1996, Lowe, 1997]. There are currently three animal models (rabbits, cattle, and dogs) supporting a prophylactic effect of vaccination with papillomavirus L1 VLP. VLP antibody-mediated protection from infection was type-specific both in the animal vaccine studies and in *in vitro* assays using HPV VLPs.

Based on these initial pre-clinical studies, safety and immunogenicity studies were initiated in humans. To date, over 3,000 individuals in the United States, Brazil and elsewhere have been vaccinated with a VLP-based vaccine, with very promising results. VLP-based vaccines manufactured by three groups have been tested in humans. These include an HPV16 L1 VLP vaccine manufactured by Novavax, Inc. under contract with the NCI, HPV16 and HPV6/11/16/18 L1 VLP vaccines manufactured by Merck Pharmaceuticals, and HPV11 and HPV16/18 L1 VLP vaccines manufactured by MedImmune, in partnership with GlaxoSmithKline (GSK) Biologicals. HPV16 has been

the primary target of these vaccines since it is known to be associated with half of all cervical cancer cases detected worldwide. HPV18, the other oncogenic HPV type included in some of the vaccines tested to date accounts for an additional 10-20% of cervical cancer cases worldwide. HPV types 6 and 11 are non-oncogenic HPV types that are responsible for genital warts. The presence of these two types should not impact cervical cancer rates.

Without exception, results from early phase human trials have indicated that VLP-based vaccines are well tolerated and that they induce high levels of neutralizing antibodies [Koutsky, 2002, Harro, 2001, Evans, 2001]. Antibodies have been detected both systemically and in the genital tract of vaccinated women [Nardelli-Haefliger, 2003]. Furthermore, initial results from a phase IIb trial of an HPV16 VLP vaccine conducted by Merck Pharmaceuticals has recently demonstrated that vaccination effectively protects against 6 month persistent infection with HPV16 with a high level of efficacy [Koutsky, 2002]. Results from a recently completed Phase IIb trial conducted by GSK Biologicals confirm the finding and extend it to HPV18. (Amended: 29 Mar 2005) Although these results are very promising, data showing protection against the development of cervical cancer precursor lesions (known as HSIL cytologically and CIN2-3 histopathologically) is needed.

The NCI initially tested an HPV16 VLP vaccine manufactured under contract by Novavax, Inc. but found scale-up of vaccine manufacturing under GMP conditions not to be feasible in a timely manner. Also, the vaccines manufactured by the larger pharmaceutical companies contained two oncogenic HPV types (HPV16 and HPV18) and were therefore likely to be superior to the monovalent vaccine being manufactured by Novavax, Inc. GSK Biologicals has agreed to provide their bivalent HPV16/18 VLP vaccine under a Clinical Trials Agreement with NCI, and we therefore plan to test this vaccine in the Costa Rican efficacy trial.

Safety and immunogenicity results from early phase trials of the HPV16/18 VLP vaccine manufactured by MedImmune/GSK Biologicals are described in the Investigator Brochure provided under separate cover (Investigator Brochure for the Candidate Prophylactic HPV16/18 VLP Vaccine Formulated with AS04, GSK-580299). Phase I and II studies have been conducted under the supervision of the US FDA to investigate the safety, reactogenicity, and immunogenicity of the HPV16/18 VLP vaccine candidate. In these trials, over 700 healthy women received at least one dose of the HPV16/18 VLP vaccine. The antigen concentration and adjuvant for the vaccine formulation advanced to phase III development was selected mainly based on these clinical studies. In summary, the vaccine at all antigen concentrations and formulations (with or without various adjuvants), when administered as 3 doses over 6 months, was well-tolerated and induced potent immune responses, measured as neutralizing antibodies against HPV16 and HPV18. A formulation containing 20 μg of each of the two HPV types (40 μg total) plus the adjuvants aluminum hydroxide ($Al(OH)_3$) and 3-deacylated monophosphoryl lipid A (MPL®) (in combination designated AS04) elicited the highest geometric mean titers of virus-specific antibody while retaining a favorable reactogenicity profile. This vaccine candidate was used in a recently completed phase IIb trial and will be used in the Costa Rican phase III efficacy trial.

As indicated above, the candidate HPV16/18 VLP vaccine that will be used in our trial is formulated with an investigational adjuvant (AS04). AS04 is an adjuvant that is not used in any licensed vaccines. Nevertheless, GSK Biologicals has compiled considerable information regarding its safety during clinical trials of other vaccines. The information in this section reflects knowledge that was current at the time of study start. Please refer to the most recent edition of the Investigator Brochure for a review of clinical and pre-clinical studies (Amended: 13 Nov 2008).

1.2. The Need for an HPV Vaccine

Cervical cancer is the second or third most common female tumor worldwide [Parkin, 1999]. In many developing countries, where rates are highest, cervical cancer is the most common malignancy diagnosed among women. Over 400,000 new cases of cervical cancer are diagnosed each year, resulting in approximately 200,000 deaths. While Pap smear screening programs in developed nations have been shown to be effective at reducing the incidence of invasive cervical cancer, difficulties remain in providing underserved populations within these developed countries with access to routine cytological screening programs. Also, in developing countries, it has proven difficult to implement effective screening programs despite numerous attempts over the past 30+ years. In Costa Rica, for example, rates of disease have remained high despite screening of approximately 80% of adult women in the country [Herrero, 1993]. Furthermore, even in developed nations, where cervical cancer rates have dropped significantly since Pap smear screening programs were implemented, the cost associated with these efforts is large. As an example, in the United States, the cost associated with Pap smear screening and subsequent colposcopic follow-up and treatment is greater than five billion dollars annually [Kurman, 1994]. An effective HPV vaccine could, in the long run, prove to be a more effective way to reduce cervical cancer incidence and mortality in developing countries, and also to reduce the costs associated with cervical cancer prevention efforts in the United States and other developed and industrialized nations.

1.3. Defining Cervical Abnormalities

Before describing the proposed efficacy trial itself, it is important that we carefully define the terminology used to classify women with respect to degree of cervical abnormality. Based on our understanding of the natural history of cervical cancer, we know that cervical cancer begins with infection with one of about 15 oncogenic HPV types [Munoz, 2003, IARC Working Group on the Evaluation of Carcinogenic Risks to Humans, 1995]. As mentioned above, most of these HPV infections are transient but a small proportion of infections persist and are at risk of progressing to more severe disease [Hildesheim, 1994, Ho, 1995, Nobbenhuis, 1999].

Using the latest cytological Bethesda 2001 classification system [Solomon, 2002], the first unequivocal cytological evidence of HPV infection is called LSIL (low-grade squamous intraepithelial lesions). Careful studies have demonstrated that 85+% of LSIL lesions are HPV positive for oncogenic types, and so while there may be a small subset of LSIL diagnoses that represent “look-a-likes”, the vast majority of LSIL lesions represent true HPV-related disease [The Atypical Squamous Cells of Undetermined

Significance/Low-Grade Squamous Intraepithelial Lesions Triage Study (ALTS) Group, 2000].

In addition to these LSIL lesions, equivocal lesions (called ASC-US for atypical squamous cells of undetermined significance) are commonly diagnosed. It is now possible to classify ASC-US lesions into the benign reactive atypias or true SILs by using oncogenic HPV testing [Solomon, 2001].

Persistent oncogenic HPV infections can progress to more worrisome high-grade disease known as high-grade squamous intraepithelial lesions (HSIL), usually within a period of many years (5-10 years, on average) [IARC Working Group on the Evaluation of Carcinogenic Risks to Humans, 1995]. HSIL, a cytological diagnosis, comprises the histopathological diagnoses of CIN2 and CIN3. These lesions are often, but not always, preceded by abnormal cytology [IARC Working Group on the Evaluation of Carcinogenic Risks to Humans, 1995, Koutsky, 1992]. These high-grade lesions, which include carcinoma *in situ* (CIS), are the immediate precursors to cervical cancer.

Based on this understanding of the natural history of cervical cancer, we propose to use the following cytological classification to guide clinical management of participants in our trial:

1. Normal: Normal cytology (including benign reactive changes) or atypical squamous cells of undetermined significance (ASC-US) that tests negative for oncogenic HPV types.
2. ASC-US+/LSIL: LSIL by cytology or ASC-US that tests positive for oncogenic HPV types.
3. HSIL: HSIL by cytology. Atypical squamous cells, cannot exclude HSIL (ASC-H) will be included in this group due to their high risk of underlying HSIL. Atypical glandular cells (AGC) and AIS, rare forms of cervical glandular abnormalities, will also be included in this group due to their high risk of progression.
4. Cancer: Cancer by cytology (very few, if any, cancers are expected in our cohort of young women).

Additional details regarding our clinical management plan are provided in Section 3.15

For purposes of defining endpoints for our primary and secondary analyses, histopathological criteria will be used. Only histopathologically confirmed CIN2+ will be considered a true outcome for analysis purposes. Additional details regarding our definition of trial endpoints are provided in Section 3.13.

1.4. Summary

We now know that HPV is involved in the pathogenesis of nearly all cervical tumors and that HPV16 and HPV18 combined account for 60% or more of all cervical cancer cases worldwide. Pre-clinical and safety/immunogenicity human trials (in >3,000 participants) have shown that vaccination against papillomaviruses using VLP-based vaccines protects against infection and tumor development in animals, and that the vaccines are both safe

and immunogenic in humans. Results from proof-of-principle trials also indicate that VLP-based vaccines are effective at protecting against persistent infection in humans. While alternative methods exist to control cervical cancer (i.e., Pap smear screening), these methods have been unsuccessful in developing countries. In the few developed countries in which screening has been successful, the costs associated with these efforts are high. The development of an HPV vaccine that protects against the development of cervical cancer and its precursors is therefore desirable. We propose to conduct a pivotal efficacy trial to evaluate the effectiveness of the HPV16/18 VLP-based vaccine manufactured by GSK Biologicals to protect against the development of histopathologically confirmed, incident CIN2+ associated with either HPV16 or HPV18 infections. The trial will be conducted in Guanacaste, Costa Rica, an area with high rates of cervical cancer and the site of an ongoing NCI-sponsored 10,000-woman, population-based natural history study.

2. STUDY OBJECTIVES

2.1. Primary Objective

The main objective of the present randomized clinical trial is to:

Demonstrate efficacy of the candidate vaccine compared with control in the prevention of histopathologically confirmed CIN2+ associated with an HPV16 or HPV18 cervical infection post dose 3 (from month 6 to month 48), in young adult women negative for HPV DNA (by PCR) at months 0 and 6 for the corresponding HPV type.

CIN2+ is defined as CIN2, CIN3, AIS, or invasive cervical cancer. Refer to Section 3.13.1 for the definition of the primary endpoint.

2.2. Secondary Objectives

In addition to the primary objective listed above, this trial will address a few selective secondary objectives of importance to determine potential clinical uses of the vaccine. These secondary objectives are to:

1. Evaluate the safety of the candidate vaccine in young adult women in the entire randomized cohort, regardless of their initial HPV16/18 DNA (by PCR) status, and also stratified according to initial status. This will be done by strict monitoring and documentation of adverse events.
2. Evaluate the duration of protection conferred by the candidate vaccine against cervical infection with HPV16 or HPV18 post dose 3 (from month 6 to month 48), in young adult women negative for HPV DNA (by PCR) at months 0 and 6 for the corresponding HPV type.
3. Evaluate the efficacy of the candidate vaccine compared with control in the prevention of CIN2+ associated with cervical infection by any oncogenic HPV type, post dose 3 (from month 6 to month 48), in young adult women negative for HPV DNA (by PCR) at months 0 and 6 for the corresponding HPV type.

CIN2+ is defined as CIN2, CIN3, AIS or invasive cervical cancer.

4. Demonstrate efficacy of the candidate vaccine compared with control in the prevention of histopathologically confirmed CIN2+ associated with an HPV16 or HPV18 cervical infection post dose 3 (from month 6 to month 48) detected within the lesional component of the cervical tissue specimen by PCR, in young adult women negative for HPV DNA (by PCR) at months 0 and 6 for the corresponding HPV type and seronegative by ELISA at month 0 for the corresponding HPV type.

   CIN2+ is defined as CIN2, CIN3, AIS, or invasive cervical cancer.
5. Evaluate the efficacy of the candidate vaccine compared with control in the prevention of persistent HPV16 or HPV18 cervical infection post dose 3 (from month 6 to month 48), in young adult women negative for HPV DNA (by PCR) at months 0 and 6 for the corresponding HPV type.

   Persistent HPV16 or HPV18 cervical infection is defined as detection of the same HPV type (by PCR) in cervical samples from all consecutive evaluations over approximately 12 months from Month 6 to Month 48.
6. Evaluate the vaccine immunogenicity (by ELISA and V5/J4 monoclonal antibody inhibition EIA) in a subset of (Amended: 19 May 2006) 600 subjects enrolled (immunogenicity subcohort).

Refer to Section 3.13.2 for definitions of the secondary endpoints.

2.3. Tertiary Objectives

The NCI and Costa Rican researchers are committed to addressing several other objectives of interest to the scientific community. While a comprehensive listing of all tertiary objectives and analyses is beyond the scope of this protocol, to follow is a summary of some of the topics that are being considered for exploratory evaluation by NCI and Costa Rica. [Note: It is understood that the power to address some of these objectives may be limited].

1. An evaluation of alternative histological, cytological, virological, and immunological outcomes that could help us understand the mechanisms of vaccine action, and that might be used as surrogate endpoints in future studies.
2. An evaluation of the therapeutic efficacy (secondary prevention) of the candidate vaccine.
3. An evaluation of vaccine efficacy in subsets of women defined based on their socio-demographic and risk factor characteristics.
4. An evaluation of the effect of vaccination on the distribution of HPV types and variants in our population.
5. An evaluation of vaccine efficacy of the candidate vaccine against HPV infections that occur at extra-cervical sites. (Amended: 13 Nov 2008)

Refer to Section 3.13.1 for definitions of tertiary endpoints.

2.4. Ancillary Analyses & Studies

The present clinical trial provides a unique opportunity for NCI, Costa Rican, and other investigators to answer various other immunological and natural history questions using information and materials collected as part of this trial. Opportunities might also arise to conduct ancillary studies that require the collection of additional information and/or biological specimens not planned for as part of the current investigation. Although the exact nature of these ancillary analyses and studies is currently unknown, we plan to set up a mechanism by which investigators can propose to perform ancillary studies within the context of our trial. These ancillary studies will be judged on their scientific merit and on their potential impact on the primary and secondary objectives of our trial. Efforts will be made to ensure that no ancillary study interferes with our ability to successfully evaluate the primary and secondary objectives listed above.

Any ancillary study or protocol requiring contacts with the participants not described in the current protocol or requiring collection of additional specimens will be submitted for review by the IRBs involved.

Examples of ancillary analyses and studies being considered at present include:

1. A family-based study to examine host genetic factors involved in the immunological response to HPV vaccination.
2. A nested case-control study to evaluate the risk factors associated with the development of “rapid onset” HSIL among young women.
3. A longitudinal study of women who are sexually inexperienced (virginal) at study entry and who subsequently become sexually active, to examine the early natural history of HPV infection in unvaccinated women and the host immune response to these early infections.

3. METHODS

A detailed operating procedures manual called the “Field Procedures Manual” has been written for this study. The Field Procedures Manual has been reviewed to assure that it reflects the procedures described in this protocol. The investigators, study staff, and site monitors will be trained to use the Field Procedures Manual.

3.1. Overview of the Trial

Approximately 20,000 women will be invited to participate in a double blinded, randomized clinical trial to evaluate whether vaccination with the bivalent HPV16/18 VLP-based vaccine manufactured by GSK Biologicals protects against the development of histopathologically confirmed, incident CIN2+. Women between 18-25 years of age at entry who are residents of Guanacaste Province or surrounding areas will be eligible. Participants will be required to not be pregnant at the time of vaccination, to agree to use an effective birth control method 30 days before vaccination until 60 days after the last vaccination (approximately 9 months total), and to be in good health as determined by an overall physical examination administered by a study physician at entry into the trial.

Women will be identified through a census of the region performed in February/July 2000, and will be invited to participate in the trial by visiting one of the study clinics set up specifically for this trial. While randomization (into HPV or HAV vaccination) of 15,000 women was initially anticipated, randomization of 7,500 women will provide a sufficient study size to evaluate the main trial objectives based on revised statistical assumptions, see Section 4.7. (Amended: 13 Nov 2008)

Selected women who are eligible and agree to participate will be given a pelvic examination (if they report previous sexual activity) to collect baseline cervical specimens prior to vaccination, and will be administered 3 doses of a 0.5 mL vaccine containing either HAV (control vaccine) or 40 μg HPV16/18 VLP (20 μg each of HPV16 VLP and HPV18 VLP; VLP vaccine). The initial vaccination will be performed at entry, and additional vaccine doses will be given at one and six months following the initial vaccination. Women will be monitored at the clinic for a minimum of 60 (Amended: 31 Oct 2005) minutes following each vaccination, during which study personnel will collect information on reactogenicity of the vaccine. Appropriate medical treatment will be readily available in case of a rare anaphylactic reaction. A home visit in the week after each vaccination will also be performed by a specially-trained outreach worker on a 10% random sample of participants, to collect additional information on vaccine side effects. A toll-free number will be staffed throughout the duration of the trial so that volunteers may contact the vaccine trial clinical team at any time in case of an emergency.

Long-term follow-up will include clinic visits at yearly intervals starting on the first anniversary of vaccination and continuing through the fourth year anniversary. Follow-up visits will include a pelvic examination (among sexually experienced women) to permit a careful evaluation of the cervix and determination of HPV and cytological status.

Questionnaires and biological specimens will be obtained at the enrollment and follow-up visits. The questionnaire will elicit information on risk factors thought to be associated with HPV infection, SIL, or immune response to vaccination. Biological specimens collected during the trial will include blood (plasma, serum, red blood cells, and buffy coat on all participants, and cryopreserved PBMCs on a subset of participants), cervical cells used for cytology and HPV DNA testing and other research assays, and cervical secretions used to measure levels of mucosal antibodies and other immunological/inflammatory parameters.

Each volunteer is expected to have, on average, seven clinic visits (three vaccination visits and four annual follow-up visits). A 10% random sample of women will also have three home visits performed to monitor vaccine side effects. A subset of 600 women will have a visit at month 7 to assess immunogenicity a minimum of 30 days following the last vaccination. Women diagnosed with LSIL or HPV positive ASC-US will be scheduled for clinic visits with pelvic examinations at six-month intervals to assure adequate clinical management. Women with evidence of HSIL, ASC-H, AGC (Amended: 29 Mar 2005) or persistent ASC-US+/LSIL at any time during the study will be referred for colposcopic evaluation and subsequent excisional treatment, if needed. Treatment standards will be consistent with those in the United States and Costa Rica.

To assist the reader, three summary tables/figures are provided. The first summarizes the expected number of women identified, enrolled, vaccinated and followed as part of our trial (Table 1). The second is an overview diagram of the trial (Figure 2). The third is a table depicting the timeline and procedures planned for the trial (Table 2).

Table 1 Expected Trial Sample Size (Amended: 13 Nov 2008)

Number of women (from census) invited to participate	19,000 – 21,000
Number of eligible women who agree to be randomly assigned to one of two study arms	7,500
Number of women who will be randomly assigned to one of two study arms, will receive 3 doses of vaccine and will be evaluable in ATP analysis	5,300
Estimated number of women who complete four years of follow-up	6,375

Figure 2 Diagrammatic Overview of the Costa Rican HPV16/18 VLP Vaccine Trial

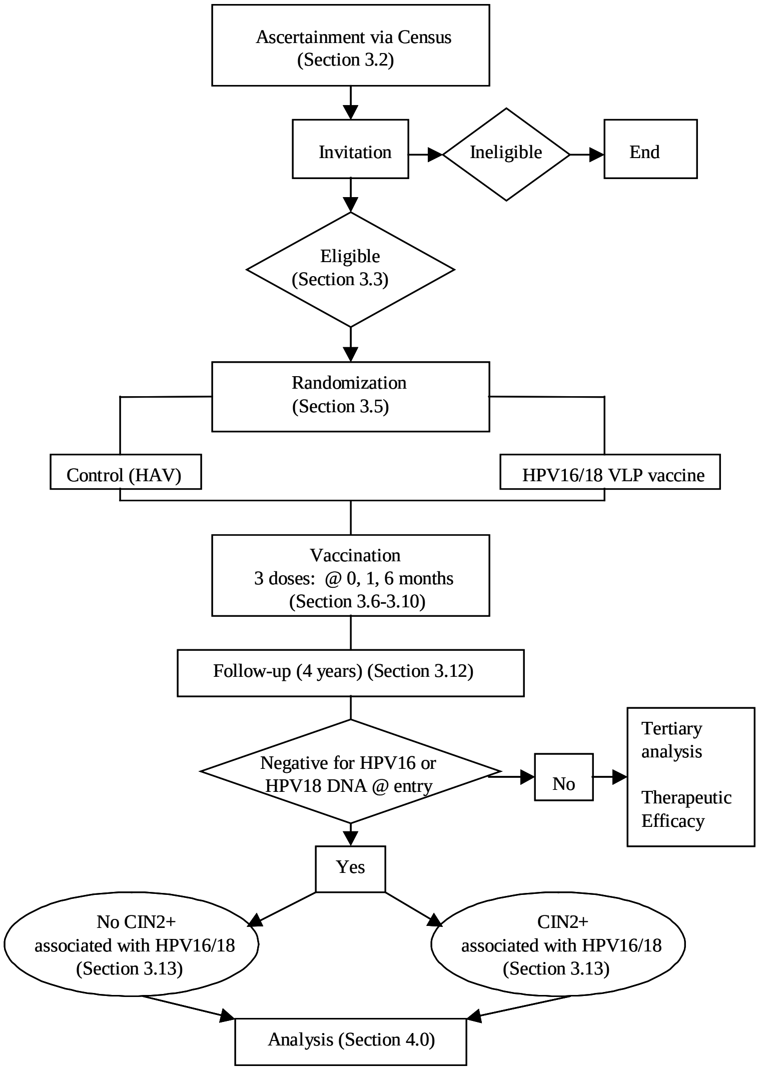

Note: There is only one set of study procedures, however, the analyses will vary depending on the initial HPV DNA status of the subject.

Table 2 Timeline of Costa Rican HPV16/18 VLP Vaccine Trial Activities (Amended: 13 Nov 2008)

	Invitation	Vaccination and AE monitoring			Follow-up							
Timing of Home Visit/Office Visit/Contact	-1 month	0 month	1 month	6 months	7 months*	12 months	18 months**	24 months	30 months**	36 months	42 months**	48 months
Desired & [Maximum Allowable] Range in Days relative to Month 0 Visit	-30 to –3	0	21 to 62 [21 to 120]	161 to 216 [121 to 300]	30 to 60 after 6 month visit	301 to 480	481 to 660	661 to 840	841 to 1020	1021 to 1200	1201 to 1380	1381 to 1740#
Type of Visit/Contact	Invitation	Enrolment & Vaccine #1	One Month & Vaccine #2	Six Month & Vaccine #3	Immuno-genicity Subset	1st Fup	Interim Fup	2nd Fup	Interim Fup	3rd Fup	Interim Fup	4th Fup
Sampling timepoint		Pre	PV I	PV II	PV III	PV III	PV III	PV III	PV III	PV III	PV III	PV III
Contact potential subjects by mail	●											
Visit potential subjects at their homes and provide them with a copy of the informed consent form	●											
Informed consent		●										
Check eligibility/inclusion criteria		●										
Check exclusion criteria		●										
Check vaccination deferral criteria		●	●	●								
Check elimination criteria		●	●	●	●	●	●	●	●	●	●	●
Check contraindications		●	●	●								
Interview (Questionnaire)		●		●		●	●	●	●	●	●	●
Complete medical history		●										
Interim medical history			●	●	●	●	●	●	●	●	●	●
Physical examination		●										
Pre-vaccination body temperature		●	●	●								
Urine sampling for urine pregnancy test (50 mL)		●	●	●								
Randomization		●										
Blood sampling												

	Invitation	Vaccination and AE monitoring			Follow-up							
Timing of Home Visit/Office Visit/Contact	-1 month	0 month	1 month	6 months	7 months*	12 months	18 months**	24 months	30 months**	36 months	42 months**	48 months
Desired & [Maximum Allowable] Range in Days relative to Month 0 Visit	-30 to –3	0	21 to 62<br>[21 to 120]	161 to 216<br>[121 to 300]	30 to 60 after 6 month visit	301 to 480	481 to 660	661 to 840	841 to 1020	1021 to 1200	1201 to 1380	1381 to 1740#
Type of Visit/Contact	Invitation	Enrolment & Vaccine #1	One Month & Vaccine #2	Six Month & Vaccine #3	Immuno-genicity Subset	1st Fup	Interim Fup	2nd Fup	Interim Fup	3rd Fup	Interim Fup	4th Fup
Sampling timepoint		Pre	PV I	PV II	PV III	PV III	PV III	PV III	PV III	PV III	PV III	PV III
For antibody test/research (10 mL)<br>For hematocrit (3 mL)<br>For research (10 mL)<br>For cryopreservation (40 mL)		●<br>●<br>●<br>●***	●	●<br><br><br>●***	●<br><br><br>●***	●<br><br><br>●***	●<br><br><br>●***	●<br><br><br>●***	●<br><br><br>●***	●<br><br><br>●***	●<br><br><br>●***	●<br><br>●<br>●***
Pelvic examination†		●		●††		●	●	●	●	●	●	●
Collection of cervical secretions for research†		●		●††		●	●	●	●	●	●	●
Collection of cervical sample for:<br>-cervical cytology,<br>-HPV PCR testing, and<br>-HCII testing†¶		●§		●††		●	●††	●	●††	●	●††	●^
Collection of subject-obtained cervical sample for HPV PCR testing†				●								
Collection of cervical cells for research†		●		●††		●	●	●	●	●	●	●
Vaccination		●	●	●								
Monitoring for solicited & unsolicited symptoms 60 min post-vaccination		●	●	●								

	Invitation	Vaccination and AE monitoring			Follow-up							
Timing of Home Visit/Office Visit/Contact	-1 month	0 month	1 month	6 months	7 months*	12 months	18 months**	24 months	30 months**	36 months	42 months**	48 months
Desired & [Maximum Allowable] Range in Days relative to Month 0 Visit	-30 to –3	0	21 to 62 [21 to 120]	161 to 216 [121 to 300]	30 to 60 after 6 month visit	301 to 480	481 to 660	661 to 840	841 to 1020	1021 to 1200	1201 to 1380	1381 to 1740#
Type of Visit/Contact	Invitation	Enrolment & Vaccine #1	One Month & Vaccine #2	Six Month & Vaccine #3	Immuno-genicity Subset	1st Fup	Interim Fup	2nd Fup	Interim Fup	3rd Fup	Interim Fup	4th Fup
Sampling timepoint		Pre	PV I	PV II	PV III	PV III	PV III	PV III	PV III	PV III	PV III	PV III
On one day from Day 3-6 after vaccination, a specially-trained outreach worker will interview a random subset of 10% of the 15,000 subjects for follow-up regarding post-vaccination solicited & unsolicited symptoms		●	●	●								
Recording of AEs (nonserious and serious) occurring during the period since the last visit			●	●	●	●	●	●	●	●	●	●
Recording of any concomitant medication/vaccination taken in the 30 days prior to SAE onset that could have contributed to the SAE		●	●	●	●	●	●	●	●	●	●	●
Recording of any concomitant medication/vaccination used to treat an SAE, that clarifies the diagnosis of the SAE or is an immunosuppressant		●	●	●	●	●	●	●	●	●	●	●
Reporting of all pregnancies and all pregnancy outcome information		●	●	●	●	●	●	●	●	●	●	●
Study Conclusion												●

	Invitation	Vaccination and AE monitoring			Follow-up							
Timing of Home Visit/Office Visit/Contact	-1 month	0 month	1 month	6 months	7 months*	12 months	18 months**	24 months	30 months**	36 months	42 months**	48 months
Desired & [Maximum Allowable] Range in Days relative to Month 0 Visit	-30 to –3	0	21 to 62 [21 to 120]	161 to 216 [121 to 300]	30 to 60 after 6 month visit	301 to 480	481 to 660	661 to 840	841 to 1020	1021 to 1200	1201 to 1380	1381 to 1740#
Type of Visit/Contact	Invitation	Enrolment & Vaccine #1	One Month & Vaccine #2	Six Month & Vaccine #3	Immuno-genicity Subset	1st Fup	Interim Fup	2nd Fup	Interim Fup	3rd Fup	Interim Fup	4th Fup
Sampling timepoint		Pre	PV I	PV II	PV III	PV III	PV III	PV III	PV III	PV III	PV III	PV III
Collection of Oral HPV Samples												●
Collection of Anal HPV Samples												●
Collection of Vulvar HPV Samples												●

*7 month visit applies only to the 600 women enrolled in the immunogenicity subcohort. (Amended: 19 May 2006)

**Visit is only required if indicated by the case management algorithm. The protocol-specified clinical management algorithm is summarized in Appendix A.

Fup = follow-up; Pre = pre-vaccination; PV = post-vaccination. ● is used to indicate a study procedure that requires documentation in the individual CRF.

Note: The double-line borders following Months 0, 1 and 6 indicate that a home visit by a specially-trained outreach worker to monitor AEs will occur during the week following vaccination (any day from Day 3 to Day 6) in a random subset of 10% of the women.

***This blood draw will be performed on a subset of women, and at selected study visits, as follows: A) A random sample of women at 0, 12, and 36 months; B) All women referred to accelerated screening visits per management algorithm, at yearly intervals *before March 31, 2009 (Amended: 10 September 2009)*; C) All women referred for colposcopic evaluation who require a biopsy or LEEP, at the time of the colposcopy visit; D) women enrolled in the immunogenicity subcohort after March 1, 2006. (Amended: 19 May 2006).

†Procedure will not be performed on virginal women.

††Pelvic examination will be performed if indicated by the case management algorithm. The protocol-specified clinical management algorithm is summarized in Appendix A.

¶Reflex HCII HPV testing will be performed automatically for all subjects with results of ASC. HCII HPV testing will also be performed on all women who have a pelvic examination at the 0 month visit.

§HCII testing for *Chlamydia trachomatis* and *Neisseria gonorrhoeae* will be performed on samples obtained at the 0 month visit (or the first visit with a pelvic examination for women who are virginal at entry) only.

^ HPV testing by HCII will be performed on all specimens obtained at the 48 month visit.

#Every effort will be made to schedule study participants within the preferred Month 48 study visit window (1381 to 1740 days post Month 0). However, the window for the last visit might be extended until October 3, 2010, which is the scheduled date of last subject last visit (i.e, 1740 days after the 4 year anniversary date for the last subject first visit).

3.2. Study Population

The trial will be conducted in the Guanacaste Province of Costa Rica and surrounding areas. Potential participants were initially identified through a door-to-door census of women in the region. The census was performed by a professional survey team in February/July 2000 and obtained the following information on all female residents between the ages of 12 and 22:

- Complete name
- Date of birth
- National ID number (if available)
- Nationality
- Address
- Telephone number (if available)
- Contact person outside the household.

While women recruited into our trial will be 18-25 years of age at enrollment (see eligibility criteria below), the census enumerated women aged 12-22. This was done to account for the lag time between the census and the initiation of the trial, and to allow for the possibility of extending enrollment if necessary to achieve the desired sample size. If the number of women to be invited is modified, approval will be sought by the IRBs reviewing the trial.

3.3. Eligibility Criteria

3.3.1. Inclusion Criteria

To be eligible for participation in our trial, participants must fulfill all of the following inclusion criteria:

- Gender: Female
- Age: 18 to 25 years (inclusive) at the time of first vaccination (i.e., enrollment)
- Planned residence: Guanacaste Province and surroundings (including parts of Puntarenas and Alajuela Provinces) for the 6 months following first vaccination
- Language: Able to speak/understand Spanish
- Mental Competence: Appears to be mentally competent
- Health Status: In good general health, as determined by a physical examination and history performed by a study physician at enrollment
- Consent: Written informed consent obtained prior to enrollment

We have chosen to restrict the trial to women since the prevalence of detectable HPV infection and clinical outcomes is higher and more easily and reliably detectable in women than in men. It is understood, however, that should the vaccine prove effective, vaccination programs might include both males and females in order to achieve "herd immunity". Separate trials will be required to evaluate the vaccine in men.

Our trial is further restricted to young, adult women (18-25 years) to maximize the number of participants unexposed to HPV at entry who are likely to be exposed to the virus during the follow-up period. We know from natural history studies in Guanacaste and elsewhere that prevalence of HPV peaks in the late teens and early 20s, following initiation of sexual activity, and that LSIL rates peak approximately 1-2 years later.

Restriction of our trial to the Guanacaste Province and surroundings was dictated by our understanding of this population, their cooperativeness in previous studies, and the known high rates of cervical cancer in this area. The high rates of disease in this population maximize the potential benefit to individual participants, should the vaccine prove effective. Women with definite plans to out-migrate will not be eligible to avoid loss to follow-up, since this could introduce bias into our trial.

Women who cannot speak or understand Spanish and those who are mentally incompetent will not be included in our trial because of concerns that they may not be capable of providing adequate informed consent.

Finally, to assure safety of women enrolled in our trial, we plan to collect medical history information and to perform a physical and pelvic examination (if sexually experienced) on all women at the time of their enrollment into the study. Women with a history of chronic conditions requiring treatment (such as cancer, chronic hepatic or kidney diseases, diabetes, or autoimmune disorders) will not be eligible for our trial.

3.3.2. Exclusion Criteria

The following criteria will be checked at the time of enrollment. If any apply, the participant will not be included in the study.

1. History of chronic condition(s) requiring treatment such as cancer, chronic hepatic or kidney diseases, diabetes, or autoimmune disorders.
2. Acute or chronic, clinically significant pulmonary, cardiovascular, neurologic, hepatic or renal functional abnormality, as determined by physical examination or previous laboratory tests carried out by the participant's doctor.
3. History of allergic reaction (e.g., difficulty breathing) after receipt of any vaccine.
4. History of vaccination against hepatitis A (the control vaccine is a hepatitis A vaccine) or a known history of hepatitis A infection.
5. History of chronic administration (>14 days) of immunosuppressants or immune-modulating drugs within 6 months prior to the first vaccine dose. (For corticosteroids, this will mean prednisone, or equivalent, ≥0.5 mg/kg/day. Inhaled and topical steroids are allowed.)

6. Are sexually experienced and of childbearing potential (i.e., not surgically sterilized), and are unwilling to use an effective method of birth control for 30 days before vaccination until 60 days after the last vaccination (approximately 9 months)
7. Uterus has been removed.
8. Use of any investigational or non-registered product (drug or vaccine) other than the study vaccines within 30 days preceding the first dose of study vaccine, or planned use during the study period.
9. Previous administration of monophosphoryl lipid A (MPL® [Monophosphoryl lipid A; Manufactured by Corixa Corporation, Seattle, Washington, USA (Formerly Ribi)]) or AS04 adjuvant (no vaccines currently licensed contain these).
10. Previous vaccination against human papillomavirus.
11. History of allergic disease, suspected allergy or reactions likely to be exacerbated by a component of the study vaccines, i.e., 2-phenoxyethanol or neomycin.
12. Hypersensitivity to latex (found in the tip cap and syringe plunger).
13. Any medically diagnosed or suspected immunodeficient condition based on medical history and physical examination (no lab testing required).

Those women who report having a history of chronic conditions requiring treatment, an acute or chronic functional abnormality noted upon physical examination, an allergic response to any previous vaccination or to components of the vaccine/syringe, medically diagnosed or suspected immunodeficient conditions (note that this assessment is based on medical history and physical examination; no lab testing is required), or a recent history of chronic use of immunosuppressant or immune-modulating drugs will also be excluded, to assure safety of the participants. Women with a history of hepatitis A vaccination or of acute hepatitis A disease will also be excluded, since HAV will be used as the control vaccine in our trial and revaccination is not currently indicated in these groups.

Although there is no indication to date that the HPV16/18 VLP vaccine might adversely affect pregnant women or their unborn children, the possible risks to pregnant women and unborn babies are not known. Therefore, we intend to exclude from the trial sexually experienced women who do not agree to use an effective form of birth control from 30 days before vaccination until 60 days after the last vaccination (approximately 9 months total). Acceptable forms of birth control include abstinence; surgical sterilization; oral contraceptives; the intrauterine contraceptive device; a diaphragm or condom; or injectable contraceptives including implants, monthly injectables, and three-monthly injectables.

Women without an intact uterus will be excluded from the trial since they are not at risk of developing cervical HPV infection or CIN2+.

Finally, women who report recent (within 30 days) or planned (during the study period) use of an investigational or non-registered product (drug or vaccine, including *MPL*, AS04, and HPV vaccine) will be excluded. This will be done to avoid having an adverse event occurring in response to one of these other agents being confused with an adverse event resultant from study vaccination.

3.3.3. Deferral Criteria

Women will have their enrollment into the trial and/or vaccination visits (Amended: 31 Oct 2005) deferred if they:

- Received immunoglobulins within 90 days preceding enrollment/vaccination visit (Amended: 31 Oct 2005).
- Received a registered vaccine within 30 days of enrollment/vaccination visit. Administration of routine Meningococcal, Hepatitis B, Influenza, and Diphtheria/Tetanus vaccines up to 8 days before any dose of study vaccine is allowed. (Amended: 31 Oct 2005)
- Are suffering from an acute disease or have an oral temperature ≥37.5°C at enrollment/vaccination visit (Amended: 31 Oct 2005). Acute disease is defined as the presence of a moderate or severe illness with or without fever. Vaccines can be administered to women with a minor illness such as diarrhea and mild upper respiratory infection.
- Are pregnant, less than three months post-partum, or breastfeeding at the time of enrollment/vaccination visit. If the participant reports a menstrual delay and her pregnancy test is negative, she will be deferred for a minimum of seven days. Women with a second negative pregnancy test after the minimal deferral period would be eligible for enrollment/vaccination. A menstrual delay is defined as a delay of more than five days for women with regular menses and more than 35 days for women with irregular menses. (Amended: 31 Oct 2005)
- Are sexually experienced and using an effective method of birth control (including abstinence) for less than 30 days at the time of enrollment/vaccination visit. (Amended: 31 Oct 2005)
- Are sexually experienced but cannot have a pelvic exam performed due to heavy bleeding (menstruation or other) or heavy vaginal discharge. This criteria applies only to sexually experienced women at enrollment or those who require a pelvic exam at the Month 6 visit. (Amended: 31 Oct 2005)

Women who have received immunoglobulins (within 90 days of enrollment) and/or other vaccinations (within 30 days of enrollment) or who have current acute illnesses will be deferred to avoid confounding of our findings by these other vaccinations/conditions (for example, an adverse event to another vaccination).

Although there is no reason to believe that HPV16/18 VLP vaccination would adversely affect pregnant women or their unborn child, the possible risks to pregnant women and unborn babies are not known. Therefore, we have chosen to defer enrollment of pregnant women. A sensitive pregnancy test will be performed at the study clinic prior to each vaccination, and women found to be pregnant will have their enrollment deferred until they are at least three months post-partum and no longer breastfeeding. It should be noted that women who are found to be pregnant at the time of their one or six month vaccinations, despite use of an acceptable form of birth control, will not receive their subsequent vaccinations and will have their follow-up pelvic examinations and other follow-up activities deferred until they are at least three months post-partum.

Sexually experienced women who are not pregnant but report not using an acceptable form of birth control (including abstinence; surgical sterilization; oral contraceptives; the intrauterine contraceptive device; a diaphragm or condom; or injectable contraceptives including implants, monthly injectables, and three-monthly injectables) for at least 30 days will be deferred until they begin practicing birth control.

Women will also be deferred if, for any reason, a pelvic examination cannot be performed at the time of the initial visit. A pelvic examination will allow us to determine whether a woman has prevalent HPV infection and/or SIL at the time of initial vaccination. Although initial cytological and virological status will not be used to define a woman's eligibility for participation in the trial, it is important that cytological and HPV status at enrollment be obtained for sexually experienced women since the vaccine may not protect against an already established infection/lesion. It can be assumed that virginal women are unexposed to HPV and not at risk of SIL. The low rates of exposure to HPV among virginal women in Guanacaste have been confirmed by serological testing of self-reported virginal women in our natural history study.

3.3.4. Elimination criteria from the ATP analyses during the study

For the primary according to protocol (ATP) analysis planned for our study, the following elimination criteria may apply. These are also discussed in the statistical analysis section of this protocol (Section 4.2).

- Women who do not receive all three doses of the vaccine within the protocol-specified intervals (see Table 4).
- Women who are found to be HPV16 and HPV18 DNA positive ***(for the corresponding type considered in the analysis)*** during the vaccination phase***, by clinician and/or self-administered collection*** (months 0 or 6) ***(Amended: 10 September 2009)***.
- Women who indicate chronic administration (defined as more than 14 days) of immunosuppressants or other immune-modifying drugs occurring in the 90 days prior to blood sampling. For corticosteroids, this will mean prednisone, or equivalent, $\geq$0.5 mg/kg/day. Inhaled and topical steroids are allowed. It shall be noted that concomitant medications administered during this study will be collected only in the context of assessing SAEs. In specific, we will collect information on medications that are taken in the month preceding an SAE and those that are taken to treat an SAE if, in the investigator's judgement, the medication used to treat the SAE can help clarify the diagnosis of the SAE or if the medication is an immunosuppressant.

3.4. Contraindications to Subsequent Vaccination

The following events constitute a contraindication to further vaccination. If any of the events listed below occur, women will not receive further doses of the vaccine but will continue to be followed for the full four years or until resolution of the AE, whichever occurs last. The decision to discontinue vaccination will be carefully documented on the appropriate CRF, along with the date and reason for vaccine discontinuation. Women

who fail to receive one or more doses of vaccine will not be included in the ATP analysis but will be included in the ITT analysis (see Section 5).

Hypersensitivity reaction following vaccine administration

- Pregnancy
- Any medically diagnosed or suspected immunodeficient condition based on medical history or physical examination (no lab testing required)
- Any other acute or newly acquired chronic condition at the time of scheduled vaccination as determined by the medical history, which in the opinion of the study doctor precludes further administration of the study vaccine
- Any SAE judged to be possibly, probably, or definitely related to study vaccine
- Other significant reactions including severe pain, severe swelling, severe limitation of motion, persistent high fever, severe headache or other systemic or local reactions, which in the opinion of the study doctor preclude further administration of the study vaccine

3.5. Trial Arms & Randomization

The present trial will be a double blinded, randomized trial consisting of two arms. Women randomly assigned to the experimental arm will receive the HPV16/18 VLP vaccine while those randomly assigned to the control arm will receive the HAV vaccine, as described more fully in Section 3.6, Section 3.7, and Section 3.10. Women who agree to visit one of the study clinics and who fulfill the eligibility criteria described earlier will be randomly assigned to one of the two trial arms at an overall ratio of 1:1. Because the HPV16/18 VLP and HAV vaccines are indistinguishable in their appearance, we are able to and will mask investigators and participants to trial arm assignment.

A blocked randomization procedure will be used, with random block sizes used to avoid the possibility of unmasking at the clinic site. Details of the vaccine labeling and subject randomization process are provided in Appendix B.

Since knowledge of vaccine arm assignment would not affect treatment of participants who have an SAE, no provision is made to allow for unblinding on site in Costa Rica. Unblinding of individual participants, at the request of the DSMB, the IRBs, the GSK Global Clinical Safety Department or the NCI Medical Monitor as part of reporting requirements to the FDA (e.g., for rapid reporting of an unexpected SAE associated with vaccination) will be allowed. This individual unblinding will be performed in a manner that assures that the overall study blinding is maintained, that staff involved in the conduct, analysis, or reporting of the study remain blinded except where absolutely impractical, and that any unblinding be appropriately documented ***(see also Section 3.16.3, Exiting Women from the Trial) (Amended: 10 September 2009)***. Treatment codes will be kept at NCI and GSK under controlled/secured access.

3.6. Study Vaccines

The candidate HPV16/18 VLP-based vaccine to be used in our trial has been manufactured by GSK Biologicals. The control vaccine (investigational formulation of *Havrix* hepatitis A vaccine - HAV) has been developed and manufactured by GSK Biologicals. The Quality Control Standards and Requirements for the candidate vaccine and the control vaccine are described in separate release protocols and the required review and agreement of the FDA will be obtained before initiation of the trial.

The candidate HPV16/18 virus-like particle (VLP)/AS04 vaccine will be supplied as a liquid in mono-dose syringes. HPV16/18 VLPs at a concentration of 40 μg/0.5 mL are formulated with AS04 adjuvant. AS04 is an investigational adjuvant comprised of 500 μg of aluminum hydroxide ($Al(OH)_3$) and 50 μg of monophosphoryl lipid A (*MPL*). The control HAV vaccine will be supplied as a liquid in mono-dose syringes and will have an indistinguishable appearance to the HPV16/18 VLP vaccine. Inactivated hepatitis A viral antigen at a concentration of 720 ELISA units/0.5 mL is formulated with 500 μg aluminum salts (as hydroxide: $AL(OH)_3$), 0.5% 2-phenoxyethanol (w/v), and contains trace amounts of neomycin sulfate (no more than 20 ng per 0.5 mL dose).

Three lots of HPV16/18 VLP vaccine have been prepared to support the Phase III efficacy trial. Each HPV16/18 VLP vaccine lot is manufactured using one single batch of HPV16 L1 VLP antigen and one single batch of HPV18 L1 VLP antigen. Four lots of an investigational formulation of *Havrix* control vaccine have been prepared to support the Phase III efficacy trial. Formulation of the investigational *Havrix* control vaccine lots was performed using one batch of purified inactivated Hepatitis A antigen.

It should be noted that after completion of this trial and under a separate protocol, participants will be offered cross-over vaccination. This will include offering the licensed formulation of *Havrix* (1440 ELISA units/0.5 mL) hepatitis A vaccine to women who received the HPV16/18 VLP vaccine and the ***licensed*** HPV16/18 VLP vaccine to women who received the investigational hepatitis A vaccine ***(Amended: 10 September 2009)***. In addition, all women will be offered vaccination with Engerix-B® (Hepatitis B Vaccine [Recombinant]; Manufactured by GlaxoSmithKline Biologicals, Rixensart, Belgium). ***In order to reduce clinic visits, women who are eligible to recieve both the hepatitis A vaccine and hepatitis B vaccine will be offered vaccination with Twinrix® [Hepatitis A Inactivated & Hepatitis B (Recombinant) Vaccine] (Amended: 10 September 2009).***

The Material Safety Data Sheets (MSDSs) for HPV16/18 VLP vaccine and the investigational formulation *Havrix* that will be administered during this study are provided as Appendix E of this protocol.

At the time of study start, five Phase I-IIb clinical trials were conducted that evaluated vaccine safety.(Amended: 13 Nov 2008). During these trials, the 40 μg HPV16/18 VLP AS04 vaccine (i.e., the formulation planned for use in the current trial) was administered to approximately 690 young women living in Brazil, Canada or the United States who ranged in age from 15 to 30 years. No Serious Adverse Events associated with this formulation were considered by the investigators to be related to vaccination.

The single largest study evaluating HPV16/18 VLP vaccine safety was GSK study HPV-001. In this double-blind randomized trial, 1113 subjects (560 vaccine, 553 placebo) were evaluated. Subjects who received vaccine reported slightly higher rates of solicited local adverse events (injection site pain, redness, and swelling) compared with the placebo group; however, the rates of solicited general adverse events were similar between the vaccine and placebo groups. Overall, most solicited local and solicited general adverse events were low-grade in intensity and transient. Rates of unsolicited symptoms with onset within 30 days after each vaccination were similar in both groups.

The results of these clinical trials suggest that the HPV16/18 VLP vaccine is safe and well tolerated. However, with the administration of any vaccine there may be unanticipated allergic reactions (which may include rash and/or other allergic manifestations) and, as with the administration of any foreign protein, there may be rare occurrences of anaphylactic reaction. Please refer to the Investigator Brochure (IB) for a review of the HPV16/18 VLP vaccine pre-clinical and clinical studies. (Amended: 13 Nov 2008)

3.7. Selection of an Investigational Formulation of *Havrix* as the Control Vaccine

The purpose of an active control is to confer benefit to recipients while maintaining the study blind. Therefore, the control vaccine must match the HPV16/18 VLP candidate vaccine in appearance, reactogenicity, injection volume, and number of doses. Moreover, the control vaccine must be manufactured by GSK Biologicals to enable labeling (for blinding and randomization). Another important consideration is limited prior use of the active control vaccine in the population targeted for enrollment, since individuals previously vaccinated with the active control vaccine would be ineligible for our study. In addition, Costa Rica, and particularly Guanacaste, is considered an intermediate to high incidence area for hepatitis A and a low risk area for hepatitis B [Taylor, 2001].

Based on the above criteria, and in consultation with the trial DSMB, we have selected an investigational formulation of Hepatitis A vaccine (HAV) called *Havrix* as the control vaccine for our trial. A 3-dose, 720 ELISA unit (EL.U.) formulation of *Havrix* is registered for use in Costa Rica, although it has been supplanted by a more convenient 2-dose 1440 EL.U. formulation in recent years. All marketed *Havrix* products have a 1.0 mL injection volume. To maintain the blind, lots of *Havrix* 720 EL.U. investigational formulation will be prepared with a 0.5 mL injection volume; they will be labelled "For Investigational Use Only". The safety, immunogenicity, and efficacy of the *Havrix* investigational formulation is expected to match that of the *Havrix* 720 EL.U. formulation presently registered in Costa Rica.

In the United States, *Havrix* is licensed for use in children and adults. In adults, the vaccine is administered as a single dose of 1440 ELISA units (EL.U.) adsorbed on 0.5 mg Alum in 1.0 mL, followed by a booster dose 6 to 12 months after the primary dose.

There is a sufficient body of data to support that a 720 EL.U. dose of *Havrix*, administered to adults on a 0, 1, 6 month schedule, will be safe and immunogenic. This data is summarized below.

First, *Havrix* 720 EL.U./0.5 mg Alum in 1.0 mL has been licensed for use in adults in several countries on a 0, 1, 6 month schedule. Between 1992 and 1997, *Havrix* 720 EL.U. adsorbed on 0.5 mg Alum (1.0 mL) was registered for use on a 0, 1, 6 month schedule in 68 countries including Canada, Australia, the United Kingdom, Brazil and Sweden. Data from trials which included 1,025 adults who were given *Havrix* 720 EL.U./0.5 mg Alum (1.0 mL) on a 0, 1, 6 month schedule were also included in the original product license application (PLA) (Ref. No. 92-0465) submitted for *Havrix* and supported licensure in the US. In most of the countries where *Havrix* 720 EL.U. (1.0 mL) was registered for use in adults on a 0, 1, 6 month schedule, this formulation has now been replaced by a formulation containing 1440 EL.U. of viral antigen adsorbed on 0.5 mg Alum per 1.0 mL dose, since the higher antigen content formulation can be given on a 2-dose schedule.

Second, *Havrix* 720 EL.U./0.25 mg Alum in 0.5 mL (pediatric formulation) is currently licensed in the US for use in children aged 12 months (Amended: 31 Oct 2005) to 18 years on a 0, 6-12 month schedule. The 720 EL.U. dose has been demonstrated to be safe for use in children in numerous clinical trials and through post-marketing surveillance. This suggests that the formulation can be safely administered to adults.

Finally, the formulation of Twinrix® (Hepatitis A Inactivated & Hepatitis B [Recombinant] Vaccine; Manufactured by GlaxoSmithKline Biologicals, Rixensart, Belgium) currently licensed in the US for use in adults contains 720 EL.U. of hepatitis A virus antigen (and 20 μg HBsAg adsorbed on 0.45 mg Alum) per 1.0 mL and is administered on a 0, 1, 6 month schedule. Data from 11 clinical trials involving a total of 1812 subjects submitted as part of the *Twinrix* PLA (BB-IND 6986) and post-marketing surveillance data indicate that this dose and schedule of hepatitis A virus antigen is both safe and immunogenic in adults. It is reasonable to expect that the same dose and schedule of *Havrix* (720 EL.U. given on a 0, 1, 6 month schedule) administered as a monovalent vaccine will be safe and immunogenic in adults.

In summary, there is a preponderance of evidence to suggest that investigational *Havrix* 720 EL.U. in 0.5 mL will be safe and immunogenic when administered to adults. This makes it a suitable active control for this HPV vaccine efficacy study. The use of investigational *Havrix* as a control will allow this study to be conducted in a fully blinded fashion and will offer benefit for the women allocated to the control group.

3.8. Central Storage of Vaccine

Vaccine syringes will be shipped from GSK Biologicals in Rixensart to the study site in Costa Rica at 4°C (range 2°C to 8°C). Once in Costa Rica, the vaccine shipment QC procedures detailed in the Field Procedures Manual will be followed to ensure that the vaccine has been maintained at the proper temperature during shipment. At the Costa Rican repository, boxes containing vaccine syringes will be transferred and stored in a walk-in refrigerator located at the central study repository and kept at 4°C (range 2°C to 8°C) and will never be frozen. The walk-in refrigerator will be equipped with temperature monitors, with a back-up refrigeration unit and generator, and will be monitored on a daily basis by trained staff. Access to the vaccine storage facility will be restricted to authorized personnel only.

Deviations in temperature beyond those allowable by protocol that occur either during vaccine storage, vaccine transport or vaccine use will be documented and the NCI principal investigator or his designee will be notified. NCI will also notify GSK Biologicals regarding such violations. Vaccines affected by such deviations will not be used for the trial without joint approval by NCI and GSK Biologicals.

3.9. Concomitant Medications/Vaccination

We plan to provide women in our rural population with two 500 mg acetaminophen pills after each vaccination with an indication to take the medication in case of a mild fever or pain. The use of this acetaminophen during the trial will be followed in the 10% random subset of women for whom a home visit is performed on Day 3 to Day 6 following each vaccination. Also, concomitant medications administered during this study will be collected in the context of assessing AEs. In specific, we will collect information on medications/vaccinations that are taken in the month preceding an AE and those that are taken to treat an AE if it is felt that the medication used to treat the AE can help clarify the diagnosis of the AE or if the medication is an immunosuppressant. (Amended: 29 Mar 2005)

3.10. Vaccination Route and Schedule

Participants will be vaccinated intramuscularly in the deltoid muscle (upper arm) and will receive three doses at 0, 1, and 6 months, as summarized in Table 3 below.

Table 3 Vaccine Dosage and Administration

Timing	Dose	Vaccine/Control	Route	Site	Side
Month 0	1	√	Intramuscular	Deltoid	Non-dominant
Month 1	2	√	Intramuscular	Deltoid	Non-dominant
Month 6	3	√	Intramuscular	Deltoid	Non-dominant

3.11. Clinic Visits During Vaccination Phase (0, 1, 6, & 7 months)

Three clinic visits are planned for vaccination at 0, 1, and 6 months. At the first clinic visit, we will collect information for determining eligibility, ensure that written informed consent has been obtained prior to any study procedure, perform urine pregnancy testing, assign treatment arm by randomization, give the initial vaccination, and monitor women for at least 60 (Amended: 31 Oct 2005) minutes following vaccination with appropriate medical treatment readily available in case of a rare anaphylactic reaction. At the two subsequent visits at 1 and 6 months, information on AEs will be collected, subsequent vaccinations will be administered, and women will be monitored for a minimum of 60 (Amended: 31 Oct 2005) minutes following vaccination with appropriate medical treatment readily available in case of a rare anaphylactic reaction. After each vaccination visit, women will be provided with two 500 mg acetaminophen pills with an indication to take the medication in case of a mild fever or pain. The use of acetaminophen during the trial will be followed in the 10% home visit subset. A summary of the procedures performed at each of these visits is presented in Table 2. Intervals between study visits during the vaccination phase of the trial are provided in Table 4.

Blood specimens will be collected at each of these three timepoints to permit evaluation of antibody titers at the time of each vaccination and to allow for research-based testing. Blood collected at entry will also be used to measure hematocrit levels, as a service to participants. Cervical secretions and cervicovaginal cells will be collected from non-virginal women at entry and at the six-month visit, to assess HPV status at the beginning and end of the vaccination schedule and to allow for research tests. Cervicovaginal cells collected at the first pelvic examination will also be used for *Chlamydia trachomatis* and *Neisseria gonorrhoeae* testing using the Hybrid Capture test (Digene Corp., Gaithersburg, MD, USA). A ThinPrep® (a standardized cervical specimen preparation method; Manufactured by Cytyc Corporation, Boxborough, MA, USA) Pap smear will also be prepared (non-virginal women only) before the first vaccination, to assess cytological status at entry. An interview will be conducted at entry and at the six-month visit to assess exposure to known and suspected risk factors for HPV infection and cervical cancer.

To permit characterization of maximal antibody titers achieved after all three doses of vaccine are administered, a blood draw is also planned a minimum of 30 days after the third vaccination (7 month visit) on a subset of (Amended: 19 May 2006) 600 women randomly assigned to one of the two arms of our trial.

Table 4 Intervals Between Study Visits (Amended: 13 Nov 2008)

a. Yearly Visit Schedule

Interval	Length of interval (days)
1 (Month 0→Month 1)	21 to 62
2 (Month 0→Month 6)	161 to 216
3 (Month 6→Month 7)*	30 to 60**
4 (Month 0→Month 12)	301 to 660
5 (Month 0→Month 24)	661 to 1020
6 (Month 0→Month 36)	1021 to 1380
7 (Month 0→Month 48)	1381 to 1740#

b. Six-Monthly Visit Schedule

Interval***	Length of interval (days)
1 (Month 0→Month 1)	21 to 62
2 (Month 0→Month 6)	161 to 216
3 (Month 6→Month 7)*	30 to 60**
4 (Month 0→Month 12)	301 to 480
5 (Month 0→Month 18)	481 to 660
6 (Month 0→Month 24)	661 to 840
7 (Month 0→Month 30)	841 to 1020
8 (Month 0→Month 36)	1021 to 1200
9 (Month 0→Month 42)	1201 to 1380
10 (Month 0→Month 48)	1381 to 1740#

* This interval applies only to the 600 women enrolled in the immunogenicity subcohort (Amended: 19 May 2006), i.e., the women in the immunogenicity subset.

**Date of the Month 6 visit serves as the reference date.

***This schedule is to be followed by women detected as having evidence of LSIL (regardless of HPV testing results) or ASC-US concomitant with detection of an oncogenic HPV type.

N.B. Except for Month 7 where the date of the Month 6 visit serves as the reference date, the date of the first

vaccination serves as the reference date for intervals between study visits. Also, for Intervals 1 and 2, the length of interval listed represents the desired interval. Broader, allowable intervals are defined in Table 2. (Amended: 31 Oct 2005)

#Every effort will be made to schedule study participants within the preferred Month 48 study visit window (1381 to 1740 days post Month 0). However, the window for the last visit might be extended until October 3, 2010, which is the scheduled date of last subject last visit (i.e, 1740 days after the 4 year anniversary date for the last subject first visit).

3.12. Active Follow-up of Participants (Months 12-48)

Long-term follow-up is planned for a period of four years from the time of entry into the trial. Follow-up will be performed to monitor trial outcomes, to collect additional information on long-term AEs, and as part of NCI-sponsored ancillary studies to evaluate issues of immunological and etiological interest. A summary of procedures performed during follow-up is presented in Table 2. Intervals between study visits during the follow-up phase of the trial are provided in Table 4.

Follow-up will consist of yearly clinic visits, at which time the following activities will be completed:

- An interview to collect information on risk factors known or suspected to be associated with HPV infection, SIL, or the immune response to vaccination.
- An interview to obtain information on AEs (Amended: 29 Mar 2005), including hospitalizations, incapacitation lasting more than one day, pregnancies, and other events reported by participants. AEs and SAEs are further defined in Appendix D.
- A pelvic examination (if the participant reports previous sexual activity) to collect cervical specimens for cytological and virological evaluation. Cervical secretions will also be collected at this time for ancillary evaluation of local immune responses.
- A blood specimen will be collected for HPV antibody and other testing. On an approximately (Amended: 19 May 2006) 10% subset of women at enrollment and at the 7 (Amended: 19 May 2006), 12 and 36 month visits, additional blood for ancillary studies will be collected to permit cryopreservation of lymphocytes for immunological assessment.

Women who at any of the yearly screening visits are found to have evidence of ASC-US+/LSIL will be seen at six-month intervals (rather than yearly intervals), and will continue to be seen every six months until there is evidence of disease persistence/progression (in which case they will be referred for colposcopic evaluation) or three consecutive normal cytologies are observed (in which case they will return to a yearly screening interval). Women who at any of the clinic visits are found to have evidence or suspicion of AGC/ASC-H/HSIL/cancer will be immediately referred for colposcopic evaluation and excisional treatment, if needed (Amended: 13 Nov 2008). Participants referred for colposcopic evaluation will be seen by an expert study colposcopist. Detailed colposcopy referral and colposcopy management algorithms are provided in Appendix A. Women who are referred for colposcopic evaluation, whether treated or not, will continue to be actively followed at six month intervals using the criteria defined above for women diagnosed with ASC-US+/LSIL.

Women who are determined to be pregnant at the time of their scheduled follow-up visit will be deferred until they are three months post-partum. Please note that pregnancy outcomes will be documented for all pregnancies among trial participants, via participant report and review of medical charts.

A small proportion of cytology and HPV specimens is expected to be inadequate and/or unsatisfactory for evaluation (<5%). In such cases, we do not intend to resample the women until her next scheduled clinic visit. However, to ensure optimal clinical management of women with inadequate/unsatisfactory results, we will presume an LSIL cytology and HPV DNA positivity. This will effectively mean that women with inadequate/unsatisfactory results are either rescreened at 6-month intervals (if no previous history of ASC-US+/LSIL), or referred for colposcopic evaluation (if previous history of ASC-US+/LSIL).

3.13. Trial Endpoints

3.13.1. Primary Endpoint

The primary endpoint for our trial will be histopathologically confirmed, incident CIN2+ (defined as CIN2, CIN3, AIS, or invasive cervical cancer) associated with an HPV16 or HPV18 infection. To define histopathological endpoints, a panel of expert pathologists will review all histopathological material collected during the course of our trial. This expert panel review will supercede the initial local histopathological diagnosis for purposes of defining the primary endpoint for US FDA submission. Panel members will review histopathological material blinded to trial arm assignment and to HPV DNA status or other participant characteristics. They will also review specimens blinded to the interpretation given by other panel members. Should the initial independent assessment differ between panel members, adjudication will be performed by consensus. Additional details of the plan for histology review to define study outcomes are provided in Section 3.19 (Laboratory Tests). Women diagnosed with cytological evidence of HSIL but without histopathological confirmation of CIN2+ will not be considered for analysis of the primary endpoint.

HPV16 and HPV18 status will be determined by testing cervical cells collected into PreservCyt® (Cell preservative solution; Manufactured by Cytyc Corporation, Boxborough, MA, USA) medium using PCR-based general primers (SPF_{10}) followed by LiPA and HPV16 and HPV18 type-specific PCR-based primer systems. The algorithm for PCR-based testing is provided as Appendix C. Additional details of the plan for HPV testing are provided in Section 3.19 (Laboratory Tests). For women who test positive for multiple HPV types, including HPV16 or HPV18, we will assume that HPV16 or HPV18 are associated with the CIN2+ lesions (i.e., will count as an outcome).

Formatted: English (U.S.)

3.13.2. Secondary Endpoints

In addition to histopathologically confirmed CIN2+ and HPV16 and HPV18 infection, which are described above, other endpoints required to achieve the six secondary objectives of our trial are discussed below.

To evaluate the safety of our vaccine (Secondary Objective #1), we plan to examine:

1. Occurrence and intensity of solicited local adverse events and occurrence (either onset or aggravation) of solicited general adverse events including urticaria within 60 (Amended: 31 Oct 2005) minutes after each vaccination and over all vaccinations combined.
2. Occurrence and intensity of solicited local adverse events and solicited general adverse events in a 10% random subset of participants on one day from Day 3 to Day 6 after each vaccination and over all vaccinations combined.
3. Occurrence of unsolicited AEs and SAEs throughout the entire study (Month 0 up to Month 48).
4. Outcome of all pregnancies.

To evaluate the duration of protection conferred by the candidate vaccine (Secondary Objective #2), we will examine the time to occurrence of incident cervical infection with HPV16 or HPV18 (by PCR). HPV16 and HPV18 infection will be defined as described in Section 3.19 of this protocol (Laboratory Tests).

To evaluate the efficacy of the candidate vaccine to protect against infection with any oncogenic HPV type (Secondary Objective #3), we will use as an endpoint histopathologically confirmed CIN2+ associated with infection by any oncogenic HPV type detected by PCR in the preceding cervical cytology specimen. Whenever oncogenic and non-oncogenic HPV types are detected in a woman diagnosed with CIN2+, we will assume that the lesion was caused by one of the oncogenic types detected. Additional details of the plans for histological assessment and HPV testing are provided in Section 3.19 (Laboratory Tests).

To more strictly evaluate whether the candidate vaccine protects against incident HPV16 or HPV18 associated CIN2+ among unexposed individuals (Secondary Objective #4), we will evaluate vaccine efficacy against the primary study endpoint while further restricting the evaluation to women who are HPV DNA (by PCR) negative at months 0 and 6 for the corresponding HPV type, seronegative by ELISA at month 0 for the corresponding HPV type, and who have evidence of HPV16 or HPV18 infection detected within the lesional component of the cervical tissue specimen by PCR. Details of the plans for HPV DNA and serologic testing are provided in Section 3.19 (Laboratory Tests).

To evaluate the efficacy of the candidate vaccine to protect against persistent infection with HPV16 or HPV18 (Secondary Objective #5), we will define as persistent infection any HPV16 or HPV18 infection that persists (same type persistence) for approximately 12 months or longer. The word “approximately” is used here because our annual visits can occur slightly less (or more) than 12 months apart based on our protocol-defined visit intervals (see Table 4). For women who are being followed at 6-month intervals, the intervening HPV result collected at the 6-monthly visit will also be required to be positive for the same HPV type for a woman to be defined as having a persistent HPV infection. Details of the plans for HPV testing are provided in Section 3.19 (Laboratory Tests).

Finally, to confirm that immunogenicity in our population is comparable to that seen in previous Phase I/II trials and to define long-term immunogenicity of the candidate vaccine (Secondary Objective #6), we plan to measure HPV16 and HPV18 antibody levels by ELISA and V5/J4 monoclonal antibody inhibition EIA on blood specimens collected from the 600 women recruited into the immunogenicity subcohort of (Amended: 19 May 2006) our trial. Details of the serology testing plan are provided in Section 3.19 (Laboratory Tests)

3.13.3. Tertiary and Exploratory Endpoints (Amended: 13 Nov 2008)

In addition to the primary and secondary endpoints listed above, NCI and Costa Rican investigators plan to evaluate numerous additional exploratory virological and immunological endpoints as surrogate endpoints in our trial. A full description of these endpoints is beyond the scope of this protocol, but includes:

- Persistent HPV16 or HPV18 infection at intervals other than the 12-month interval defined for secondary objective #5 and defined based on intratypic variant testing.
- High viral load HPV16 or HPV18 infection (whether transient or persistent).
- HPV16 or HPV18 VLP antibodies detected in serum by ELISA, pseudovirion neutralization, or other methods that might be developed during the course of the trial.
- HPV16 or HPV18 VLP antibodies detected in cervical secretions by ELISA or other methods that might be developed during the course of the trial.
- T-cell proliferative assays for HPV16 or HPV18 VLP.
- T-cell cytokine production assays for HPV16 or HPV18 VLP.
- T-cell cytotoxicity assays for HPV16 or HPV18 VLP.

3.14. Adverse Events Monitoring

One of the major objectives of the present trial is to obtain additional information on the AEs associated with HPV16/18 VLP vaccination. While safety and immunogenicity trials in the United States and elsewhere have suggested the overall safety of the HPV16/18 VLP-based vaccine, rare AEs cannot be ruled out in these early phase studies given the relatively limited number of volunteers involved (approximately 690 individuals vaccinated at the time of study start with the GSK Biologicals candidate HPV16/18 VLP-based vaccine and approximately 2,200 individuals vaccinated at the time of study start with other VLP-based vaccines). (Amended: 13 Nov 2008) Thus, it is incumbent on those conducting large-scale efficacy trials to collect additional safety data. Criteria for defining AEs, SAEs, and examples of each are provided in Appendix D.

Our plan to monitor all trial participants for solicited and unsolicited AEs (whether expected or unexpected) is summarized below.

- **AEs occurring immediately following vaccination.** We will observe all vaccinees for at least 60 (Amended: 31 Oct 2005) minutes at the clinic following each

vaccination with appropriate medical treatment readily available in case of a rare anaphylactic reaction. This will be performed under the supervision of trained medical staff. During this 60 (Amended: 31 Oct 2005) minute observation period, we will collect information on vaccine reactogenicity, including both solicited and unsolicited events, as summarized on the Vaccine Administration and Reactogenicity Monitoring (VAR) and AER (Amended: 31 Oct 2005) forms that are provided separately from this protocol.

- **AEs occurring in the week following vaccination.** We plan to visit a random sample of 10% of vaccinees once during the week following each vaccination to collect information on solicited AEs, unsolicited AEs, and use of study-provided acetaminophen. This visit will be conducted by a specially trained outreach health worker between Days 3 and 6 after each vaccination. The information collected during this visit is summarized on the Home Visit Reactogenicity Monitoring and AER (Amended: 31 Oct 2005) forms that are provided separately from this protocol.
- **Long-term active monitoring.** We will schedule all participants for annual clinic visits for a period of 4 years from enrollment. At the time of each clinic visit, we will collect information on long-term, unsolicited AEs, and SAEs. The clinic visit will be performed by a trained study doctor or nurse and will include a pelvic examination for sexually experienced women. The Post-Enrollment Eligibility Screener and Adverse Event Report forms will be used to document all reported AEs. These forms are provided separately from this protocol.
- **Long-term passive monitoring.** We will provide trial participants with a toll-free number that can be used at any time during our trial visit to report AEs to our trained emergency response team. Participants will be instructed to call this toll-free number should they have any medical problems, whether they think the problem is related or not to vaccination. The phone will be staffed 24 hours a day by a physician who will triage all calls and refer as needed. The AER (Amended: 29 Mar 2005) form will be used to document all AEs reported through this mechanism. This form is (Amended: 29 Mar 2005) provided separately from this protocol.
- **Monitoring pregnancy outcomes.** Information regarding pregnancies and their outcomes will be obtained at every follow-up visit performed during the trial. For women who are lost to follow-up, information will be captured via linkage to hospital records. We plan to document outcomes for all pregnancies occurring among vaccinees during the course of our study. For deliveries, we will review and abstract relevant information from standardized forms that are completed for each delivery at all hospitals in Costa Rica. Review will be conducted by trained abstractors from our staff at regional hospitals in Guanacaste and hospitals outside of Guanacaste to which women from Guanacaste are sometimes referred. Alternatively, the same information can be obtained from the “baby card” provided by the hospital to the mothers of newborns. Miscarriages (whether spontaneous or voluntary) resulting in hospitalizations or emergency room visits will be monitored as described below. Miscarriages occurring outside of a hospital setting will be documented by self-report during follow-up visits.
- **Monitoring specific AEs of interest in offspring.** At the request of the Costa Rican IRB specific adverse events occurring in subject's offspring who were conceived

within one month prior to first vaccination to one year after last vaccination will be reported at the final visit (Month 48). This includes the following medical event categories in the offspring:

- Endocrinologic and metabolic conditions
- Autoimmune diseases
- Sensory impairment
- Intellectual impairment
- Death

The occurrence of these specific events in offspring will be included in the clinical database. A child case will be created and linked to the mother case. Medical judgment will be exercised in deciding whether any disorders/diseases reported in offspring are included in these specific categories and should be reported as appropriate. Of note congenital malformations in offspring, as per original protocol, are to be documented and reported as SAEs and included in the pregnancy outcome analysis. (Amended: 13 Nov 2008)

- **Monitoring of hospitalizations and emergency room visits.** Hospitalization listings from all hospitals and emergency rooms in our study area will be reviewed by trained study staff. Listings from selected hospitals outside of Guanacaste to which women from Guanacaste are referred will also be reviewed by our staff. This will allow us to identify women in our trial who are hospitalized during the study period and who might not have contacted us via the toll-free number listed above. Medical records from these women will be reviewed and abstracted to obtain detailed information about the condition that led to hospitalization and/or the emergency room visit. This source of information will be important for the small fraction of women (<10% based on 10 years of experience from our natural history study in 10,000 women in Guanacaste) that we expect to be lost to follow-up during our study.

All AEs reported on the AER form (Amended: 31 Oct 2005) will be followed through resolution by a staff physician. Non-serious AEs will be reviewed by a staff clinician before participant close-out; participants with ongoing conditions will be referred to the appropriate referral center if needed. (Amended: 13 Nov 2008) AEs will be evaluated for their intensity, seriousness, and relationship to vaccination. In addition, the nature of each event (including a diagnosis using the ICD-10 coding structure), date and time of onset (where appropriate), and outcome (including resolution date) will also be documented. As outlined in Appendix D, all SAEs, regardless of expectedness will be reported by one of the Costa Rican investigators to the NCI Medical Monitor (who will in turn report to the DSMB) and the GSK safety group, in a timely manner, in compliance with FDA regulations (21 CFR 312.32). Definitions of seriousness, relationship to vaccination, intensity, and the procedures for and timing of reporting AEs are also provided in Appendix D.

It should be noted that all medical staff will receive special training to respond to clinical emergencies. Emergency resuscitation equipment will also be available at all study clinics.

3.15. Clinical Procedures

3.15.1. Overview

All participants are expected to have at least seven visits over the course of our trial (see also Table 2). This will include:

- An initial enrollment visit, at which time potential participants will be screened for eligibility, will be randomly assigned to one of two arms, and will receive their first vaccination
- Two clinic visits for the administration of additional vaccine doses (one and six months following the enrollment visit)
- A clinic visit will be conducted at seven months on a subset of (Amended: 19 May 2006) 600 women enrolled to allow for immunogenicity assessment a minimum of 30 days following the last vaccination
- Four long-term follow-up clinic visits at yearly intervals starting 12 months after enrollment, and continuing through their four-year anniversary

In addition to the clinic visits listed above, women diagnosed with ASC-US+/LSIL at entry or during follow-up will be followed at six-month intervals and referred for colposcopic evaluation if the lesion persists for six months or longer. Also, women found to have prevalent or incident HSIL or cancer at any time during the study will be referred for colposcopic evaluation and excisional treatment, if necessary.

3.16. Enrollment Physical Examination

Women selected for the trial will be invited to visit one of the study clinics to participate in the trial. At the time of the initial clinic visit, medical history information will be obtained and a complete physical examination will be performed to determine the overall health status and eligibility for the study. The Enrollment General Physical Exam and Medical History form that will be used to record results from this examination is provided separately from this protocol.

Immediately preceding the physical examination, and before each vaccination (at 0, 1, and 6 months), all participants will be asked to collect urine specimens for a urine pregnancy test. Only women with a negative pregnancy test and who report use of an acceptable form of birth control for at least 30 days and a willingness to continue use of an acceptable form of birth control until 60 days after the last dose of vaccine is administered (i.e., for the first eight months of the trial) will be enrolled. The enrollment of women who are pregnant will be deferred until they are at least three months post-partum and no longer breastfeeding.

3.16.1. Pelvic Examinations

Pelvic examinations will be performed at enrollment and during follow-up visits, starting at 12 months (i.e., at 12, 24, 36, and 48 months). These examinations will be performed

to permit a visual examination of the external genitalia and cervix, to collect cells for cytology and HPV DNA testing (and for *C. trachomatis* and *N. gonorrhoeae* testing by Hybrid Capture at the first pelvic exam), and to collect cervical secretions for HPV antibody and other testing. Conditions identified during these procedures will be documented on the appropriate CRF and will result in treatment by study staff or referral to the Costa Rican National Social Security Administration Health System. No pelvic examinations will be performed on virginal women and these women will be assumed to be HPV and SIL negative, since virginal women are known to be at extremely low risk of HPV infection and SIL. The low rates of exposure to HPV among virginal women in Guanacaste have been confirmed by serological testing of self-reported virginal women in our natural history study. No pelvic examinations will be performed on pregnant women, as discussed previously. Pregnant women will have their enrollment and/or follow-up visits deferred until they are at least three months post-partum. During enrollment, women who are more than three months post-partum but still breastfeeding will also be deferred until they are no longer breastfeeding.

The enrollment pelvic examination will be performed to assess whether a woman has prevalent HPV infection and/or ASC-US/SIL at baseline. As discussed earlier, cytological findings at entry will not be used to determine eligibility for vaccination. However, women with advanced disease (defined as women with suspected or confirmed HSIL and/or cancer and those with lesions of glandular origin) will be referred for colposcopic examination and, if confirmed, the lesion will be treated. Women with evidence of advanced disease during the first six months of the trial will receive no additional vaccinations until after colposcopic assessment and treatment (if necessary), but will continue to be followed for the duration of the trial, as described in the next section. Also, the presence of HPV16 or HPV18 DNA, antibodies to HPV16 or HPV18, and/or ASC-US/LSIL at entry will not result in exclusion from the trial, but assessment of baseline status is important to permit planned primary and secondary analyses restricted to individuals uninfected with HPV16 or HPV18 at entry (see Section 4).

While a pelvic examination will not be performed on women at their six month visit (unless there is evidence of ASC-US+/LSIL at the enrollment visit, as discussed below), a self-administered cervicovaginal cell collection will be performed at this visit to collect cells for HPV DNA testing. This will allow for a separate evaluation of women who develop incident HPV infection before the full vaccination schedule is completed. This will be important to determine whether vaccination failures are more likely before all vaccine doses are administered and “maximal” immunological protection is achieved. The self-administered collection proposed for our trial has previously been compared against clinician-administered collections and the two types of collection were found to correlate very well (88.1% agreement; kappa = 0.73) [Gravitt, 2001]. To ensure that self-administered specimens collected during our trial are comparable to clinician-administered sampling for HPV DNA testing, we plan a re-evaluation of these two collection methods within the context of our trial. Both specimen types will be collected and tested for HPV DNA (by PCR) from the subset of women in our trial who require a pelvic examination at their six-month visit due to evidence of ASC-US+/LSIL at the enrollment visit.

Follow-up pelvic examinations conducted at 12, 24, 36, and 48 months will permit active surveillance of trial participants for the primary and secondary outcomes of our trial. As mentioned previously, additional pelvic examinations at six-month intervals will be scheduled for women with cytological evidence of ASC-US+/LSIL at any of the routine annual screening visits (Appendix A). Women whose ASC-US+/LSIL lesion persists for six months or longer will be referred for colposcopic evaluation and excisional treatment, if needed.

Cytological specimens obtained during pelvic examinations will be reviewed as described in the Laboratory Tests section of this protocol (Section 3.19).

3.16.2. Referral for Colposcopic Evaluation

To assure prompt treatment of precancerous lesions of trial participants, women with cytological evidence of ASC-H by cytology, HSIL/cancer by cytology, with evidence of a glandular lesion by cytology, or with visual abnormalities suggestive of cancer will be rapidly referred for colposcopic evaluation and excisional treatment, if needed (Appendix A). If all doses of the vaccine have not been completed at the time the diagnosis of one of these advanced conditions is made, the woman will not receive any additional vaccinations until after colposcopic evaluation and treatment (if needed) is completed. Women referred for colposcopic evaluation (regardless of treatment) will continue to be followed actively throughout the four-year trial period to assure adequate clinical management and to collect information on potential vaccine AEs. These women will be followed at six-month intervals until there is evidence of persistent or progressive disease, or they have three consecutive normal screening visits.

Women with cytological evidence of ASC-US+/LSIL at any point during the trial will be seen for repeat screening visits every six months (Appendix A). Should the cytological abnormality persist for two visits (whether consecutive or not), these women will be referred for colposcopic evaluation and excisional treatment, if needed. As was the case for women diagnosed with advanced disease (ASC-H/HSIL/glandular lesions/cancer), those who have persistent ASC-US+/LSIL and are referred for colposcopic evaluation will continue to be followed actively at six-month intervals until there is evidence of persistent or progressive disease, or they have three consecutive normal screening visits.

Biopsy and LEEP specimens obtained during the trial will be retrieved for histopathological review by the study pathologists in Costa Rica and the NCI, as described in the Laboratory Tests section (Section 3.19). These histopathological specimens will also be used for research purposes.

3.16.3. Exiting Women from the Trial (Amended: 13 Nov 2008)

At the Month 48 visit we will apply the same criteria applied throughout the trial to define women who require colposcopic evaluation. Specifically, all women with evidence of cancer, HSIL, glandular lesions, AGC, ASC-H, or persistent ASC-US+/LSIL will be referred to colposcopy. Close-out of individual study participants will only occur after colposcopy referral, for women who require such referral. See Appendix A for more details.

It should be noted, that after participant close-out, participants will be offered additional clinical care if needed. Case management after study close-out will be defined under a separate protocol.

Activities at study completion (Amended: 10 September 2009)

The Data and Safety Monitoring Board (DSMB) for our trial has recommended cross-over immunization of both treatment and control recipients. The participants who received the HPV vaccine will be offered vaccination against hepatitis A and hepatitis B and participants who received the control vaccine will be offered vaccination against HPV-16/18 and hepatitis B, as appropriate. Vaccines to be used during cross-over immunization are the formulations licensed for use in Costa Rica. Cross-over immunization will be implemented by NCI and Costa Rican investigators under a separate protocol they have developed.

Implementation of cross-over immunization will require unblinding. Unblinding information and cross-over will not be offered to participants until they have been exited (closed out) from the present study and their data for this study have been frozen in the study databases. Exiting of participants in the trial will occur in an ongoing/batched manner as participants complete their 4-year participation in our study and after ongoing SAEs or pregnancies are resolved, or on October 3, 2010 (10 months after the 4-year anniversary for the last woman enrolled), whichever comes first. This will ensure that study blind is maintained for individual participants until their data for the final analysis is frozen.

If cross-over immunization cannot be offered to a subject (e.g., because of medical reasons) or the subject refuses cross-over immunization, unblinding information will be provided after the participant has exited the current study and her data has been frozen. This will occur at the time of the first cross-over implementation visit, at which time participation/eligibility for cross-over immunization will be determined.

3.17. Other Procedures and Data Collection

3.17.1. Overview

In addition to the clinical examinations described above, the following data and specimen collection is planned:

- Interviews at enrollment and during follow-up to ascertain information regarding HPV exposure risk (i.e., sexual behavior) and other socio-demographic, behavioral and exogenous factors that are either believed to be associated with the development of SIL or thought to potentially modulate the immune response to vaccination.
- Blood specimens will be collected at every clinic visit for HPV antibody testing and testing for other SIL risk factors (e.g., other sexually transmitted diseases) and/or factors postulated to modulate immune response to vaccination (e.g., HLA typing and T-cell responses to HPV). Blood specimens collected at the enrollment visit will also be used to perform a hematocrit as a benefit to participants.

- Oral, anal and vulvar specimen collection at the 48-month visit is being added to allow for the evaluation of the effect of vaccination on oral, anal, and vulvar HPV infection rates. (Amended: 13 Nov 2008)

It should be noted that, for sexually experienced women, both the interview and blood collection will be deferred whenever a woman's pelvic examination is deferred (e.g., due to pregnancy).

3.17.2. Interview Questionnaires

A brief, 10-15 minute questionnaire will be administered to all participants at enrollment. This questionnaire will assess information on factors believed to be associated with either SIL or immune response to vaccination. More specifically, the questionnaire will obtain information on:

- Socio-demographic factors
- Sexual history
- Reproductive behavior
- Contraceptive practices
- Cigarette smoking
- Family history.

A copy of the study enrollment questionnaire is provided separately from this protocol.

During follow-up (starting at the six month visit), additional questionnaires will be administered to participants at each clinic visit to ascertain changes in the factors listed above. Should some of the specific ancillary studies listed above be approved, additional questions relating to the specific hypotheses being addressed in the ancillary studies may also be added. A copy of the study follow-up questionnaire is provided separately from this protocol.

3.17.3. Blood Collection

Blood specimens will also be collected from all participants at each of the clinic visits (see Table 2). The major purpose of these blood collections will be to monitor immune response to vaccination, both humoral (i.e., antibody) and cell-mediated (i.e., T-cells). Aliquots of plasma, serum, buffy coat, peripheral blood mononuclear cells (PBMCs) and red blood cells (RBCs) will also be saved for other research-based assays, including antibody tests against sexually transmitted diseases other than HPV, hormone assays, nutritional assays (particularly those nutrients believed to impact the immune response), host DNA testing (to evaluate host susceptibility factors associated with SIL and the immune response to vaccination, such as HLA), and others. The blood specimen collected at study entry will also be used to perform a hematocrit as a benefit to participants. Women with evidence of anemia will be provided appropriate initial treatment and referral to the Social Security system.

Three types of blood draws are planned. These include the collection of 3 mL of blood for hematocrit testing (done as a benefit to participants), 10-20 mL of blood that will be kept cool after collection and processed locally within a few hours of collection, and the collection of an additional 40 mL of blood on a subset of participants that will be kept at room temperature and shipped overnight to a specialized laboratory set up to cryopreserve viable lymphocytes. The 3 mL blood draw will be obtained at enrollment only. The 10-20 mL blood draw will be collected from all participants at all clinic visits (20 mL at the enrollment and exit visits and 10 mL at all other intervening visits) and will be used for HPV antibody, host DNA, hormonal, nutritional, and other research-based assays. The 40 mL blood draw will be collected to allow for cryopreservation of PBMCs required to perform functional immunological testing planned as part of efforts by NCI and Costa Rican investigators to monitor the immune response of trial participants. This third blood draw will be collected for ancillary research purposes on a subset of participants, including a random sample of 10% of participants at enrollment and at their 12 and 36 month visits (to document the distribution of the immune responses in our population before and after vaccination), women referred to accelerated screening visits at six-month intervals ***through March 31, 2009 (Amended: 10 September 2009)*** (this group would have the additional blood sample collected annually), women who are referred for colposcopic evaluation and require a biopsy or LEEP, and women enrolled in the immunogenicity subcohort of our trial after March 1, 2006. (Amended: 19 May 2006) While the primary focus of this larger blood draw will be on the cryopreservation of PBMCs for immunological monitoring, residual plasma will also be saved for use in possible future assays.

While the primary focus of this larger blood draw will be on the cryopreservation of PBMCs for immunological monitoring, residual plasma will also be saved for use in possible future assays.

To assure that participation and retention rates in our trial are not negatively affected by the blood draws, refusal to have blood collected will not result in exclusion from the trial.

3.17.4. Extra-Cervical Collection

Oral, anal and vulvar specimen collection at the 48-month visit is being added to allow for the evaluation of the effect of vaccination on oral, anal, and vulvar HPV infection rates. Prior to the pelvic exam, an oral specimen will be collected. During the pelvic examination, a vulvar and an anal specimen will be collected prior to cervical sampling. Each specimen will be collected according to a standard procedure and will be stored in separate tubes. (Amended: 13 Nov 2008)

3.18. Biospecimen Processing, Storage, & Shipment

Collection of numerous types of biological specimens is planned, as mentioned in previous Sections. The types of specimens we propose to collect and plans for processing, storage and shipment are summarized in this Section.

The individual types of specimens we propose to collect and the expected number of aliquots resultant from this collection and requiring storage/shipment are summarized in

Table 5 to allow NCI to plan for adequate repository space at its main repository in Maryland, USA. In brief, we propose to collect cervical cells for liquid-based cytology, additional cervical cells for HPV DNA, RNA and other testing, cervical secretions for immunological assessment, cytological material (slides) for evaluation, histopathological material (blocks and slides) for review and testing, and blood for various immunological and other research-based tests, and oral, anal and vulvar samples (Amended: 13 Nov 2008). We have had experience collecting all of these biological specimens in Costa Rica on several thousand women over more than seven years as part of our natural history study.

Several biological specimens we propose to collect require little or no processing and can be stored at air-conditioned room temperature until shipment to the USA. These include the cells for liquid-based cytology and cytopathological materials. Other specimens require little or no processing but need to be frozen on the day of collection. These include the cervical cells for HPV DNA and RNA testing, and cervical secretions, and oral, anal and vulvar samples (Amended: 13 Nov 2008).

Table 5 Types of Biological Specimens Proposed and Expected Storage Requirements (Amended: 13 Nov 2008)

Specimen Type	# per Woman	Size	Total #*
Cervical Cells (Liquid Cytology/HPV)	6	25 mL vial	45,000
Cervical Cells (Research)	6	10 mL vial	45,000
Cervical Secretions	5	10 mL vial	37,500
Blood	70	1-2 mL vials	525,000
Tissue Slides (Cytology/Pathology)	6.25	Slides	46,875
Tissue Blocks	.25	Blocks	1,875
Oral Samples	1-3	20 mL vial	7,500
Anal Samples	1	1 mL vial	7,500
Vulvar Samples	1	1 mL vial	7,500

* Assuming 7,500 participants (repository estimation purposes; does not take drop-outs into account)

Blood specimens will require more extensive processing. For all participants at enrollment and during follow-up, we propose to collect 10-20 mL of blood and to separate and store its various components (plasma, serum, buffy coat, and red blood cells). Processing of these specimens will be performed by trained personnel on site who are devoted to this task. They will use specific Standard Operating Procedures (SOPs) developed for our trial.

Finally, we propose to collect additional blood specimens on a random sample of participants to permit cryopreservation of lymphocytes for use in functional immunological and other testing. Cryopreservation of lymphocytes is technically more challenging than the separation of whole blood into its components, and so collaborative arrangements have been established with investigators at the University of Costa Rica in San Jose to have specimens from our trial cryopreserved. We will have specimens requiring cryopreservation transported daily by a study driver to a university lab devoted to the processing of our trial specimens. Trained personnel will utilize the separation and cryopreservation procedures established by Biotech Research Laboratories, and used

widely on other DCEG studies including our Costa Rican natural history study. Standardized SOPs have been developed for use by the processing laboratory.

Biological specimens collected during our trial will be temporarily stored in a repository located adjacent to the trial administrative offices. This repository is outfitted with air conditioning, a walk-in refrigerator, and various liquid nitrogen tanks, and is staffed by trained personnel devoted to maintaining the repository.

Shipment of biological specimens will be performed periodically to one of the NCI repositories in Maryland, USA and further distributed, as needed, to collaborating laboratories for testing. Materials that can be stored at room temperature will be shipped on a monthly basis by air. Shipments will be made by cargo plane, since some of the materials being shipped contain methanol and are considered flammable. Materials that need to be stored frozen will be shipped in liquid nitrogen tanks. Given the large volume of specimens to be shipped as part of our study, we plan to have frozen specimens shipped in large liquid nitrogen tanks that are specifically manufactured to allow for air transport. A specialized shipping and customs agent will be responsible for these shipments. The agent we propose to use has been responsible for the successful shipment of biological specimens collected during our natural history study. Hundreds of shipments have been made from Costa Rica since 1993, and to date only one shipment (in the early phase of our study) was lost in transit.

3.19. Laboratory Tests

3.19.1. Cytology

Cervical cytology will be performed using the *ThinPrep* PapTest™ (Cytyc Corporation, Boxborough, MA, USA). Cervical cells for *ThinPrep* cytology will be collected at each clinic visit where a pelvic exam is performed using a Cervex® brush (Rovers Medical Devices BV, Oss, The Netherlands) rinsed into a collection vial containing *PreservCyt* medium. Two 0.5 mL aliquots of the 20 mL in the *PreservCyt* vial will be withdrawn for PCR testing (see below), prior to preparation of *ThinPrep* slides using the *ThinPrep* 2000 Processor. *ThinPrep* slides will be prepared by qualified trial staff at the local cytology staining laboratory, will be screened sequentially by two cytotechnologists specifically trained to screen *ThinPrep* slides, and will be interpreted by the Costa Rican study cytopathologist, Mario Alfaro, using the Bethesda 2001 classification system.

The clinical management of the study participants will rely on the Costa Rican cytopathology interpretation, with the exception of HSIL noted below. As part of NCI's ongoing quality control efforts (led by cytotechnologist Claire Eklund and pathologist Martha Hutchinson who are affiliated with the Women's and Infants' Hospital in Providence, RI), all slides that read as abnormal in Costa Rica and a 10% sample of the slides read as negative in Costa Rica will be re-screened and re-interpreted in the United States. At the study exit (Month 48), in addition to 100% of slides read as abnormal in Costa Rica and 10% of the slides read as negative in Costa Rica mentioned above, 100% of slides that are read in Costa Rica as negative with evidence of reactive changes and that are concurrently HPV positive by the HCII will be re-interpreted in the United States.(Amended: 13 Nov 2008) The 10% set of negatives selected for evaluation in the

United States will be randomly selected, without replacement. This scheme will result in approximately 50% of subjects with a negative cytology having an expert review over the course of the 4-year study follow-up period. The purpose of this review is to maximize the accuracy of the Costa Rican interpretations and the comparability of the Costa Rican readings to a US standard, realizing nonetheless that cytopathology is inherently and unavoidably subjective to some extent and that there is no absolute reference standard of cytopathology, particularly for equivocal interpretations such as ASC-US.

The cytological interpretation given by the Costa Rican cytopathologist will be used in clinical management and in the analysis, with one exception. In cases where the Costa Rican interpretation is <HSIL and the US interpretation is HSIL or more severe disease, the interpretation from the US will be communicated to Costa Rica, entered into the CRF, and will lead to colposcopic referral. Also, for clinical management purposes, should *ThinPrep* slides be inadequate for interpretation (estimate <2%), the subject will be managed as if she were LSIL positive, rather than delay management by recall of the patient for repeat cytology.

3.19.2. Histology

(to define trial endpoints)

Biopsy and excisional treatment (loop electrosurgical excisional procedure, or LEEP) specimens obtained during the trial will be fixed in buffered formalin, and sent to the Costa Rican pathologist, Diego Guillen, for processing and diagnosis. LEEP specimens will be sectioned extensively, and up to three levels will be cut from each block. The diagnosis made by the Costa Rican pathologist will be used to determine clinical management of participants.

In addition to the Costa Rican diagnosis, ***an*** NCI ***designated*** pathologist (Amended: 13 Nov 2008***, Amended: 10 September 2009***) with expertise in cervical pathology will periodically diagnose all histological specimens, blinded to the initial Costa Rican diagnosis. If the diagnoses are concordant (concordance will be required at the level of negative or atypical metaplasia/CIN1/CIN2/CIN3 or *in situ*/cancer), that will establish the NCI provisional study diagnosis. Discrepant findings between the Costa Rican and NCI pathologist will lead to review by the second NCI pathologist. Agreement between two of the three pathologists at the <CIN2/CIN2+ level will establish the NCI study diagnosis. These diagnoses will not be available within a timeframe that would allow their use for clinical management purposes. Therefore, NCI pathology review will be performed to define histological diagnoses for analysis purposes. This review process and its results will be performed and documented in the US. A copy of the results of the US pathology review will be sent to the clinical staff in Costa Rica. Periodic review of proficiency slide sets will be performed throughout the trial to assure comparability of the Costa Rican/NCI panel consensus readings against other expert pathologists involved in HPV vaccine studies.

The primary trial endpoint is CIN2+ (associated with HPV16 and/or HPV18). CIN2 and CIN3 will be separately diagnosed for scientific interest, but combined as study endpoints. No cancers are anticipated and, in our experience in Guanacaste,

adenocarcinoma *in situ* will be extremely rare. If these rare outcomes do occur, they will be included as cases.

3.19.3. PCR typing of HPV DNA from *PreservCyt* specimens

(to define trial endpoints)

Two 0.5 mL aliquots of *PreservCyt* specimens will be withdrawn *prior* to *ThinPrep* preparation to avoid possible specimen-to-specimen contamination. The removal of the two small aliquots is not expected to impact on the cellular adequacy of the *PreservCyt* specimen, which typically contains many more cells than required for a *ThinPrep* and subsequent Hybrid Capture® 2 (a signal amplification assay; manufactured by Digene Corporation, Gaithersburg, MD, USA) (HCII HPV DNA test) testing.

The PCR testing used to establish the presence of HPV16 and/or HPV18 for the efficacy endpoints will be performed by GSK Biologicals or DDL Diagnostic Laboratories (Amended: 13 Nov 2008) on one *PreservCyt* aliquot, as follows (see also Appendix C).

Formatted: English (U.S.)

To test for HPV DNA positivity, GSK Biologicals or DDL Diagnostic Laboratories (Amended: 13 Nov 2008) will use SPF_{10} primers that amplify a 65 nucleotide region of the HPV L1 gene for most of the known HPV isolates. The read-out for this test is a DNA enzyme linked immunoassay. Positive specimens will be typed by reverse hybridization line probe assay (LiPA) using 25 type-specific hybridization probes (14 oncogenic HPV types [HPV types 16, 18, 31, 33, 35, 39, 45, 51, 52, 56, 58, 59, 66, and 68] and 11 non-oncogenic HPV types [HPV types 6, 11, 34, 40, 42, 43, 44, 53, 54, 70, and 74]). If LiPA detects no HPV16, the specimen will be tested by HPV16 specific PCR using primers that amplify a 92 nucleotide segment of the E6/E7 gene. If LiPA detects no HPV18, the specimen will be tested by HPV18 specific PCR using primers that amplify a 126 nucleotide segment of the L1 gene. Redundant testing using general PCR with LiPA, followed by type-specific PCR to confirm absent HPV16 and HPV18 affords maximum test sensitivity while preserving high specificity. To monitor laboratory reproducibility, NCI will insert masked QC replicates in each batch sent for testing. The results of this testing algorithm will be considered definitive for all HPV16 and HPV18 related study endpoints.

In order to establish the presence of an HPV type other than HPV16 or HPV18, the second 0.5 mL aliquot of *PreservCyt* will be sent by NCI to a collaborating contract laboratory to test for a full range of genital HPV types using PGMY primers and reverse line blot typing assay (LBA). To monitor laboratory reproducibility, NCI will insert masked QC replicates in each batch sent for testing. Samples positive for HPV types other than HPV16 and HPV18 in either the PGMY/LBA or SPF/LiPA assay will be considered as HPV positive for the appropriate other HPV type.

3.19.4. PCR typing of HPV DNA from histopathological specimens

(to define outcome for secondary objective #4 defined in Section 2.3.)

Tissue-based HPV PCR testing will be performed on sections obtained from formalin-fixed & paraffin-embedded tissue specimens collected from participants referred to colposcopy who are found to have histopathological evidence of CIN2+.

Clean techniques will be implemented when sectioning tissue blocks to avoid contamination. Furthermore, lesional material will be selected for analysis using micro-dissection techniques, as appropriate. Testing will be performed at a laboratory designated by NCI and GSK Biologicals after assay validation.

To test for HPV DNA, SPF_{10} primers which amplify a 65 nucleotide region of the HPV L1 gene for most of the known HPV isolates will be used. The read-out for this test is a DNA enzyme linked immunoassay. Positive specimens will be typed by reverse hybridization line probe assay (LiPA), using 25 type-specific hybridization probes. This typing process enables detection of 14 oncogenic HPV types (HPV-16, 18, 31, 33, 35, 39, 45, 51, 52, 56, 58, 59, 66 and 68) and 11 non-oncogenic HPV types (HPV-6, 11, 34, 40, 42, 43, 44, 53, 54, 70 and 74). If LiPA detects no HPV16, the specimen will be tested by HPV16 specific PCR using primers that amplify a 92 nucleotide segment of the E6/E7 gene. If LiPA detects no HPV18, the specimen will be tested by HPV18 specific PCR using primers that amplify a 126 nucleotide segment of the L1 gene. Redundant testing using generic PCR with LiPA, followed by type-specific PCR to confirm absence of HPV-16 and HPV-18 affords maximum test sensitivity while preserving high specificity. To monitor laboratory reproducibility, NCI will insert QC control specimens as appropriate. The results of this testing algorithm will be considered definitive for the study endpoint used to assess secondary objective #4 defined in Section 2.2

3.19.5. HPV DNA Testing by *Hybrid Capture 2* (HCII)

(for baseline/exit assessment and triage of ASC cytology)

After *ThinPrep* cytology slides have been prepared, residual *PreservCyt* specimens on slides read as ASC will be sent to the HPV DNA testing laboratory (Enrique Freer) at the University of Costa Rica for HPV testing using the *Hybrid Capture 2* test (HCII; Digene Corp., Gaithersburg, Maryland, USA). The assay uses a cocktail of labeled RNA probes (Probe B) designed to detect 13 oncogenic HPV types (HPV types 16, 18, 31, 33, 35, 39, 45, 51, 52, 56, 58, 59, and 68).

An ASC-US (Amended: 29 Mar 2005) cytologic interpretation will lead to “reflex” HCII testing and for the purposes of clinical management subjects who have HCII positive (or HCII-inadequate) ASC-US will be treated the same as an LSIL. Should residual *PreservCyt* material be insufficient for HCII testing, or should HCII testing fail for any other reason, specimens will be assumed to be HCII positive for clinical management purposes and treated accordingly (<5% expected).

HCII testing will also be performed on all subjects at baseline for NCI research purposes and at exit (Month 48 visit) to assist in defining the exit management strategy.

3.19.6. Chlamydia trachomatis & Neisseria gonorrhoeae Testing (Hybrid Capture 2, HCII)

(for baseline assessment)

Hybrid Capture 2 CT/GC (*Hybrid Capture 2 Chlamydia trachomatis* and *Neisseria gonorrhoeae*, Digene Corporation, Gaithersburg, Maryland, USA) is an assay used for the detection of *Chlamydia trachomatis* and *Neisseria gonorrhoeae* in cervical specimens. The test uses specific RNA probes to detect *Chlamydia trachomatis* and *Neisseria gonorrhoeae* DNA in cervical specimens. The RNA/DNA hybrids are detected using a monoclonal antibody specific for RNA/DNA hybrids and readouts are made using chemiluminescent markers.

Testing will be performed according the manufacturer's specifications at the time of each woman's first pelvic examination for NCI research purposes and as a benefit to study participants. The same cervical sample used for the HCII-HPV assay will be used for the HCII CT/GC assay.

3.19.7. Serology Testing by ELISA

Serological assays for HPV16/18 by ELISA will be performed at GSK Biologicals laboratories, Rixensart, Belgium or in a laboratory sponsored by the NCI in Costa Rica, pending validation. Baseline ELISA anti-HPV16 and anti-HPV18 testing will be performed on all women enrolled. In addition, anti-HPV16 and anti-HPV18 ELISA will be performed on all blood samples (Month 0 through Month 48) collected from the 600 women enrolled into the immunogenicity subcohort within the (Amended: 19 May 2006) trial. Women in this immunogenicity subcohort will have an extra clinic visit performed a minimum of 30 days following the last vaccination (Month 7 visit) to permit immunological assessment after all three vaccine doses are administered.

A 1 mL aliquot of serum obtained from 10 mL whole blood will be used for HPV16/18 serology testing. Quantitation of antibodies to HPV16 and HPV18 VLPs (anti-VLP-16 and anti-VLP-18) will be performed by ELISA using either HPV16 or HPV18 VLPs as coating antigens. ELISA titers of VLP-specific antibodies will be calculated relative to the optical density readings obtained with serial dilutions of a positive control pooled reference serum sample using a four-parameter equation. Midpoint titers (expressed in EL.U./mL) will be obtained by averaging the values from all dilutions that fall within the working range of the standard positive control titration curve.

In case of insufficient blood sample volume to perform assays for all antibodies, the samples will be analyzed according to the following priority ranking (ranked in order of decreasing priority):

1. HPV16 antibody (by ELISA)
2. HPV18 antibody (by ELISA).

Serology testing of follow-up specimens collected from women not included in the 600-woman immunogenicity subset or testing for additional antigens contained in the study vaccines will be performed if deemed necessary by the NCI, or if any findings in the

present study or in other studies necessitate additional investigation of the immunogenicity of the vaccine. In this case, the ranking above may also be changed.

3.19.8. V5/J4 Monoclonal Inhibition Enzyme Immunoassay (EIA) Antibodies on a Subset of Subjects

V5/J4 monoclonal inhibition EIA will be performed at GSK Biologicals laboratories, Rixensart, Belgium or in a laboratory designated by NCI and GSK Biologicals after assay validation on all blood samples (Month 0 through Month 48) collected from the 600 women enrolled into the immunogenicity subcohort of the trial.

These EIA inhibition assays are based on the indirect measure of serum antibodies specifically directed either against V5 or J4 epitopes that are described in the literature as the major neutralizing epitopes of HPV16 and HPV18, respectively.

These assays are based on the competitive test principle. Briefly, HPV16 or 18 VLPs are coated onto 96-well microplates, serial dilutions of human sera are added to the VLP-coated plates and incubated. After washing, biotinylated V5 or J4 monoclonal antibodies are added into the wells. The plate is washed and peroxidase (POD)-conjugated streptavidin is added. The binding of streptavidin is detected by the addition of tetra methyl benzidine (TMB). Colorimetric change induced will be inversely proportional to the concentration of anti-V5 or -J4 antibodies in the serum. ELISA titers are calculated from a reference curve using a four parameters equation and expressed in ELISA Units per milliliter (EU/mL).

3.19.9. Other Assays

Several other laboratory assays will be performed for research purposes to enable exploratory analyses of tertiary objectives and ancillary studies.

3.20. Efforts to Maximize Participation/Retention

Although a randomized clinical trial is the ideal method to assess the effectiveness of any intervention, it is well recognized that subject withdrawal or loss-to-follow-up subsequent to randomization can introduce bias if losses are differential in the different trial arms. Thus, issues of retention subsequent to enrollment are important and have been given emphasis in planning for the present trial.

Efforts aimed at minimizing withdrawal and losses during follow-up are listed below:

- Selection of Guanacaste as the trial site because of high participation and retention rates in previous studies.
- Community outreach and education efforts are planned to assure that adequate information regarding the trial is disseminated throughout the region. These efforts will target both the population at large and the public health community in the region.

- We will invite 20,000 women to participate, but our power estimates (see Table 6 Section 4.7) indicate the need for 7,500 women (Amended: 13 Nov 2008). Only women who, in principle, agree to comply with the full protocol will be enrolled.
- We plan to send reminder letters before each appointment. For appointments preceding vaccination visits we will also send an outreach worker to the home of participants to remind them of their scheduled appointments.
- Reimbursement for transportation costs will be provided, if necessary and, when needed, transportation to the clinic in a study vehicle will be provided.
- Women who, at enrollment, have definite plans to move out of the study area within six months after enrollment will be excluded.
- Women who move within Guanacaste will be given the option to have their clinic assignment changed to a clinic closer to their new home. Women who work will also have the option to be seen at a clinic that is close to their work location. Several clinics throughout Guanacaste and surroundings will be used for our trial, allowing for such flexibility.
- Despite our attempt to exclude women with plans to migrate outside of our study region, some women are likely to move during the four-year study follow-up period. Women who move will be offered transportation or reimbursement of expenses to visit the nearest study clinic. During our Natural History Cohort study women who had migrated outside of Guanacaste often agreed to return to our clinics for their appointments.

Efforts described above should minimize withdrawals from the study prior to the completion of 48 months of follow-up. In the event that a woman withdraws from the study due to participant refusal, relocation, death, or other reason, the specific reason for withdrawal will be carefully specified on a CRF designed for this purpose. Discontinuation of vaccination (i.e., withdrawal from vaccination) will not constitute grounds for withdrawal from study follow-up. The occurrence of an AE will also not constitute grounds for withdrawal. However, should a woman choose to refuse further participation as a result of an AE, this will be carefully documented and all attempts will be made to track the AE to its resolution.

4. STATISTICAL ANALYSIS PLAN & POWER

4.1. Overview

The main analyses planned for our trial will be conducted by the NCI and Costa Rican investigators and will focus on the primary outcome, viz., HPV16 or HPV18 (combined) associated, histopathologically confirmed CIN2+ among uninfected individuals (i.e., HPV DNA negative for HPV16 or HPV18 at entry and at 6 months), as defined in Section 2. The primary analyses will be based on the difference in the cumulative proportions of women who develop an outcome between the treatment group and the control group (i.e., difference between attack rates). The test statistic will be the Fisher's exact test for difference between two proportions. Analysis will be triggered by the total number of events observed, and will be conducted after at least 30 confirmed cases are

observed and the last woman recruited into the study has completed a follow-up time of two years, pending review and approval of the final statistical analysis plan by the DSMB.

The primary analysis for this trial will be “According-to-Protocol” (ATP). The ATP analysis will exclude women otherwise eligible for the trial who do not receive all three doses of vaccine or who test positive for HPV16 and HPV18 DNA before or at the time of the last vaccination (scheduled for 6 months after randomization). An Intent-to-Treat (ITT) analysis is also planned to confirm ATP findings. For the ITT analysis, all women randomly assigned to one of the two study arms and confirmed by HPV DNA testing to be HPV16 or HPV18 negative at randomization will be included.

Secondary analyses for efficacy will be performed to examine duration of the effect of the vaccine in the prevention of incident infection with HPV16 or HPV18. These analyses will focus on events that occur at one, two, three, and four years after randomization in women who are under follow-up and had not previously had the event of interest. Additional secondary analyses will evaluate the efficacy of the candidate vaccine in the prevention of CIN2+ associated with all incident oncogenic HPV types including HPV16 and HPV18, in the prevention of persistent HPV16 or HPV18 infection (persistence defined as 12+ months of infection with same type infection). Analyses of the 600 women in the immunogenicity subset will also be performed and will focus on defining the serological antibody profile observed in vaccinated individuals over time.

Additional exploratory tertiary and ancillary analyses will be conducted by the NCI and Costa Rican investigators and will evaluate numerous issues, as described in the Tertiary Objectives and Ancillary Analyses & Studies sections of this protocol (Section 2.3 and Section 2.4).

No interim statistical analyses for ***the primary*** efficacy ***endpoint*** are planned at this time ***(Amended: 10 September 2009)***.

Adverse events (AEs) observed in the course of the trial will be carefully monitored. Both expected and unexpected AEs will be examined. Rates of specific solicited, unsolicited, and serious AEs (SAEs) will be compared by treatment arm and evaluated. These analyses will be performed throughout the trial by the DSMB (study personnel will remain blinded) and will not affect the overall alpha level of the study.

Power calculations are provided at the end of this section (Section 4.7). Assumptions inherent in our calculations are specified and are based largely on results of our ongoing population-based natural history study in Guanacaste. Assuming conservatively that 12,000 (6,000 per arm) of the 20,000 women invited to participate are randomly assigned to one of the two arms of the trial, we estimate an overall power of 91% to detect an 80% reduction in incidence of HPV16 or HPV18 (combined) associated CIN2+ over the four years of follow-up planned for this trial.

4.2. Primary Analysis for Efficacy

The primary analysis will assess the candidate vaccine's ability to protect against incident, histopathologically confirmed HPV16 or HPV18 associated CIN2+ among uninfected individuals (i.e., HPV DNA negative for HPV16 or HPV18 at entry and at 6 months). An expert panel of pathologists will review all histopathological specimens blinded to vaccination status to determine CIN2+ diagnoses. Presence of HPV16 or HPV18 DNA will be determined by testing cervical cells collected into liquid-based *PreservCyt* medium by PCR using the SPF_{10} primers typed using the LiPA method and followed by HPV16 and HPV18 type-specific primers (Section 3.19). For women with multiple type infections including HPV16 or HPV18, we will assume that HPV16 or HPV18 is associated with the CIN2+ lesion.

Final analysis for Biologic License Application (BLA) submission will be triggered when one of the following sets of conditions is met:

- 48 months of follow-up per participant is achieved, or
- At least 30 confirmed cases of CIN2+ associated with HPV16 or HPV18 cervical infection that are eligible for the ATP analysis are observed and the last woman recruited into the study has completed a minimum follow-up period of two years, pending review and approval of the final statistical analysis plan by the DSMB. (Amended: 29 Mar 2005)

Assuming 15,000 women enroll, it is expected that a total of approximately 50 HPV16/18+ CIN2+ events will occur across both trial arms over the course of the trial under the null hypothesis. A minimum threshold for analysis of 30 events was chosen since this number of events would provide adequate power (96%) to observe a statistically significant difference between the two trial arms under the assumption of 80% efficacy (Table 6). The requirement of a minimum (Amended: 29 Mar 2005) follow-up time of two years was chosen to guard against analyses that are biased by early events only, which in the case of CIN2+ among young women might not be representative of true cancer precursors.

Should analysis occur prior to completion of the full 48 months of follow-up (under the second scenario above), women will continue to be followed through 48 months and an annex report will be issued to describe any additional data collected.

The main analysis of this primary endpoint will compare the cumulative proportions (i.e., attack rates) of women who develop an event among those who were randomly assigned to one of the two arms of the trial and found to be HPV16 or HPV18 DNA negative and without CIN2+ at baseline.

4.2.1. Baseline Characteristics

An initial evaluation will examine baseline characteristics (age, residence, education, virginity) of each trial group to assure comparability between groups.

4.2.2. Exclusion from Analysis Based on Pre-Enrollment Testing

Women will be tested at entry for the presence of HPV16 or HPV18 DNA; only those who test negative for HPV16 or HPV18 will be included in the main ATP and ITT analyses described below. Women infected with HPV16 and HPV18 DNA prior to vaccination will be excluded from the main analysis to focus the analysis on the prophylactic (not therapeutic) efficacy of our vaccine. All decisions about presence of HPV16/18 at baseline will be made by protocols that ensure blindness to assigned treatment arm and any information or materials collected at or subsequent to randomization.

4.2.3. According to Protocol (ATP) Analysis

The primary analysis will be an ATP analysis. Criteria for elimination from the ATP 3.3.4 analyses are listed in Section of this protocol. ATP analysis will be performed using a Fisher's exact test for equality of proportions. Kaplan Meier analysis will also be performed (see plans for secondary analyses below). The log-rank test is not being proposed for this primary analysis since a vaccine that significantly delays onset of the primary outcome without significantly affecting the cumulative difference in attack rates should not be considered an efficacious vaccine.

4.2.4. Intent to Treat (ITT) Analysis

An ITT analysis will also be performed to confirm the ATP findings. For the ITT analysis, all eligible women who consent and are randomly assigned to one of the two arms of the trial (and all events occurring after randomization) will be included, regardless of whether or not all three doses of the HPV16/18 VLP or control vaccine are administered.

4.3. Secondary Analyses for Efficacy

4.3.1. Duration of Protection

An important objective of this trial is to evaluate the duration of protection provided by the candidate vaccine. Short-term protection from the vaccine is sufficient to provide evidence for efficacy and will constitute the primary justification for licensure. However, evidence of longer-term protection is necessary to justify vaccination as a viable alternative to currently available screening practices for the prophylaxis of cervical cancer, particularly in developing countries. Therefore, we will consider analyses aimed at evaluating long-term efficacy of the vaccine in the prevention of incident infection with HPV16 or HPV18. To this end, we plan to examine Kaplan Meier curves and stratified log-rank test among those free of the outcome (left-truncation) at one-, two- and three-years after completion of vaccination (for ATP analyses) and randomization (for ITT analyses).

4.3.2. Protection against Any Oncogenic HPV Types

An additional important secondary objective of our trial is to evaluate whether the candidate vaccine protects against incident CIN2+ associated with any oncogenic HPV type. Both ATP and ITT analyses will be conducted, using the same methods proposed for the primary and secondary analyses described above (i.e., comparison of attack rates and of Kaplan Meier curves).

4.3.3. Protection against HPV16 or HPV18 Associated CIN2+ Among Women with no Evidence of Exposure to HPV16 or HPV18 at Entry

To more stringently evaluate whether the candidate vaccine protects against incident, HPV16 or HPV18 associated CIN2+ among unexposed individuals, secondary analyses will be performed restricted to women who are not only HPV DNA (by PCR) negative at months 0 and 6, but also seronegative by ELISA at month 0 for the corresponding HPV type. In addition, HPV16 or HPV18 positivity will be defined by PCR on the lesional component of the cervical tissue specimen. Statistical analysis methods identical to those proposed for the primary analysis will be used for this purpose.

4.3.4. Protection against Persistent HPV16 or HPV18 Infection

To confirm published findings that immunization with VLP-based HPV vaccines protects against persistent infection, secondary analyses will also be performed to evaluate whether the candidate vaccine protects against long-term type-specific persistence (defined as 12+ months of infection) with HPV16 or HPV18. Both ATP and ITT analyses will be conducted, using the same methods proposed for the primary and secondary analyses described above (i.e., comparison of attack rates and of Kaplan Meier curves).

4.3.5. Vaccine Immunogenicity

To directly assess HPV16/18 VLP vaccine immunogenicity (as measured by anti-HPV16 and anti-HPV18 ELISA and V5/J4 monoclonal antibody tests) in our population, secondary analyses will be performed with the specific intent of confirming that HPV16 immunogenicity in our population mirrors that seen in other Phase I/II trials conducted to date, evaluating whether HPV18 immunogenicity seen after vaccination is comparable to that seen for HPV16, and evaluating patterns of antibody levels over time since vaccination.

4.4. Evaluation of Adverse Events (AEs)

In addition to vaccine efficacy, we plan to carefully monitor and evaluate AEs observed in the course of the trial. Our plan for detecting, documenting, and classifying AEs is discussed in Section 3.14. In this section we describe our plans for evaluation of these AEs. Analysis of AE data will be based on the total vaccinated group without exclusions. An additional analysis of safety will be performed based on an ATP safety cohort. The ATP safety cohort will be defined as all subjects who have received at least one dose of

study vaccine/control according to their randomization assignment (i.e., excluding individuals who received no vaccination or who received a vaccine other than the one specified by randomization), who did not receive a vaccine forbidden in the protocol, for whom the administration site is known, and for whom sufficient data is available to perform an analysis of safety. It should be noted that for these analyses to evaluate AEs, study personnel will not be unblinded, analyses will initially be presented to the DSMB by group without specifying which group is the vaccinated or control group, and unblinding of the groups by the DSMB will only occur at the request of the DSMB should safety issues arise.

Both expected AEs, as defined in the GSK Investigator Brochure, and unexpected AEs will be evaluated. Solicited, unsolicited, and serious AEs (SAEs) will be evaluated separately. AEs and SAEs will be evaluated throughout the study. SAEs will be reported to the DSMB immediately, whereas non-serious AEs will be summarized and reported to the DSMB on a periodic basis. Expedited reporting of SAEs to the US FDA, other national regulatory authorities, and Investigators will be performed by GSK, in accordance with relevant regulations. Details of the plan for US FDA reporting are provided in Appendix D. Our plan for the analysis of AEs by the DSMB is summarized below.

1. Descriptive analysis will be performed to quantify the proportion (along with 95% confidence intervals) of vaccinated women who experience specific AEs. Solicited and unsolicited AEs will be tabulated separately by intensity. SAEs will also be evaluated separately. Relationship to vaccination will be examined. The proportion of individuals ever experiencing AEs and the proportion experiencing AEs at specific times (e.g., proportion of vaccinated women who experience pain at the injection site on the day of vaccination and in the week following vaccination) will be computed. Analysis stratified by dose number will also be performed.
2. The proportion of women who experience specific solicited AEs, unsolicited AEs, and SAEs will also be compared between the vaccinated group and the control group to determine whether there is evidence for significantly increased reactogenicity to the vaccine compared to control. Again, both cumulative and time-specific proportions will be computed, as will analysis stratified by dose number.
3. Pregnancies and pregnancy outcomes will be monitored throughout the study, and comparisons between the vaccine and control groups will be carried out in cumulative and time specific analyses.
4. Finally, since the incidence of new or exacerbated chronic illnesses with a possible immune pathogenesis is elevated among women 18-25 years of age, separate analyses will focus on an evaluation of new or exacerbated chronic illnesses such as autoimmune diseases (e.g., systemic lupus erythematosus, thyroiditis), insulin-dependent diabetes mellitus, and asthma. A similar approach to that described above will be used for this assessment.

4.5. Other Analyses

In keeping with NCI’s research mission, we plan multiple analyses to evaluate issues of scientific relevance to the research community in addition to the analyses summarized

above. These include analyses of vaccine effects using Cox regression that are stratified by specific subpopulations (e.g., seronegative and seropositive individuals at entry; users and non-users of oral contraceptives), analyses restricted to women who are virgins at baseline and therefore have never been previously exposed to HPV16 or HPV18. NCI will also perform analyses that adjust for any effect of these factors if they are not distributed in a balanced way between vaccination groups despite randomization. Additional analyses will look at endpoints for evaluation of HPV16 and HPV18 effects separately, of the ability of the candidate vaccine to protect against transient and persistent infections with HPV16 and HPV18, and of the therapeutic effect of the candidate vaccine among HPV16 or HPV18 positive individuals at entry. The large size of the present trial (including the large number of women who will receive control vaccinations) also provides us the unique opportunity to evaluate various hypotheses related to the natural history of HPV infection and SIL and to immunological response to HPV and to HPV vaccination. These ancillary analyses will be performed by NCI and Costa Rican investigators to increase our knowledge of HPV, cervical neoplasia, and immune response rather than to evaluate the efficacy of the vaccines to protect against the primary trial outcomes. Therefore, although results from these analyses will be shared with the DSMB and with the research community as a whole, they will not necessarily be used to determine efficacy of the vaccine. A detailed description of these natural history and etiological analyses is beyond the scope of this protocol.

4.6. Interim Analysis

There will be no restrictions on the number of analyses that can be conducted by the DSMB to examine safety of the candidate vaccine. Unscheduled analyses of safety may be conducted by the DSMB and will not affect the alpha for testing vaccine efficacy. Safety concerns with the vaccine shall constitute grounds for stopping enrollment or vaccination at any time during the trial.

No interim statistical analyses for efficacy ***were*** planned ***at the beginning of the trial (Amended: 10 September 2009)***. A need for an interim analysis for efficacy may arise during the trial. Should this occur, the study protocol will be amended, and the amendment submitted to the US FDA for review. To assist the DSMB in making decisions regarding interim analyses in the future, a set of guidelines are provided separately from this protocol in the DSMB Charter.

A Statistical Analysis Plan (SAP) for initial analyses that focuses on virological endpoints has been submitted to the FDA. The analyses proposed within are largely exploratory in nature, but there is one overlap between an objective in the SAP for initial analysis and secondary objective #5 listed in this protocol (i.e., for secondary objective 5 listed in this protocol, we are proposing to conduct an interim analysis). Given this overlap, for secondary objective #5 listed in this protocol, the following alpha adjustment plan will be implemented: The overall alpha of 0.025 (one-sided) will be split into 0.001 for the interim analysis proposed in the SAP for initial analyses and 0.024 for the final analysis. As specified in more detail in the SAP for initial analysis (Table 3 of that document), power (assuming 80% vaccine efficacy and 20 or more total events) should remain above 85-90% for the final analysis of secondary objective #5 (Amended: 10 September 2009).

No stopping rule will be applied at this stage; blinding will be maintained and follow-up will continue for all subjects until Month 48 (Amended: 10 September 2009).

4.7. Power to Determine Vaccine Efficacy

The powers of our trial to detect vaccine efficacy rates ranging from 30-90% and for sample sizes ranging from 10,000-15,000 are summarized in Table 6. These calculations were performed using the PASS program (Hintze J. 2001. NCSS and PASS Number Cruncher Statistical Systems, Kaysville, UT, USA). The assumptions used in estimating power were based largely on results from our population-based natural history study in Guanacaste, Costa Rica [Herrero, 2000] and from the British natural history study published by Woodman and colleagues [Woodman, 2001]. We estimated the expected person-years and rates as follows for the ATP analysis assuming 15,000 women randomly assigned to one of the two arms of the trial:

1. **Person-years**: A total of 3,087 person-years/1,000 evaluable women are expected over the course of the four-years of follow-up in the control group. The expected person-years/1,000 evaluable women in the HPV16/18 VLP vaccine group is slightly higher (3,090/1,000 evaluable women) since we expect fewer CIN2+ events that terminate a woman’s follow-up in the HPV16/18 VLP vaccine arm. These estimates account for the exclusion of person-years accrued:
    - by women in the first 6 months of the study (since events in that time are excluded from ATP analysis),
    - by women exposed to both HPV16 and HPV18 at entry or at the time of the third vaccination (month 6),
    - by women diagnosed with CIN2+ in the first 6 months of the study,
    - by women who do not receive all three doses of vaccine,
    - by women who are virginal since virginal women are not at risk of CIN2+,
    - by women who are lost to follow-up during the study
2. **Outcome Rates**: The expected rate of histopathologically confirmed CIN2+ associated with HPV16 or HPV18 occurring over the 42 months post third vaccination follow-up period among evaluable women is estimated to be 0.5% (.00238 annual rate of CIN2+ * 0.6 proportion of CIN2+ associated with HPV16/18 * 3.5 for 42 months of follow-up in ATP).

To estimate the person-years and rates described above, the following specific assumptions were made, based largely on data from our natural history study in Costa Rica. All of the assumptions used to calculate power are conservative to avoid underpowering our study.

1. The annual incidence of histopathologically confirmed CIN2+ is estimated to be 0.238%.
2. The percent of non-virginal women with histopathologically confirmed CIN2+ at enrollment is estimated to be 1.5%.

3. The percent of CIN2+ with detectable HPV16 or HPV18 DNA is estimated to be 60%.
4. The proportion of women who are sexually inexperienced at entry is estimated to be 23.0%. The proportion of women who are sexually experienced at entry is estimated to be 77.0%.
5. The proportion of virginal women who initiate sexual activity during follow-up is estimated to be 43.2%. We assumed that initiation of sexual activity among virginal women at entry will occur evenly throughout the four years of follow-up.
6. The proportion of these sexually experienced women who are both HPV16 DNA positive and HPV18 DNA positive at entry is estimated to be 1%.
7. The proportion of sexually experienced women who remain HPV16 or HPV18 negative at the end of the vaccination phase (month 6) among those who are HPV16 and HPV18 DNA negative at entry is 98.5%.
8. The proportion of women diagnosed with CIN2+ in the first 6 months of the study is estimated to be 0.2%.
9. We expect that 10% of women will receive less than three vaccine doses.
10. Women will be followed for a total of four years from first vaccination.
11. We expect 10% of participants to be lost to follow-up during our study. The dropout rate is expected to be evenly distributed throughout the four years of follow-up.
12. Two-sided alpha of 0.05.

Table 6 Power to Determine Vaccine Efficacy in the Costa Rican Trial

Sample Size	Cumulative # HPV16/18 CIN2+ in control/vaccine arms		% Efficacy	Power* to exclude 0% Efficacy	Power* to exclude 30% Efficacy**
	Control	Vaccine			
10,000	17	12	30%	16%	2%
12,000	20	14	30%	18%	2%
15,000	25	18	30%	21%	2%
10,000	17	8	50%	39%	12%
12,000	20	10	50%	45%	14%
15,000	25	13	50%	53%	16%
10,000	17	3	80%	85%	55%
12,000	20	4	80%	91%	63%
15,000	25	5	80%	96%	73%
10,000	17	2	90%	94%	67%
12,000	20	2	90%	97%	75%
15,000	25	3	90%	99%	84%

*Alpha (2-sided) = 0.05.
**Power calculations to exclude efficacy of 30% were performed using an NCI Matrix Laboratory (MATLAB®, Mathworks, Inc.)-based program.

Total accrual into the Costa Rica Trial (HPV-009) was lower than initial estimates. For the outcome of histological CIN2+ (expected # events = 24), the final accrual of 7,466 provides 91% power to detect a vaccine efficacy of 80% under the following assumptions:

1) cumulative incidence of histopathologically confirmed CIN2+ associated with HPV-16 or HPV-18 of 0.79% (Woodman, 2001)
2) percent of non-virginal women with histologically confirmed CIN2+ at enrolment of 1.5%
3) proportion of women who are sexually inexperienced at entry of 23%
4) Proportion of virginal women who initiate sexual activity during follow-up of 43% (constant rate of initiation assumed during the 4-year follow-up period)
5) proportion of sexually experienced women who are both HPV-16 and HPV-18 DNA positive at entry of 0.6%
6) proportion of sexually experienced women who acquire HPV-16/18 during the vaccination phase of 1.5% (includes women who develop incident CIN2+ associated with HPV-16/18 infection during this period)
7) proportion of women who receive 1+ vaccine doses outside the prescribed protocol window of 20%
8) proportion of women who drop out during the study period of 10% (constant drop-out rate assumed during the 4-year follow-up period)
9) a two-sided alpha of 0.05. (Amended: 13 Nov 2008)

5. OVERSIGHT BY THE DATA AND SAFETY MONITORING BOARD & ADVISORY BOARD

A Data and Safety Monitoring Board (DSMB) has been established by NCI to review and monitor the trial. The Charter Document for this DSMB is provided separately from this protocol. The DSMB membership consists of individuals with expertise in statistics, gynecology, pathology, clinical trials, and health advocacy. A list of the DSMB members is provided separately from this protocol in the DSMB Charter. Members of the DSMB are from outside the NIH to assure an independent review of the trial. DSMB members have no conflicts of interest with pharmaceutical companies currently involved in the development of prophylactic HPV vaccines. The DSMB is composed of both United States and Costa Rican nationals to assure joint review of the trial by representatives of both countries involved.

The primary role of the DSMB will be to:

- Monitor accrual and event rates to confirm that assumptions underlying the trial design are adequate.
- Monitor adverse event data at yearly intervals, or more frequently if needed, to assure the safety of participants.
- Monitor efficacy, should the need for an interim analysis arise, considering the guidelines which are provided separately from this protocol as part of the Charter Document for the DSMB. It should be noted that no interim analysis is planned at present.

After each meeting the DSMB will submit its recommendations to the NCI Principal Investigator, the DCEG Scientific Director, the Costa Rican Principal Investigator, and the NCI and Costa Rican IRBs. The NCI Principal Investigators will share these recommendations with GSK Biologicals.

In addition to the DSMB, the NCI has formed an External Advisory Group comprised of experts in virology, vaccinology, gynecology, oncology, pathology, epidemiology, statistics, medical ethics, and health advocacy. This group will advise the NCI Principal Investigator and the DCEG Scientific Director on other issues that will undoubtedly arise during the course of the trial. For example, the External Advisory Group may be asked to:

- Review and approve the trial protocol and procedures prior to initiation of the trial.
- Review concepts for ancillary studies to assure that their implementation will not negatively impact the primary or secondary objectives of our trial.
- Review major publications relating to the primary or secondary objectives of our trial.

Members of the External Advisory Group are from outside the NIH to assure independence. Members have no conflicts of interest with pharmaceutical companies currently involved in the development of prophylactic HPV vaccines. The External Advisory Group is composed of both United States and Costa Rican nationals to assure joint review of the trial by representatives of both countries involved.

6. HUMAN SUBJECTS PROTECTION

The HPV16/18 VLP Vaccine Trial will be conducted in compliance with regulations for human subject research in effect in Costa Rica and the United States (The Belmont Report & Title 45 Code of Federal Regulations, Part 46, Protection of Human Subjects, and Declaration of Helsinki 1996, including its 2000 amendment). (Amended: 29 Mar 2005)]. The principles of respect for persons, beneficence, and justice will be paramount in planning our trial. Participants will be recruited into our trial only after they have been informed about the trial procedures, have understood what is being asked of them, and signed an informed consent form. A draft of the Informed Consent form is provided separately from this protocol. The subject information sheet and written informed consent form as well as the informed consent process employed during our trial will comply with Section 4.8 (Informed Consent of Trial Subjects) of the ICH guideline for Good Clinical Practices. Our trial will be limited to women, since the goal of the trial is to investigate vaccine efficacy against a disease that occurs among women only. Women 18-25 years of age at enrollment will be eligible to participate. The lower age limit of 18 was chosen since this is the age at which individuals in Costa Rica are considered adults and the age at which they can legally self-consent to participation in research projects. Since the US National Institutes of Health (NIH) defines women younger than 21 as children, our trial will include “children” (women ages 18-20 years) based on the US NIH definition.

Trial plans and procedures will be reviewed by the NCI Institutional Review Board (IRB). A local IRB in Costa Rica will also review this project. The Costa Rican IRB

responsible for review of our project is part of INCIENSA (Costa Rican Institute for Research on Nutrition and Health), an institution affiliated with the Ministry of Health. This local IRB is registered with the Costa Rican CONIS (Scientific Research National Council of the Ministry of Health). It is also registered with the US Office of Human Research Protection (IRB# 00001092) and our study will be conducted under a Federal-Wide Assurance (FWA# 00006588) from the Department of Health and Human Services (DHHS).

The trial will also be conducted under Title 21 Part 312 governing the conduct of trials under an Investigational New Drug Application (IND) and Good Clinical Practices (ICH E6).

The trial will be conducted in a manner that maximizes patient confidentiality. A trial-specific patient identification number will be assigned to each participant to avoid use of name or other patient identifiers (e.g., national ID number). Separate specimen identification numbers will also be used (distinct from the patient identification numbers) to provide an additional level of privacy protection. Interviews and clinical examinations will be conducted in a private setting by study staff that have been carefully trained and are aware of the need to maintain patient confidentiality. Data collected as part of this trial will be coded and keyed into a secure data management system developed specifically for this trial. Information that is sent in an expedited manner to NCI and GSK (e.g., SAE reports) will contain only the study-specific identification number and will not contain names or other patient identifiers (e.g., national ID number). Analysis datasets generated for this trial will not contain patient names, and will instead rely on the trial-specific patient and specimen identification numbers.

Since our trial will involve a medical intervention on a large number of women, an independent DSMB has been established to monitor adverse events and trial progress (see Section 5). The DSMB will perform periodic review of adverse events reported during the trial. After each review, members of the DSMB will summarize their findings and will report them to the NCI Principal Investigator and to the NCI and local Costa Rican IRBs. Should the NCI or local IRB have any concerns regarding trial adverse events, these will be addressed directly to the DSMB with a copy to the Costa Rica and NCI Principal Investigator. Full communication between the human subjects review committees and the DSMB is essential and will be the responsibility of the NCI Principal Investigator.

7. VACCINE MANUFACTURING & QUALITY CONTROL

The vaccines to be used in our trial will be manufactured by GSK Biologicals and provided to the NCI under a Clinical Trials Agreement (CTA). The HPV16/18 L1 VLP and control (HAV) vaccines will be manufactured under strict GMP conditions. Three lots of HPV16/18 VLP vaccine have been prepared to support the Phase III efficacy trial. Each HPV16/18 VLP vaccine lot is manufactured using one single batch of HPV16 L1 VLP antigen and one single batch of HPV18 L1 VLP antigen. Four lots of the investigational formulation of *Havrix* control vaccine have been prepared to support the Phase III efficacy trial. Formulation of the *Havrix* control vaccine lots was performed using one batch of purified inactivated Hepatitis A antigen. Individual use syringes will

be prepared and pre-labeled with individual specimen ID numbers. Exhaustive quality control procedures are implemented during the manufacturing, syringe filling, and labeling process, and include an assessment of product purity, identity, strength/potency, and safety. Details regarding vaccine manufacturing and quality control procedures can be found in the GSK Investigator Brochure.

Each lot of vaccine prepared for the Costa Rica trial will undergo extensive stability studies in which both the bulk and final product packaged in syringes will be tested to assure continued purity, stability, and strength/potency of the vaccine. Such testing will be performed at predefined intervals and at temperatures ranging from 37°C to 4°C.

Studies to date indicate that the vaccine is stable for two years when stored at 2°C to 8°C. Moreover, experience during Phase II demonstrated that vaccine handled in the field at more than 50 different study centers in the USA, Canada, and Brazil was highly immunogenic; overall, more than 98% of women receiving 3 doses of HPV16/18 VLP vaccine developed antibodies to HPV16 and HPV18, compared to less than 5% of women receiving 3 doses of control vaccine. Nonetheless, results from stability studies performed at GSK Biologicals on the lots of vaccine manufactured for the Costa Rica trial will be reviewed by the NCI and Costa Rica Principal Investigators on an ongoing basis. Should evidence of >50% VLP disassociation or loss in potency be observed, a new lot of vaccine will be manufactured by GSK Biologicals to replace the original lot in Costa Rica.

8. COLLABORATORS AND CONSULTANTS

A list of collaborators and consultants, the medical and scientific contributions that they will provide to the NCI HPV16/18 VLP Vaccine Trial in Costa Rica, and their institutional affiliations is provided separately from this protocol.

The main collaborating institutions involved in this trial and their role in the trial are summarized below.

U.S. National Cancer Institute (NCI): The NCI, in close collaboration with its Costa Rican collaborators, is responsible for the overall design and implementation of the trial. NCI will also be responsible, in collaboration with its Costa Rican collaborators, for the analysis of data obtained from this trial. The trial is funded by the NCI.

Proyecto Epidemiologico Guanacaste (PEG): PEG investigators, in close collaboration with its NCI collaborators, are responsible for the overall design of the trial and for conducting the field activities required for its implementation. PEG investigators are also responsible, along with its NCI collaborators, for the analysis of data obtained from this trial.

GlaxoSmithKline Biologicals (GSK): Under a Clinical Trials Agreement with the NCI, GSK has agreed to provide the vaccines to be used for the trial. The trial will be conducted under a GSK IND with the US FDA and as such GSK will be responsible for reporting to the US FDA. Data generated by this trial will be used by GSK for submission to regulatory authorities in the United States and elsewhere.

Costa Rica Ministry of Health (MOH): Under a Letter of Intent with the NCI, the MOH has expressed its interest in the trial and its intent to support its implementation.

Costa Rican Institute for Research and Training in Nutrition and Health (INCIENSA): The INCIENSA IRB will be responsible for Human Subjects Review of the trial in Costa Rica.

Westat, Inc.: Under contract with the NCI, Westat will assist NCI and Costa Rican investigators in the development of trial materials and manuals. Westat will also be responsible for monitoring and auditing trial activities, and for tracking the reporting of SAEs and pregnancy information to the NCI and GSK.

Information Management Services (IMS): Under contract with the NCI, IMS is responsible for the development and maintenance of the data management system used for our trial. IMS will also assist NCI in the preparation of reports for the DSMB and other review entities, in running of edit reports on data received from Costa Rica, and in data analysis.

Boston Biomedica Inc.-Biotech Research Laboratories (BBI): Under contract with the NCI, BBI will serve as the primary repository for specimens collected from this trial. BBI will also provide assistance to the NCI and Costa Rican investigators in the set-up and maintenance of the local repository in Costa Rica.

9. PROJECTED TIMETABLE

Review of Protocol by External Advisory Group	Mar ‘03
Submission of Concept Protocol to the FDA	June ‘03
NCI Human Subjects Annual Review	Jul ‘03
Costa Rican Human Subjects Annual Review	Aug ‘03
Submission of CMC & Full Study Protocol to FDA	Jan ‘04
First Batch of Vaccine Doses for Study Delivered to Costa Rica	Apr ‘04
Training	May ‘04
Initiation of Main Trial (Amended: 29 Mar 2005)	June 28, ‘04
Enrollment of Participants (Amended: 29 Mar 2005; 31 Oct 2005)	June ’04 – Dec‘05
Vaccination of Participants (Amended: 31 Oct 2005)	June ’04 – June ‘06
Active Follow-up of Participants (Amended: 29 Mar 2005; 31 Oct 2005)	June ’04 – Dec ‘09
Study Close-out (Amended: 29 Mar 2005; 31 Oct 2005)	Nov ’09 – Apr ‘10

10. REFERENCES

Bosch, F. X., Manos, M. M., Munoz, N., Sherman, M., Jansen, A. M., Peto, J., Schiffman, M. H., Moreno, V., Kurman R., Shah, K. V., and the International biological study on cervical cancer (IBSCC) study group. Prevalence of human papillomavirus in cervical cancer: a worldwide perspective. J Natl Cancer Inst, *87*: 796-802, 1995.

Breitburd, F., Kirnbauer, R., Hubbert, N. L., Nonnenmacher, B., Trin-Dinh-Desmarquet, C., Orth, G., Schiller, J. T., and Lowy, D. R. Immunization with viruslike particles from cottontail rabbit papillomavirus (CRPV) can protect against experimental CRPV infection. J Virol, *69*: 3959-3963, 1995.

Christensen, N. D., Reed, C. A., Cladel, N. M., Han, R., and Kreider, J. W. Immunization with viruslike particles induces long-term protection of rabbits against challenge with cottontail rabbit papillomavirus. J Virol, *70*: 960-965, 1996.

Evans, T. G., Bonnez, W., Rose, R. C., Koenig, S., Demeter, L., Suzich, J. A., O'Brien, D., Campbell, M., White, W. I., Balsley, J., and Reichman, R. C. A Phase 1 study of a recombinant viruslike particle vaccine against human papillomavirus type 11 in healthy adult volunteers. J Infect Dis, *183*: 1485-1493, 2001.

Gravitt, P. E., Lacey J. V. Jr., Brinton, L. A., Barnes W. A., Kornegay, J. R., Greenberg, M. D., Greene, S. M., Hadjimichael, O. C., McGowan, L., Mortel, R., Schwartz, P. E., Zaino, R., and Hildesheim, A. Evaluation of self-collected cervicovaginal cell samples for human papillomavirus testing by polymerase chain reaction. Cancer Epidemiol Biomarkers Prev, *10*: 95-100, 2001.

Harro, C. D., Pang, Y. Y., Roden, R. B., Hildesheim, A., Wang, Z., Reynolds M. J., Mast T. C., Robinson, R., Murphy, B. R., Karron, R. A., Dillner, J., Schiller, J. T., and Lowy, D. R. Safety and immunogenicity trial in adult volunteers of a human papillomavirus type 16 L 1 virus-like particle vaccine. J Natl Cancer Inst, *93*: 284-292, 2001.

Herrero, R., Brinton, L. A., Hartge, P., Reeves, W. C., Brenes, M. M., Urcuyo, R., Pacheo, M., Fuster, F., and Sierra, R. Determinants of the geographic variation of invasive cervical cancer in Costa Rica. Bull Pan Am Health Organ, *27*: 15-25, 1993.

Herrero, R., Hildesheim, A., Bratti, C., Sherman, M. E., Hutchinson, M., Morales, J., Balmaceda, I., Greenberg, M. D., Alfaro, M., Burk, R. D., Wacholder, S., Plummer M., and Schiffman, M. Population-based study of human papillomavirus infection and cervical neoplasia in rural Costa Rica. J Natl Cancer Inst, *92:* 464-474, 2000.

Herrero, R., Schiffman, M. H., Bratti, C., Hildesheim, A., Balmaceda, I., Sherman, M. E., Greenberg, M., Cardenas, F., Gomez, V., Helgesen, K., Morales, J., Hutchinson, M., Mango, L., Alfaro, M., Potischman, N. W., Wacholder, S., Swanson, C., and Brinton, L. A. Design and methods of a population-based natural history study of cervical neoplasia in a rural province of Costa Rica: the Guanacaste project. Pan Am J Public Health, *1*: 362-375, 1997.

Hildesheim, A., Schiffman, M. H., Gravitt, P. E., Glass, A. G., Greer, C. E., Zhang, T., Scott, D. R., Rush, B. B., Lawler, P., Sherman, M. E., Kurman, R. J., and Manos, M. M. Persistence of type-specific human papillomavirus infection among cytologically normal women. J Infect Dis, *169*: 235-240, 1994.

Ho, G. Y. F., Burk, R. D., Klein, S., Kadish, A. S., Chang, C. J., Palan, P., Basu, J., Tachezy, R., Lewis, R., and Romney, S. Persistent genital human papillomavirus infection as a risk factor for persistent cervical dysplasia. J Natl Cancer Inst, *87*: 1365-1371, 1995.

IARC Working Group on the Evaluation of Carcinogenic Risks to Humans. Human Papilloma Viruses, vol. 64. Lyon, France: IARC, 1995.

Kirnbauer, R., Chandrachud, L. M., O'Neil, B. W., Wagner, E. R., Grindlay, G. J., Armstrong, A., McGarvie, G. M., Schiller, J. T., Lowy, D. R., and Campo, M. S. Virus-like particles of bovine papillomavirus type 4 in prophylactic and therapeutic immunization. Virology, *219*: 37-44, 1996.

Kirnbauer, R., Hubbert, N. L., Wheeler, C. M., Becker, T. M., Lowy, D. R., and Schiller, J. T. A virus-like particle enzyme-linked immunosorbent assay detects serum antibodies in a majority of women infected with human papillomavirus type 16. J Natl Cancer Inst, *86*: 494-499, 1994.

Koutsky, L. A., Ault, K. A., Wheeler, C. M., Brown, D. R., Barr, E., Alvarez, F. B., Chiacchierini, L. M., and Jansen, K. U. A controlled trial of a human papillomavirus type 16 vaccine. N Engl J Med, *347*: 1645-1651, 2002.

Koutsky, L. A., Holmes, K. K., Critchlow, C. W., Stevens, C. E., Paavonen, J., Beckmann, A. M., DeRouen, T. A., Galloway, D. A., Vernon, D., and Kiviat, N. B. A cohort study of the risk of cervical intraepithelial neoplasia grade 2 or 3 in relation to papillomavirus infection. N Engl J Med, *327(18)*: 1272-1278, 1992.

Kurman, R. J., Henson, D. E., Herbst, A. L., Noller, K. L., and Schiffman, M. H. Interim guidelines for management of abnormal cervical cytology. JAMA, *271*: 1866-1869, 1994.

Liaw, K. L., Hildesheim, A., Burk, R. D., Gravitt, P., Wacholder, S., Manos, M. M., Scott, D. R., Sherman, M. E., Kurman, R. J., Glass, A. G., Anderson, S. M., and Schiffman, M. A prospective study of human papillomavirus (HPV) type 16 DNA detection by polymerase chain reaction and its association with acquisition and persistence of other HPV types. J Infect Dis, *183*: 8-15, 2001.

Lowe, R. S., Brown, D. R., Bryan, J. T., Cook, J. C., George, H. A., Hofmann, K. J., Hurni, W. M., Joyce J. G., Lehman, E. D., Markus, H. Z., Neeper, M. P., Schultz, L. D., Shaw, A. R., and Jansen, K. U. Human papillomavirus type 11 (HPV-11) neutralizing antibodies in the serum and genital mucosal secretions of African green monkeys immunized with HPV-11 virus-like particles expressed in yeast. J Infect Dis, *176*: 1141-1145, 1997.

Munoz, N., Bosch, F. X., De Sanjose, S., Herrero, R., Castellsague, X., Shah, K. V., Snijders, P. J., Meijer, C. J.; International Agency for Research on Cancer Multicenter Cervical Cancer Study Group. Epidemiologic classification of human papillomavirus types associated with cervical cancer. N Engl J Med, *348*: 518-527, 2003.

Nardelli-Haefliger, D., Wirthner D, Schiller JT, Lowy, DR., Hildesheim, A., Ponci F, and De Grandi, P. Specific antibody levels at the cervix during the menstrual cycle of women vaccinated with human papillomavirus type 16 virus-like particles. J Natl Cancer Inst, *95*: 1128-1137, 2003.

Nobbenhuis, M. A. E., Walboomers, J. M. M., Helmerhorst, T. J. M., Rozendaal, L., Remmink, A. J., Risse, E. K. J., van der Linden, H. C., Voorhorst, F. J., Kenemans, P., and Meijer, C. J. L. Relation of human papillomavirus status to cervical lesions and consequences for cervical-cancer screening: a prospective study. The Lancet, *354*: 20-25, 1999.

Parkin, D. M., Pisani, P., and Ferlay, J. Global cancer statistics. CA- Ca J Clin, *49*: 33-64, 1999.

Reeves, W. C., Brinton, L. A., Garcia, M., Brenes, M. M., Herrero, R., Gaitan, E., Tenorio, F., De Britton, R. C., and Rawls, W. E. Human papillomavirus infection and cervical cancer in Latin America. N Engl J Med, *320*: 1437-1441, 1989.

Schiffman, M. H., Bauer, H. M., Hoover, R. N., Glass, A. G., Cadell, D. M., Rush, B. B., Scott, D. R., Sherman, M. E., Kurman, R. J., Wacholder, S., Stanton, C. K., and Manos, M. M. Epidemiologic evidence showing that human papillomavirus infection causes most cervical intraepithelial neoplasia. J Natl Cancer Inst, *85*: 958-964, 1993.

Schiffman, M. H., Liaw, K. L., Herrero, R., Sherman, M. E., and Hildesheim, A. Epidemiologic Support for a Simplified View of Cervical Carcinogenesis. Genital Infections and Neoplasia, *1*: 2-6, 1998.

Schiffman, M., Herrero, R., Hildesheim, A., Sherman, M. E., Bratti, M. C., Wacholder, S., Alfaro, M., Hutchinson, M., Morales, J., Greenberg, M. D., and Lorincz, A. T. HPV DNA testing in cervical cancer screening: results from women in a high-risk province of Costa Rica. JAMA, *283*: 87-93, 1999.

Solomon, D., Davey, D., Kurman, R., Moriarty, A., O’Connor, D., Prey, M., Raab, S., Sherman, M., Wilbur, D., Wright, Jr., T., Young, N., for the Forum Group Members and the Bethesda 2001 Workshop. The 2001 Bethesda System – Terminology for reporting results of cervical cytology. JAMA, *287*: 2114-2119, 2002.

Solomon, D., Schiffman, M., Tarone, R.; ALTS Study Group. Comparison of three management strategies for patients with atypical squamous cells of undetermined significance: baseline results from a randomized trial. J Natl Cancer Inst, *21*: 293-299, 2001.

Suzich, J. A., Ghim, S. J., Palmer-Hill, F. J., White, W. I., Tamura, J. K., Bell, J. A., Newsome, J. A., Jenson, A. B., and Schlegel, R. Systemic immunization with

papillomavirus L1 protein completely prevents the development of viral mucosal papillomas. Proc Natl Acad Sci, *92*: 11553-11557, 1995.

Taylor M. L., Garcia Z., Holst I., Somogye T., Cunningham L., and Visona K. A. Seroprevalencia de los virus de la hepatitis A y B en grupos etarios de Costa Rica. Acta medica costarricense, *43*: 153-158, 2001.

The Atypical Squamous Cells of Undetermined Significance/Low-Grade Squamous Intraepithelial Lesions Triage Study (ALTS) Group. Human papillomavirus testing for triage of women with cytologic evidence of low-grade squamous intraepithelial lesions: baseline data from a randomized trial. J Natl Cancer Inst, *92:* 397-402, 2000.

Woodman, C. B., Collins, S., Winter, H., Bailey, A., Ellis, J., Prior, P., Yates, M., Rollason, T. P., Young, L. S. Natural history of cervical human papillomavirus infection in young women: a longitudinal cohort study. Lancet, *357*: 1831-1836, 2001.

11. APPENDICES

Appendix A CLINICAL MANAGEMENT ALGORITHMS

Colposcopy Referral Algorithm

Referral Algorithm

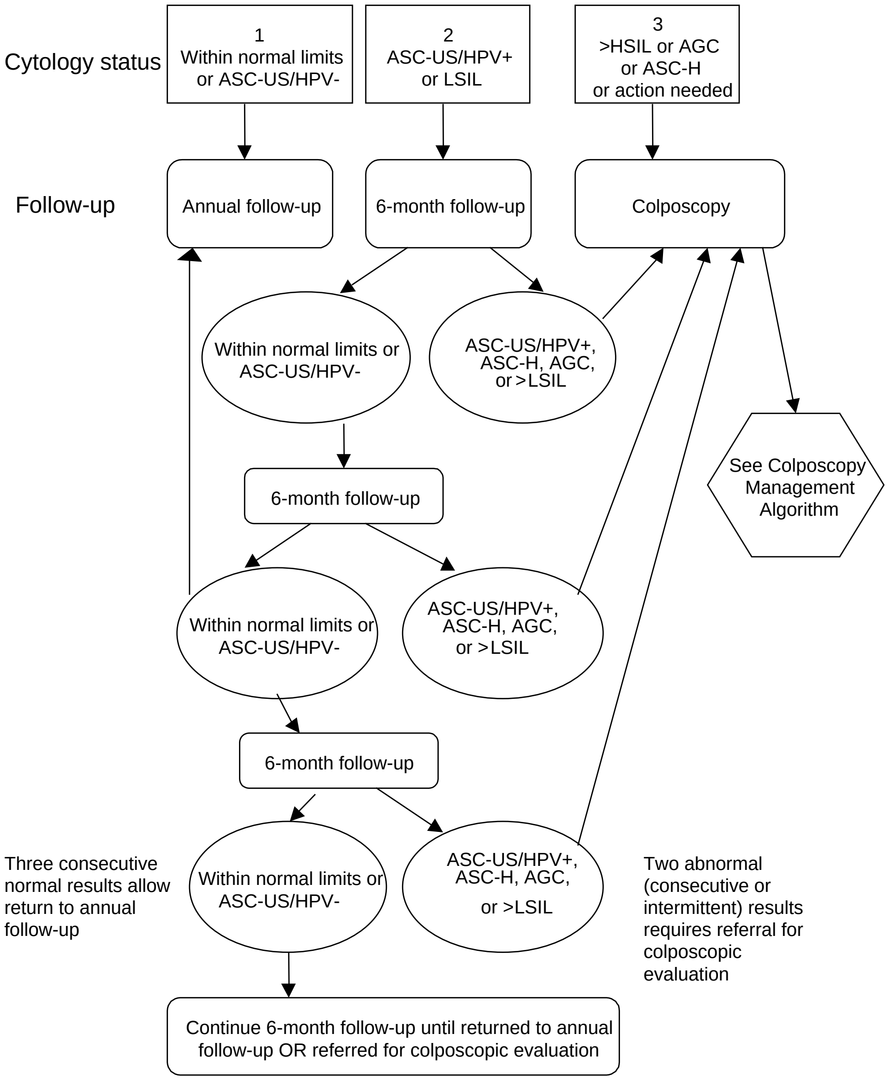

Colposcopy Management Algorithm

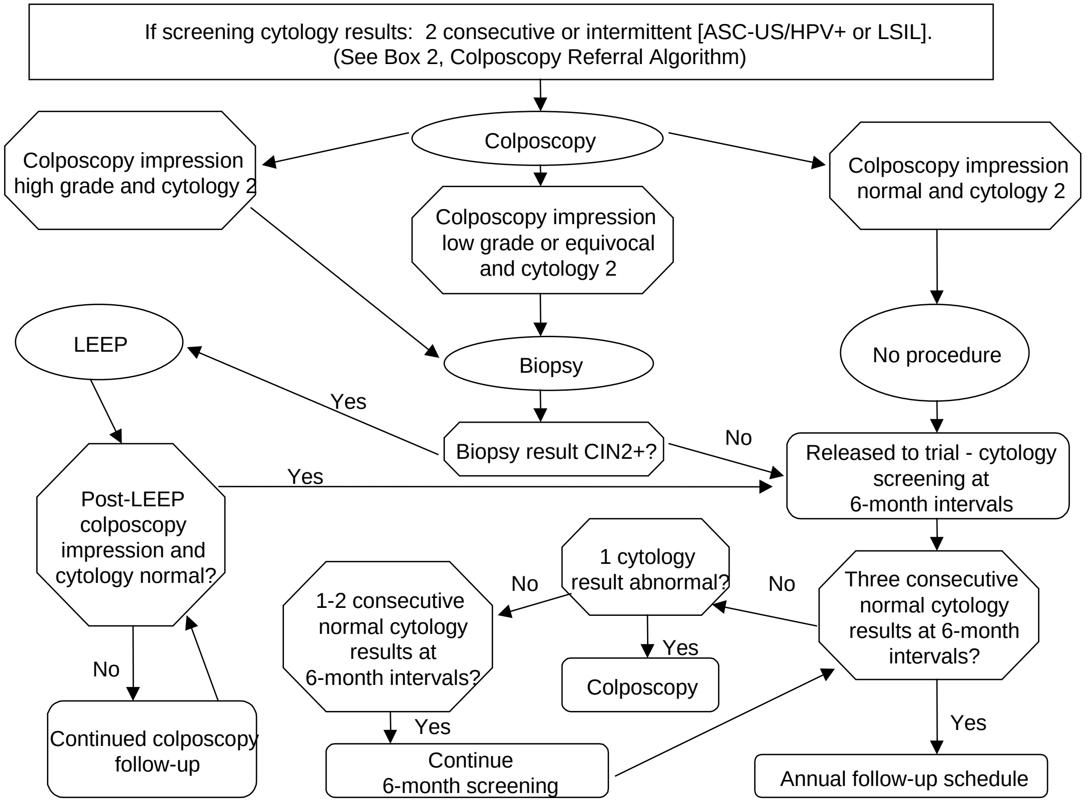

Colposcopy Management Algorithm

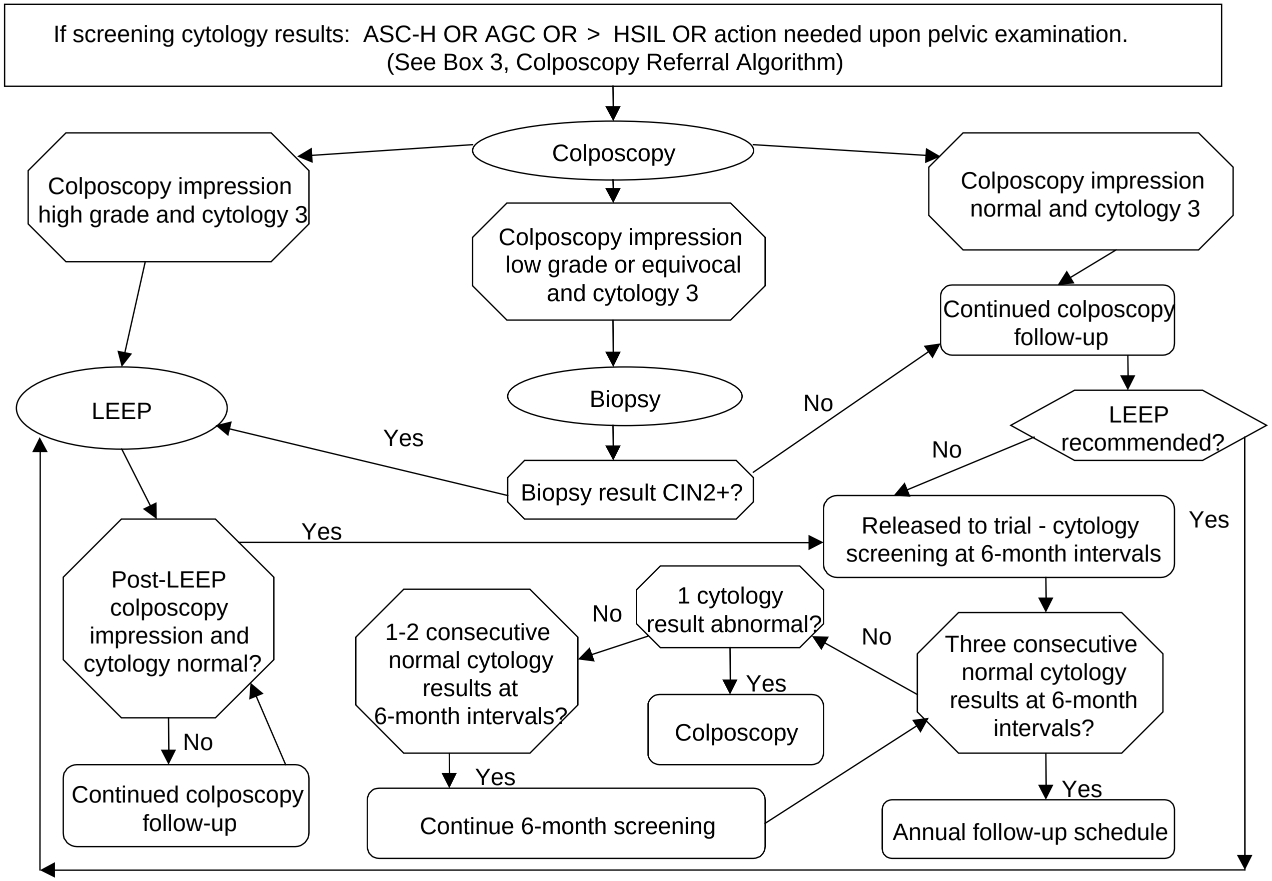

Colposcopic Treatment Table

Referral Reason	Colposcopic Impression			
	High-Grade	Low-Grade or equivocal	Negative	Unsatisfactory*
ASC-H, or AGC, or HSIL+ or action needed	LEEP	Biopsy	Colpo 6 months	Colpo within 6 months**
2 consecutive or intermittent [ASC-US/HPV+ or LSIL]	Biopsy	Biopsy	Colpo 6 months	Colpo within 6 months

*Unsatisfactory means no lesion was seen in ectocervix but a portion of the endocervical canal was not visible. If unsatisfactory for other reasons, reschedule colposcopy visit.
**If the reason for the unsatisfactory colposcopy can be modified (e.g., heavy discharge, etc) the woman will be appointed for a new colposcopy as soon as possible. If the reason can not be modified (TZ inside the canal with narrow cervical os) the participant will be given a new appointment to colposcopy (including cytology with a Cytobrush) within 2 months. If the high-grade lesion is confirmed LEEP will be performed. If the second test is negative, a third colposcopy will be done six months later.

Amended: 13 Nov 2008

Colposcopy Referral Algorithm- Month 48 Visit

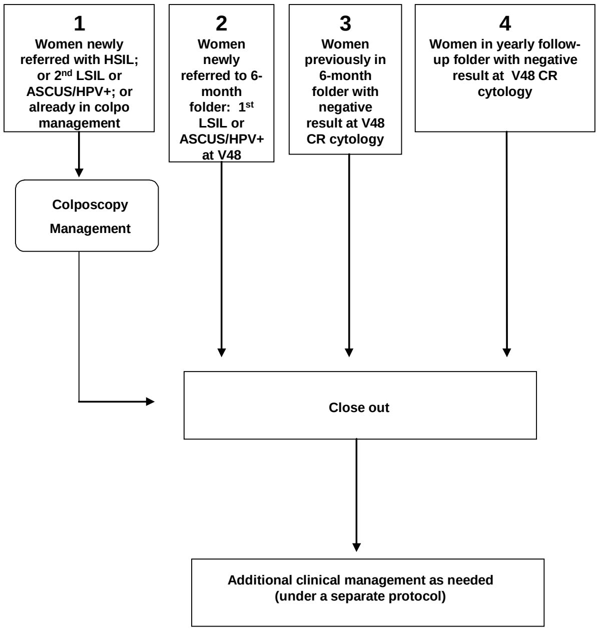

Appendix B RANDOMIZATION PROCEDURES

Approach to Labeling and Distribution of Randomized Vaccine and Control Syringes

Women identified in the enumeration survey ages 18 to 25 at the time of enrollment will be invited to participate in the trial. Enrollment will occur at study clinics in the Guanacaste province. Subjects will **not** be randomly assigned to a particular arm (i.e., material type) prior to study start. Instead, randomization to one of the two trial arms will occur when the subject receives her first vaccination. The procedure for vaccine labeling and distribution is described below.

Vaccine and control syringes will be appropriately labeled using a randomized number list associated with the vaccine and the control syringes. Vaccine identification numbers will be randomized to HPV16/18 VLP and control vaccine syringes by NCI using a standard SAS (Statistical Analysis System) program. The vaccine identification numbers will uniquely identify the vaccine doses to be administered to the same subject. The randomization listing will be used by the NCI to generate vaccine labels. Labels containing the randomized numbers will be provided to GSK Biologicals in Rixensart, where they will be applied to the respective syringes. Labeled HPV16/18 VLP vaccine and control syringes will be combined, sorted in numerical order, and delivered in sequentially numbered boxes to the study site in Costa Rica. Syringes will be pulled in numeric order for vaccination in the study clinics from these boxes.

In preparation for vaccine distribution to the clinics at the enrollment visit, first dose vaccine syringes will be pulled in numeric order and placed in boxes for transport to each clinic according to the number of subjects appointed to each clinic. The vaccine syringes to be used in administration at enrollment are already labeled with a label containing the sample vaccine Identification number (vaccine ID) and the sequence number representing the dose (e.g., VX 21234-001) of the vaccine being administered. As an example, for an enrollment visit at Clinic 1 where 25 subjects have been invited, vaccines starting with a vaccine ID of VX12345-001 and continuing through VX12369-001 will be placed in a transport box in a serpentine fashion by ascending number. Boxes filled with syringes for Clinic 2 will be filled with syringes VX12370-001 to VX12394-001 for 25 potential subjects, and so on. As a subject is determined to be eligible, the next available syringe with the lowest value vaccine ID number will be pulled for application. In this manner, as subjects randomly enter the study, the integrity of randomization carried out during the numbering and labeling process is maintained and study site personnel and subjects are blinded to material type.

However, it should be noted that while this **pattern** of placing syringes by ascending numeric order will be followed for distribution of vaccine and control syringes, the syringes may not be sequentially ordered after the first day of the trial. This is due to the fact that not all invited subjects will become eligible to participate in the trial, and some first dose syringes in each batch distributed to a clinic may be left unused. Unused syringes will be returned to inventory on a daily basis. Upon assignment of subjects to the next day’s clinics, the next available first dose syringe with the lowest vaccine ID will be used until a sufficient number of syringes are packed for distribution at each clinic. Due to this process, the vaccine IDs in each batch of syringes sent to a clinic could have some

numeric gaps between syringe identification numbers. However, the pattern of placing the lowest vaccine ID followed by ascending vaccine IDs will continue, thereby ensuring the assignment of a syringe in an ordered fashion, thus preserving randomization.

As the trial progresses, the second and third doses of vaccine and control syringes will be needed. The syringes for the second and third doses will be selected based on linkage of the vaccine ID to the first dose so as to maintain the same material type for each participant. A subject who received a vaccination from a syringe with a vaccine ID label of VX12345-001, would receive her second and third doses from syringes with a vaccine ID of VX12345-002 and VX12345-003, respectively. The second and third doses of the vaccine and control syringes will also be distributed in each clinic in ascending numeric order. This method of placement in transport boxes and use in the field allows easy accessibility for study personnel. Moreover, this procedure provides for the initial randomization of material type to a subject and the assurance that the second and third dose for each subject is the same material type as the first dose received.

In the event that an assigned syringe (i.e., a dose 2 or dose 3 syringe) is determined to be unusable, additional doses will be available on site to be used as replacements. In case an assigned syringe dose is broken or unusable, the investigator will replace it with a replacement syringe, using a web-based system developed by trial Data Management Center (DMC), under contract with the NCI. This system will ensure a fast, secure, and masked process for assigning replacement syringes. Only authorized personnel with valid logon and password information will be allowed to access the web-based replacement system; no treatment information will be accessible via this system. The replacement process will be documented on the vaccine administration page of the CRF and on the web-based replacement application.

Formatted: Danish

Formatted: Danish

Appendix C HPV PCR TESTING ALGORITHM FOR HPV16 & HPV18 DETERMINATION

Proposed Phase III Algorithm

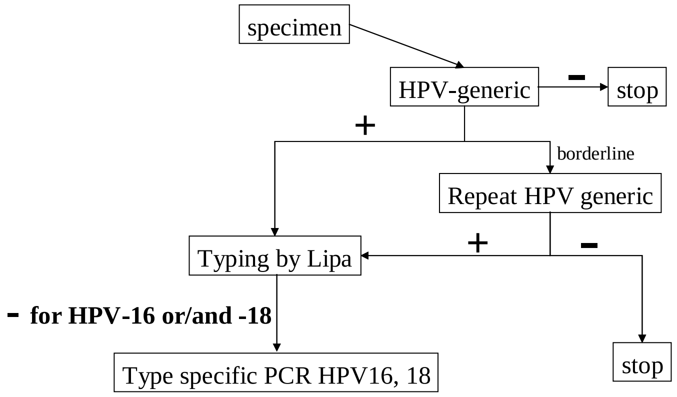

Appendix D CRITERIA FOR DEFINING ADVERSE EVENTS (AEs), SERIOUS AEs (SAEs), AND PROCEDURES FOR AND TIMING OF AE REPORTING

Adverse Events and Serious Adverse Events

The investigator is responsible for the detection and documentation of events meeting the criteria and definition of an adverse event (AE) or serious adverse event (SAE) as provided in this Appendix. During the study, when there is a safety evaluation, the investigator will be responsible for detecting AEs and SAEs, as detailed in this Appendix.

Each subject will be instructed to contact the investigator immediately should she manifest any signs or symptoms indicated below.

1. Definition of an adverse event

An AE is any untoward medical occurrence in a clinical investigation subject, temporally associated with the use of a medicinal product, whether or not considered related to the medicinal product.

An AE can therefore be any unfavorable and unintended sign (including an abnormal laboratory finding), symptom, or disease (new or exacerbated) temporally associated with the use of a medicinal product.

Examples of an AE include:

- Exacerbation of a chronic or intermittent pre-existing condition including either an increase in frequency and/or intensity of the condition.
- New conditions detected or diagnosed after investigational product administration even though it may have been present prior to the start of the study.
- Signs, symptoms, or the clinical sequelae of a suspected interaction.
- Signs, symptoms temporally associated with vaccine administration.

Examples of an AE DO NOT include:

- Medical or surgical procedure (e.g., endoscopy, appendectomy); however, the condition that leads to the procedure is an AE.
- Situations where an untoward medical occurrence did not occur (social and/or convenience admission to a hospital).
- Anticipated day-to-day fluctuations of pre-existing disease(s) or condition(s) present or detected at the start of the study that do not worsen.

AEs may include pre- or post-treatment events that occur as a result of protocol-mandated procedures (i.e., invasive procedures, modification of subject's previous therapeutic regimen).

NOTE: Pre-existing conditions or signs and/or symptoms present in a subject prior to the start of the study (i.e., prior to the first study procedure) should be recorded in the Enrollment General Physical Exam and Medical History form CE_MH.

2. Definition of a serious adverse event

A serious adverse event (SAE) is any untoward medical occurrence that:

a. Results in death,

b. Is life-threatening

NOTE: The term 'life-threatening' in the definition of 'serious' refers to an event in which, in the opinion of the investigator, the subject is at risk of death at the time of the event as it occurred; i.e., it does not include an event that, had it occurred in a more severe form, may have caused death (21 CFR 312.32).

c. Requires hospitalization or prolongation of existing hospitalization

NOTE: In general, hospitalization signifies that the subject has been detained (usually involving at least an overnight stay) at the hospital or emergency ward for observation and/or treatment that would not have been appropriate in the physician’s office or outpatient setting. Complications that occur during hospitalization are AEs. If a complication prolongs hospitalization or fulfils any other serious criteria, the event is serious. When in doubt as to whether “hospitalization” occurred or was necessary, the AE should be considered serious.

Hospitalization for elective surgery related to a pre-existing condition that did not worsen from baseline is not considered an AE.

d. Results in persistent or significant disability/incapacity,

e. Is a congenital anomaly/birth defect in the offspring of a study subject,

NOTE: Medical or scientific judgement should be exercised in deciding whether reporting is appropriate in other situations, such as important medical events that may not be immediately life-threatening or result in death or hospitalization but may jeopardize the subject or may require medical or surgical intervention to prevent one of the other outcomes listed in the above definition. These should be considered serious. Examples of such events are invasive or malignant cancers, intensive treatments in an emergency room or at home for allergic bronchospasm, blood dyscrasia, or convulsion but do not result in hospitalization.

3. Lack of efficacy

“Lack of efficacy” *per se* will not be reported as an AE. The signs and symptoms or clinical sequelae resulting from lack of efficacy will be reported if they fulfil the AE or SAE definition.

4. Clinical laboratory parameters and other abnormal assessments qualifying as adverse events and serious adverse events

Abnormal laboratory findings (e.g., clinical chemistry, cytology) or other abnormal assessments (e.g., vital signs) that are judged by the investigator to be clinically significant will be reported as AEs or SAEs if they meet the definition of an AE, as defined in Section 1, or SAE, as defined in Section 2. Clinically significant abnormal laboratory findings or other abnormal assessments that are detected during the study or are present at baseline and significantly worsen following the start of the study will be reported as AEs or SAEs. The investigator will exercise his or her medical and scientific judgement in deciding whether or not an abnormal laboratory finding or other abnormal assessment is clinically significant.

5. Time period, frequency, and method of detecting adverse events and serious adverse events

The standard time period for collecting and recording SAEs will begin after receipt of the participant's informed consent signature and will end at the concluding visit, which will occur at approximately 48 months following administration of the first dose of study vaccine for each subject. (Amended: 29 Mar 2005) See Section 8 for instructions for reporting and recording SAEs.

Additionally, in order to fulfill international reporting obligations, SAEs that are related to study participation (e.g., procedures, invasive tests, a change from existing therapy) or are related to a concurrent medication will be collected and recorded from the time of consent until the subject completes her concluding visit or withdraws from the study (e.g., early withdrawal from the study, loss to follow-up). (Amended: 29 Mar 2005)

The investigator will record all AEs during the entire active phase (including the approximately 48 month follow-up after consent (Amended: 29 Mar 2005), SAEs during the entire study period, and the onset of chronic illness(es) (diabetes, autoimmune disease, asthma, and allergies), emergency room (ER) visits and physician office visits that are not related to routine physical examination, vaccination, or common acute illnesses such as upper respiratory infection, sinusitis, pharyngitis, gastroenteritis and injury.

The AER and any other relevant forms (e.g., VAR, HV2) (Amended: 29 Mar 2005) will be completed for every report that meets the definition of an adverse event. The investigator will attempt detection of AEs and SAEs in the following ways: For all subjects the investigator will monitor and record the occurrence of reactions in the first 60 (Amended: 31 Oct 2005) minutes following administration of the vaccine at every vaccination visit (baseline [0 month], 1 month and 6 months) using the Vaccine Administration and Reactogenicity Monitoring (VAR) form. In addition, 10% of women will receive home visits from outreach workers 3 to 6 days post vaccination to monitor AEs (vaccination day is defined as day zero for this purpose). The Home Visit Reactogenicity Monitoring (HV2) form will be used for these visits. Spontaneous reports of solicited adverse events that are reported by phone to the physician on-call or in-person during a vaccination visit at the clinic will be documented on the AER (Amended: 29 Mar 2005) form. In addition, the AER form will be completed by fieldworkers during home visit to participants that had unsolicited AEs or SAEs during a vaccination clinic visit or other adverse event for which the physician orders follow-up to be conducted. Adverse events will also be monitored on the Post-Enrollment Eligibility Screener (PES)

form which is administered at the one month and six month vaccination visits and at follow-up visits occurring at 12, 18, 24, 30, 36, 42, and 48 months after the first vaccination visit and on the Colposcopy Intake Form (CIF) for women who are referred for colposcopic evaluation. Another surveillance effort will be periodic query to identify study subjects on summary lists of women in the same age range who were admitted to hospitals or ER clinics in Guanacaste and selected referral hospitals outside of Guanacaste. The investigator will evaluate all AEs observed by the investigator or one of his/her clinical collaborators or reported by the subject, whether spontaneously, or in response to a direct question. AEs not previously documented in the study will be recorded on the AER form. The nature of each event, date and time (where appropriate) of onset, outcome, resolution date, intensity and relationship to vaccination will be established.

When an AE/SAE occurs, it is the responsibility of the investigator to review all documentation (e.g., hospital progress notes, laboratory, and diagnostics reports) relative to the event. The investigator will then record all relevant information regarding an AE/SAE on the AER form. There may be instances when the NCI Medical Monitor will request additional abstraction of information from the participant's medical records. GlaxoSmithKline (GSK) Biologicals, hereafter referred to as the IND holder, may also request additional information, but will do so via the NCI Medical Monitor or NCI Project Officer (Amended: 31 Oct 2005). In these instances, all subject identifiers will be removed by the investigator prior to document transmission.

The investigator will attempt to establish a diagnosis of the event based on signs, symptoms, and/or other clinical information. In such cases, the diagnosis should be documented as the AE/SAE and not the individual signs/symptoms.

5.1 Solicited adverse events

For all subjects the investigator will monitor and record the occurrence of reactions in the first 60 (Amended: 31 Oct 2005) minutes following administration of the vaccine at every vaccination visit (baseline [0 month], 1 month and 6 months) using the VAR form. In addition, a random sampling of 10% of the women enrolled in the trial will receive home visits from outreach workers 3 to 6 days after each vaccination to monitor for AEs (vaccination day is defined as day zero). The same randomly selected 10% sample of women will receive home visits after each vaccination. The HV2 form will be used during these 3-6 day post vaccination home visits. With the exception of this sample of 10% of participants who are scheduled to receive a home visit, the AER (Amended: 29 Mar 2005) form will be used to follow up on all other AEs that occur in days 0-6 after vaccination. For example, the AER (Amended: 29 Mar 2005) form will be used to follow up on spontaneously reported AEs as well as AEs that are identified during physician directed home visits.

5.1.1 Solicited local (injection site) AEs

Standardized data will be collected for local injection site AEs on the VAR and HV2 (Amended: 29 Mar 2005) for pain, redness, swelling, and temperature at the injection site. Subjects will be asked, "Do you feel pain at the injection site"? Those who answer "Yes", will be asked to rate the intensity of the pain at the injection site. Possible

responses: 1 (Mild), 2 (Moderate), 3 (Severe). (Refer to Section 6 for intensity definitions.)

The following observations will also be made of the injection site:

Area of redness __ __ mm   Area of swelling __ __ mm

Temperature at injection site ("Normal" or "Warmer than surrounding area")

5.1.2 Other solicited general AEs

5.1.2.1 Sixty-minute (Amended: 31 Oct 2005) post-vaccination monitoring

Pre-vaccination oral temperature and 60-minute (Amended: 31 Oct 2005) post-vaccination oral temperature are recorded on the VAR form. Standardized data are collected on the VAR form to document the following general solicited AEs: fatigue, myalgia, arthralgia, GI symptoms (nausea, vomiting, diarrhea, and/or abdominal pain), headache, rash (other than urticaria), and urticaria. Subjects reporting any of these solicited symptoms will be asked to rate the intensity of each symptom (mild, moderate, severe). If a solicited general AE meets the criteria of an SAE this is indicated on the form and an AER form is completed.

5.1.2.2 Home visit reactogenicity monitoring

Oral temperature is recorded on the HV2 form and any temperature above 39°C is documented on the AER form and followed to resolution. Fever can be classified as an SAE at the discretion of the physician. Subjects providing responses to home visit interviews will be asked whether they experienced the following solicited symptoms since their most recent vaccination: fatigue, pain in their muscles, aches or pain in their joints, GI symptoms (nausea, vomiting, diarrhea, and/or abdominal pain), headache, rash (other than urticaria), and urticaria. Subjects reporting any of these solicited symptoms will be asked to rate the maximum intensity of each symptom (mild, moderate, severe). If a solicited general AE meets the criteria of an SAE, this is indicated on the HV2 form and an AER form is completed.

Possible responses to each solicited symptom: 1 (Yes), 2 (No)

Next they will be asked, "Do you still have this adverse event?"

Possible responses: 1 (Yes), 2 (No)

They will be asked about the maximum intensity of the event: 1=Mild, 2=Moderate, 3=Severe.

Participants will also be asked, "How long have you had/did you have this condition?" 0 (<1 day), 1 (1-2 days), 2 (≥3 days)

5.1.2.3 Spontaneous reports of solicited AEs

Spontaneous reports of AEs will be captured directly on the AER form. (Amended: 29 Mar 2005)

5.2 Unsolicited AEs

The VAR *and* HV2 forms have fields for the collection of information on unsolicited conditions that are observed or reported after vaccination (VAR form) or at the 3-6 day home visit following vaccination for 10% of the women (HV2 form). When completing these forms, if any unsolicited AEs are reported, the person completing the form is instructed to complete an AER form. Also, unsolicited AEs reported outside the context of a clinic visit or a home visit on Days 3-6 after vaccination for 10% of the women will be documented directly on the AER form. (Amended: 29 Mar 2005)

The Post Enrollment Eligibility Screener form (PES) is used at all scheduled post-enrollment clinic appointments. The Colposcopy Intake Form (CIF) is used at the intake visit to the colposcopy clinic. The PES and CIF forms contain essentially identical questions about any hospitalizations, doctor visits, or illnesses lasting more than one day that have occurred since the last study visit. An AER form is to be completed for any such occurrences.

6. Evaluating adverse events and serious adverse events

6.1 Assessment of intensity

The site investigator or designee(s) will be responsible for direct measurements related to several solicited AEs: oral temperature (measured in Celsius), the area of redness and area of swelling at the injection site (measured in millimeters), and temperature at the injection site.

6.1.1 Temperature at injection site

(0) Normal: The injection site is no warmer than the surrounding area.

(1) Warmer than the surrounding area

6.1.2 Other signs and symptoms

The area of local injection site redness/swelling will each be measured in millimeters and recorded on the relevant forms (i.e., the VAR or HV2 form). (Amended: 29 Mar 2005)

The maximum intensity of local injection site redness/swelling will be scored for purposes of analysis as follows:

(0) None

(1) >0 mm to ≤20 mm

(2) >20 mm to ≤50 mm

(3) >50 mm

In addition, the intensity of symptoms will be solicited for the following AEs: pain at the injection site, fatigue, myalgia, arthralgia, GI symptoms (nausea, vomiting, diarrhea and/or abdominal pain), headache, rash (other than urticaria), and urticaria. The following severity/intensity scale will be used for this purpose:

(0) None

(1) (Mild) = An AE which is easily tolerated by the subject, causing minimal discomfort and not interfering with everyday activities.

(2) (Moderate) = An AE which is sufficiently discomforting to interfere with normal everyday activities.

(3) (Severe)=An AE which prevents normal, everyday activities. (In adults/ adolescents, such an AE would, for example, prevent attendance at work/school and would necessitate the administration of corrective therapy.)

For urticaria, maximum intensity of the event will be classified as:

(1) Urticaria occurring on 1 body area only.

(2) Urticaria occurring on 2 or 3 body areas.

(3) Urticaria occurring on at least 4 body areas.

NOTE: An AE that is assessed as severe should not be confused with a Serious Adverse Event. “Severe” is a category utilized for rating the intensity of an event, and both AEs and SAEs can be assessed as “severe”. An event is defined as “serious” when it meets one of the pre-defined outcomes as described in Section 2 above (e.g., adverse events that are life-threatening, fatal, or result in prolonged hospitalization, persistent or significant disability/incapacity, or congenital anomaly).

6.1.3 Fever

Fever will be recorded on the VAR or HV2 (Amended: 29 Mar 2005) form, as appropriate, and will be categorized into severity/intensity categories for analysis purposes according to the following oral temperature cut-offs:

(0) <37.5 °C

(1) ≥37.5 °C to <38.0 °C

(2) ≥38.0 °C to 39.0 °C

(3) >39.0 °C

Fever will be classified as a serious adverse event based on the medical and scientific judgement of the site investigator or designee(s).

6.2 Assessment of causality

The investigator is obligated to assess the relationship between investigational product and the occurrence of each AE/SAE. The investigator will use clinical judgement to determine the relationship. In doing so, alternative causes, such as the natural history of the underlying diseases, concomitant therapy, other risk factors and the temporal relationship of the event to the investigational product will be considered. The investigator will provide this information by completing Part B of the AER form (section to be completed by the physician regarding diagnosis, relationship, additional information, etc.).

The relationship of the adverse event to the vaccination will be coded as follows:

(0) None

(1) Possibly related

(2) Probably related

(3) Definitely related

The code and signature of the physician, as well as the date that Part B was completed by the physician will be entered on the AER form.

There may be situations when an SAE has occurred and the investigator has minimal information to include in the initial report to the Westat Regulatory Associate; however, it is important that the investigator make an initial assessment of causality for an event prior to transmission of the AER form.

7. Follow-up of adverse events and serious adverse events and assessment of outcome

After the initial AER form is completed, the investigator will follow up and provide information on the subject’s condition. The AER form (question 5) identifies the report as either a new event or a follow-up to a previous adverse event.

All AEs and SAEs documented at a previous visit/contact and designated as unresolved will be reviewed at subsequent visits/contacts.

Investigators will follow up subjects:

- with SAEs, until the event has resolved, subsided, stabilized, or disappeared; the event is otherwise explained; or the subject is lost to follow-up;
- or, in the case of other non-serious AEs, until the event has resolved, subsided, stabilized, or disappeared; the subject completes the study; or the subject is lost to follow-up; whichever occurs first.

Clinically significant laboratory abnormalities will be followed up until they have returned to baseline, or a satisfactory explanation has been provided. Reports relative to the subsequent course of an SAE noted for any subject must be submitted to the Westat Regulatory Associate.

The NCI Medical Monitor or IND holder, via the NCI Medical Monitor or NCI Project Officer (Amended: 31 Oct 2005), may request that the investigator obtain additional information from the participant's clinical records to elucidate as fully as possible the nature and/or causality of the AE or SAE. The investigator is obliged to assist. If a subject dies during participation in the study or during a recognized follow-up period, the investigator will obtain copies of any available post-mortem findings, including histopathology, and will forward them to Westat's Regulatory Associate for appropriate distribution.

Updated information will be recorded on new AER forms linked to the original form with an AE number provided by the study data management system. The updated SAE report form should be resent to the Westat Regulatory Associate within 24 hours of receipt of the follow-up information as outlined in Section 8. (Amended: 13 Nov 2008)

Outcome of any non-serious AE (i.e., unsolicited AE) or any SAE reported during the entire study will be assessed as indicated by AER form question 8 (Resolution Code).

01 = Recovered/resolved without sequelae and without intervention

02 = Recovered/resolved without sequelae and with intervention

03 = Recovered/resolved with sequelae

04 = Unresolved

05 = Fatal/Died

06 = Other, specify

8. Prompt reporting of serious adverse events

8.1 Time-frame for submitting serious adverse event reports to Westat

SAEs will be reported promptly to the Westat Regulatory Associate once the investigator determines that the event meets the protocol definition of an SAE (Figure 3). In the event of a life-threatening or fatal SAE, the investigator or designate will telephone the Westat

Regulatory Associate to inform him/her about the SAE and also fax the completed AER form and supporting documentation to the Westat HPV Regulatory Associate WITHIN 24 HOURS OF HIS/HER BECOMING AWARE OF THESE EVENTS. **All other SAEs, regardless of relationship, will be reported to the Westat Regulatory Associate within three calendar days of the initial report via email (notification only) and fax (AER form and supporting documentation).** The Westat Regulatory Associate will forward these expedited SAE reports to the NCI Medical Monitor and IND holder within one business day of receipt from Costa Rica, for their review and assessment. Additional or follow-up information relating to the initial SAE report is also reported to Westat within 24 hours of investigator receipt and forwarded from Westat to the NCI Medical Monitor and IND holder within one business day of receipt of such additional information from Costa Rica.

8.2 Completion and transmission of serious adverse event reports to Westat

When an investigator becomes aware of an SAE for a study subject, she/he will report the information to Westat's Regulatory Associate who is responsible for coordinating receipt and distribution of SAE reports to the NCI Medical Monitor and IND holder within the time-frames outlined in Section 8.1 and Figure 3. The AER form will always be completed with all available details of the event, signed by the investigator (or designee), and forwarded to Westat within the designated time-frame. If the investigator does not have all information regarding an SAE, he/she will not wait to receive additional information before notifying the Westat Regulatory Associate of the event by faxing the completed form. The form will be updated when additional information is received and forwarded to Westat as outlined in Section 8.1. The investigator will provide an assessment of causality at the time of the initial report as described in Section 6.2.

Facsimile (fax) transmission of the AER Report Form is the preferred method to transmit SAE information to the Westat Regulatory Associate. In rare circumstances (e.g., the absence of facsimile equipment), notification by telephone is acceptable, with a copy of the AER form sent by overnight mail. Initial notification via the telephone does not replace the need for the investigator to complete, sign, and transmit the AER form as outlined in Section 8.1.

Westat Regulatory Associate for Reporting SAEs
Mujadala Abdul-Majid, Regulatory Affairs Assistant (Amended: 13 Nov 2008)
Health Studies Area, Westat, Inc.
1441 West Montgomery Ave., Westbrook, WB 335
Rockville, MD 20850
Tel: 301-738-3642 (direct)
Urgent phone: 240-453-5661
Fax: 240-314-2547
Email: mujadala@westat.com

Back-up Westat Regulatory Associate for Reporting SAEs
Meredith Melick (Amended: 31 Oct 2005)
Westat, Inc.
1650 Research Blvd., WB 352
Rockville, MD 20850
Tel: 240-453-2688 (Amended: 31 Oct 2005) (direct)
Urgent Phone: 240-453-5661
Fax: 240-314-2547
Email: meredithmelick@westat.com (Amended: 31 Oct 2005)

NCI Medical Monitor for Reporting SAEs
Mark Schiffman, MD
6120 Executive Boulevard MSC #7234
Rockville, MD 20852
Tel: 301-435-3983
Fax: 301-402-0916
Outside office hours
Tel: 301-279-7040
Fax: 301-402-0916

Back-up NCI Medical Monitor for Reporting SAEs
Diane Solomon, MD
Division of Cancer Prevention, BGCRG
6130 Executive Boulevard, Room 2123
Rockville, MD 20852-7333
Tel: 301-402-6211
Fax: 301-480-9939
Outside office hours
Tel: 301-343-5585
Fax: 301-229-8309

GSK Global Clinical Safety and Pharmacovigilance (GCSP) Email and Fax Addresses for Reporting SAEs and Pregnancy Information
Fax: 919-483-5404
Email: us.naps@gsk.com

For general safety inquiries: (Amended: 29 Mar 2005)
Desma Altobelli, PharmD
Principal Clinical Safety Scientist
Global Clinical Safety and Pharmacovigilance
Tel: 919-483-5176
Fax: 919-483-5404
E-mail: desma.j.altobelli@gsk.com
GSK Back-up Study Contact for Reporting SAEs
GSK Biologicals Clinical Safety Physician
Tel: +32-2-656 87 98
Fax: +32-2-656 80 09
Mobile phone for 7/7 day availability: +32 477 404 713
24/24 hour and 7/7 day availability

9. Regulatory reporting requirements for serious adverse events

The investigator promptly reports SAEs to Westat's Regulatory Associate, who forwards them to NCI's Medical Monitor and the IND holder as outlined in Section 8. The holder of the IND is responsible for notifying the Food and Drug Administration (FDA) and other regulatory bodies about the safety of a product under clinical investigation. Prompt reporting of an SAE to the IND holder is essential to meet legal and ethical responsibilities for the safety of all subjects. The IND holder reports SAEs to FDA following these timelines:

EVENT CATEGORY	REPORTING TIMELINES
Non life-threatening	Within 15 calendar days from the day the sponsor first received knowledge of the event
Life-threatening or Fatal	Notify FDA by phone or fax within 7 calendar days from the day the sponsor first received knowledge of the event, followed by as complete a report as possible within 8 additional calendar days

The investigator, or responsible person according to local requirements, will comply with the applicable local regulatory requirements related to the reporting of SAEs to local regulatory authorities and the IRB/IEC.

This protocol has been filed under an Investigational New Drug (IND) application with the US Food and Drug Administration (FDA). A given SAE may qualify as an IND Safety Report (INDSR) if the SAE is both attributable to the investigational product and unexpected. In this case, all investigators conducting studies under the IND (and associated INDs for the same compound) will receive an INDSR (previously referred to as Expedited Investigator Safety Report or EISR. (Amended: 29 Mar 2005).

INDSRs are prepared according to the IND holder's policy and are forwarded by the IND holder to the Westat Regulatory Associate for distribution to NCI and Costa Rica investigators. The purpose of the INDSR is to fulfil specific regulatory and Good Clinical Practice (GCP) requirements regarding the product under investigation. When the NCI and Costa Rica investigators receive an initial or follow-up INDSR or other safety information (e.g., revised Investigator Brochure) from the Westat Regulatory Associate, it will be their responsibility to notify the DSMB and the local and NCI IRBs. (Amended: 29 Mar 2005; EISR revised to INDSR throughout paragraph)

10. Post study adverse events and serious adverse events

A post-study AE/SAE is defined as any event that occurs outside of the AE/SAE detection period defined in Section 5. Investigators are not obligated to actively seek AEs or SAEs in former study participants. If the investigator learns of any SAE, including a death, at any time after a subject has been discharged from the study, and considers the event reasonably related to the investigational product, the investigator will notify the sponsor. (Amended: 13 Nov 2008)

11. Pregnancy

Urine pregnancy tests are performed at the 0, 1, and 6-month vaccine clinic visits. The investigator, or his/her designee, will record the pregnancy test result for the 0 month vaccination clinic visit on the Initial Visit Eligibility Screener (ES) form and the pregnancy test result for the 1 and 6-month visits on the Post-Enrollment Eligibility Screener (PES) form. Subjects whose pregnancy test yields a positive result at enrollment cannot receive the study vaccine. The participant will be examined for eligibility for the study once again when she is at least three months postpartum and no longer breastfeeding. If the participant is pregnant at the 1-month or 6-month visit she will have her participation in the study deferred until she is at least three months postpartum and no longer breastfeeding. At that time she will be eligible to continue in the study.

The PES, PO (Amended: 13 Nov 2008), PED (Amended: 29 Mar 2005) and CIF forms contain questions about pregnancies and pregnancy outcomes that have occurred since the last study visit. The principal investigator can also receive information about new pregnancies and pregnancy outcomes through other direct or indirect contacts with participants in the study (e.g., at home reminder visits). All new pregnancy and pregnancy outcome information for study participants, whether or not an SAE is involved, will be transmitted to the Westat Regulatory Associate via a read-only electronic mail report within 5 business days (i.e., no longer than ***40*** business hours) (Amended: 13 Nov 2008, ***Amended: 10 September 2009***) of receipt by the principal investigator or designee. The Westat Regulatory Associate will, in turn, transmit the electronic mail to the NCI Medical Monitor and GSK Global Clinical Safety and Pharmacovigilance within one business day of receipt of the electronic mail from Costa Rica, documenting the receipt date, file name and transmittal date. While pregnancy itself is not considered an AE or SAE, all pregnancies will be followed to term.

A complication of pregnancy or delivery will be recorded as an AE or an SAE, as described in Section 1 and Section 2, and will be followed as described in Section 8. (Amended: 13 Nov 2008) A miscarriage is always considered an SAE and will be reported as outlined in Section 8. (Amended: 13 Nov 2008) Furthermore, SAEs occurring as a result of a post-study pregnancy AND which the investigator considers reasonably related in time to receipt of the investigational product, will be reported to the Westat Regulatory Associate as in Section 8. (Amended: 13 Nov 2008) While the investigator is not obligated to actively seek this information from former study participants, he/she may learn of a pregnancy through spontaneous reporting. Information on pregnancies identified during the screening phase/prior to first vaccine administration does not need to be communicated as a safety issue.

12. Treatment of adverse events

Treatment of adverse events is at the discretion of the investigator and according to current good medical practice. Medications taken by a subject for treatment of an AE that are either immunosuppressive or that the physician deems may help clarify some aspect of the AE, whether or not administered by a study physician, will be recorded in Part C, section II of the AER form.

HPV-009 Vaccine Efficacy Trial in Costa Rica

Figure 3 Expedited Reporting – Personnel Communication Flow Chart (Amended: 13 Nov 2008)

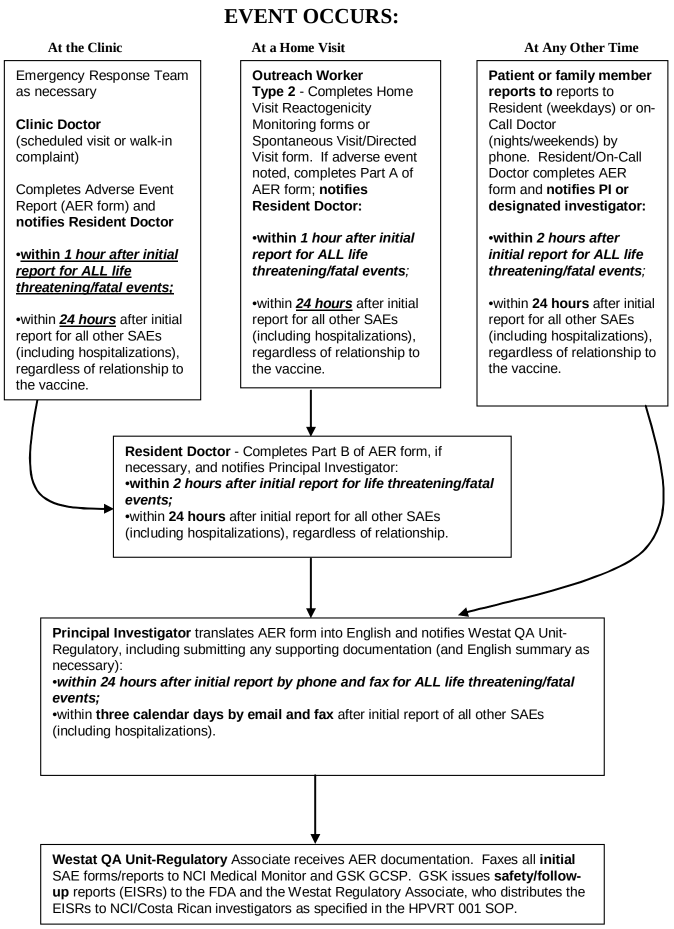

Appendix E HPV16/18 VLP & HAVRIX MSDSs

SDS Number 127745 Approved/Revised 1/6/04 Version 03 04:53:16

MATERIAL SAFETY DATA SHEET

PROPHYLACTIC HUMAN PAPILLOMAVIRUS VACCINE

SECTION 1 IDENTIFICATION OF THE SUBSTANCE/PREPARATION AND OF THE COMPANY/UNDERTAKING

Material PROPHYLACTIC HUMAN PAPILLOMAVIRUS VACCINE

Synonyms HPVpro Vaccine

Company Name GlaxoSmithKline, Corporate Environment, Health & Safety
980 Great West Road
Brentford, Middlesex TW8 9GS UK
UK General Information: +44-20-8047-5000
Transport Emergency +44-1865-407333 (EU)
Medical Emergency +1-612-221-3999, Ext. 221
Information and Advice: US number, available 24 hours
Multi-language response
GlaxoSmithKline, Corporate Environment, Health & Safety
2200 Renaissance Blvd, Suite 105
King of Prussia, PA 19406 US
US General Information: +1-888-825-5249
Transport Emergency +1-703-527-3887 (non EU)
US number, available 24 hours
Multi-language response

SECTION 2 COMPOSITION / INFORMATION ON INGREDIENTS

Ingredients	CAS RN	Percentage
HPV-16 L1 VIRUS-LIKE PARTICLES	Unassigned	<0.01
HPV-18 L1 VIRUS-LIKE PARTICLES	Unassigned	<0.01
NON-HAZARDOUS INGREDIENTS	Unassigned	99.98

SECTION 3 HAZARDS IDENTIFICATION

Fire and Explosion	This product is classified as non-flammable.
Health	Handling this product in its final form presents minimal risk from occupational exposure. Health effects information is based on hazards of components.
Environment	No information is available about the potential of this product to produce adverse environmental effects.

SECTION 4 FIRST-AID MEASURES

Ingestion	Never attempt to induce vomiting. Do not attempt to give any solid or liquid by mouth if the exposed subject is unconscious or semi-conscious. Wash out the mouth with water. If the exposed subject is fully conscious, give plenty of water to drink. Obtain medical attention.
Inhalation	Physical form suggests that risk of inhalation exposure is negligible.
Skin Contact	Using appropriate personal protective equipment, remove contaminated clothing and flush exposed area with large amounts of water. Obtain medical attention if skin reaction occurs, which may be immediate or delayed.
Eye Contact	Wash immediately with clean and gently flowing water. Continue for at least 15 minutes. Obtain medical attention.

NOTES TO HEALTH PROFESSIONALS

Medical Treatment	None.
Medical Conditions Caused or Aggravated by Exposure	The components contained in this vaccine are generally not considered to cause disease in humans.
Antidotes	No specific antidotes are recommended.

SECTION 5 FIRE-FIGHTING MEASURES

Fire and Explosion Hazards	Not expected for the product, although the packaging is combustible.
Extinguishing Media	Water is recommended for fires involving packaging.

Special Firefighting Procedures	For single units (packages): No special requirements needed. For larger amounts (multiple packages/pallets) of product: Since toxic, corrosive or flammable vapours might be evolved from fires involving this product and associated packaging, self contained breathing apparatus and full protective equipment are recommended for firefighters. If possible, contain and collect firefighting water for later disposal.
Hazardous Combustion Products	Toxic, corrosive or flammable thermal decomposition products are expected when the product is exposed to fire.

SECTION 6 ACCIDENTAL RELEASE MEASURES

Personal Precautions	Wear protective clothing and equipment consistent with the degree of hazard.
Environmental Precautions	For large spills, take precautions to prevent entry into waterways, sewers, or surface drainage systems.
Clean-up Methods	Collect and place it in a suitable, properly labelled container for recovery or disposal.
Decontamination Procedures	Water can be used for clean-up and decontamination operations. Sodium hypochlorite (bleach) or other strong oxidizers can be used in clean-up decontamination operations. Contaminated surfaces should be washed with water, then bleach or other oxidizing solution, followed by another water wash.

SECTION 7 HANDLING AND STORAGE

HANDLING

General Requirements	No special control measures required for the normal handling of this product. Normal room ventilation is expected to be adequate for routine handling of this product.
STORAGE	No storage requirements necessary for occupational hazards.

DO NOT FREEZE. Dispose of properly if frozen.

SECTION 8 EXPOSURE CONTROLS/PERSONAL PROTECTION

Other Equipment or Procedures None required for normal handling. Wash hands and arms thoroughly after handling.

SECTION 9 PHYSICAL AND CHEMICAL PROPERTIES

Appearance

Clarity Turbid liquid after shaking.

Colour Colourless supernatant and white deposit after sedimentation.

Physical Form Suspension.

SECTION 10 STABILITY AND REACTIVITY

Stability DO NOT FREEZE - dispose of properly if frozen.

Conditions to Avoid None for normal handling of this product.

SECTION 11 TOXICOLOGICAL INFORMATION

Oral Toxicity Not expected to be toxic following ingestion.

Inhalation Toxicity No studies have been conducted.

Skin Effects Irritation is not expected following direct contact.

Eye Effects Irritation is not expected following direct contact with eyes.

Sensitisation Sensitisation (allergic skin reaction) is not expected.

Genetic Toxicity Not expected to be genotoxic under occupational exposure conditions.

Carcinogenicity Not expected to produce cancer in humans under occupational exposure conditions. No components are listed as carcinogens by GSK, IARC, NTP or US OSHA.

Reproductive Effects Not expected to produce adverse effects on fertility or development under occupational exposure conditions.

SECTION 12 ECOLOGICAL INFORMATION

Summary No information is available about the potential of this product to produce adverse environmental effects. Local regulations and procedures should be consulted prior to environmental release.

SECTION 13 DISPOSAL CONSIDERATIONS

Disposal Recommendations	The disposal method for rejected products/returned goods must ensure that they cannot be re-sold or re-used.
Regulatory Requirements	Observe all local and national regulations when disposing of this product.

SECTION 14 TRANSPORT INFORMATION

The SDS should accompany all shipments for reference in the event of spillage or accidental release. Only authorised persons trained and competent in accordance with appropriate national and international regulatory requirements may prepare dangerous goods for transport.

UN Classification and Labelling

Transport Information	Transportation and shipping of this product is not restricted. It has no known, significant hazards requiring special packaging or labelling for air, maritime, US or European ground transport purposes.

SECTION 15 REGULATORY INFORMATION

The information included below is an overview of the major regulatory requirements. It should not be considered to be an exhaustive summary. Local regulations should be consulted for additional requirements.

EU Classification and Labelling	Exempt from requirements of EU Dangerous Preparations directive - product regulated as a medicinal product, cosmetic product or medical device.

US OSHA Standard (29 CFR Part 1910.1200)

Classification	This product is exempt from the requirements of the OSHA Hazard Communication Standard.

Other US Regulations

TSCA Status	Exempt

SECTION 16 OTHER INFORMATION

References	GSK Hazard Determination

Date Approved/Revised	1/6/04	SDS Version Number 3	04:53:16

SDS Sections Updated

Sections	Subsections
HANDLING AND STORAGE	General Requirements
HANDLING AND STORAGE	Handling
HANDLING AND STORAGE	Ignition Controls
HANDLING AND STORAGE	Protective Systems
HANDLING AND STORAGE	Storage
PHYSICAL AND CHEMICAL PROPERTIES	Autoignition Temperature
PHYSICAL AND CHEMICAL PROPERTIES	Boiling Point
PHYSICAL AND CHEMICAL PROPERTIES	Clarity
PHYSICAL AND CHEMICAL PROPERTIES	Colour
PHYSICAL AND CHEMICAL PROPERTIES	Conductivity
PHYSICAL AND CHEMICAL PROPERTIES	Dissociation Constant
PHYSICAL AND CHEMICAL PROPERTIES	Dust Electrostatic Properties
PHYSICAL AND CHEMICAL PROPERTIES	Dust Explosion
PHYSICAL AND CHEMICAL PROPERTIES	Evaporation Rate
PHYSICAL AND CHEMICAL PROPERTIES	Explosive Properties
PHYSICAL AND CHEMICAL PROPERTIES	Flammability
PHYSICAL AND CHEMICAL PROPERTIES	Flash Point
PHYSICAL AND CHEMICAL PROPERTIES	Freezing Point
PHYSICAL AND CHEMICAL PROPERTIES	Gas Group/T-Rating
PHYSICAL AND CHEMICAL PROPERTIES	General
PHYSICAL AND CHEMICAL PROPERTIES	Lower Explosive Limit
PHYSICAL AND CHEMICAL PROPERTIES	Melting Point
PHYSICAL AND CHEMICAL PROPERTIES	Octanol Water Distribution Coefficient

PHYSICAL AND CHEMICAL PROPERTIES	Odour
PHYSICAL AND CHEMICAL PROPERTIES	Oxidising Properties
PHYSICAL AND CHEMICAL PROPERTIES	Oxygen Balance
PHYSICAL AND CHEMICAL PROPERTIES	Packaging
PHYSICAL AND CHEMICAL PROPERTIES	Particle Size
PHYSICAL AND CHEMICAL PROPERTIES	pH of Aqueous Solutions
PHYSICAL AND CHEMICAL PROPERTIES	Physical Form
PHYSICAL AND CHEMICAL PROPERTIES	Refractive Index
PHYSICAL AND CHEMICAL PROPERTIES	Relative Density
PHYSICAL AND CHEMICAL PROPERTIES	Solubility (Other)
PHYSICAL AND CHEMICAL PROPERTIES	Surface Tension
PHYSICAL AND CHEMICAL PROPERTIES	Train Fire
PHYSICAL AND CHEMICAL PROPERTIES	Upper Explosive Limit
PHYSICAL AND CHEMICAL PROPERTIES	Vapour Density
PHYSICAL AND CHEMICAL PROPERTIES	Vapour Pressure
PHYSICAL AND CHEMICAL PROPERTIES	Viscosity
PHYSICAL AND CHEMICAL PROPERTIES	Volatile Components
PHYSICAL AND CHEMICAL PROPERTIES	Water Solubility
REGULATORY INFORMATION	European Union Classification and Labelling Requirements
REGULATORY INFORMATION	Other Regulations
REGULATORY INFORMATION	Other US Regulations - California Proposition 65
REGULATORY INFORMATION	Other US Regulations – TSCA Status
REGULATORY INFORMATION	State Regulations
REGULATORY INFORMATION	Summary

REGULATORY INFORMATION	US Environmental (EPA) Requirements
REGULATORY INFORMATION	US OSHA Standard (29 CFR Part 1910.1200) - Classification
REGULATORY INFORMATION	US OSHA Standard (29 CFR Part 1910.1200) - Target Organ Stat

The information and recommendations in this safety data sheet are, to the best of our knowledge, accurate as of the date of issue. Nothing herein shall be deemed to create any warranty, express or implied. It is the responsibility of the user to determine the applicability of this information and the suitability of the material or product for any particular purpose.

SDS Number 530 Approved/Revised 1/6/04 Version 12 04:45:31

Material HAVRIX SAFETY DATA SHEET

SECTION 1 IDENTIFICATION OF THE SUBSTANCE/PREPARATION AND OF THE COMPANY/UNDERTAKING

Material HAVRIX

Synonyms HEPATITIS A VACCINE INACTIVATED * HAVRIX 720 *

HAVRIX 360 * HAVRIX 1440 * HAVRIX MONODOSE *

HAVRIX JUNIOR * HAVRIX JUNIOR MONODOSE * NDC

NO. 58160-837-01 * NDC NO. 58160-835-01 * NDC

NO. 58160-835-11 * NDC NO. 58160-835-16 * NDC

NO. 58160-835-32 * NDC NO. 58160-835-35 * NDC

NO. 58160-835-41 * NDC NO. 58160-835-46 * NDC

NO. 58160-835-50 * NDC NO. 58160-837-11 * NDC

NO. 58160-837-26 * NDC NO. 58160-837-28 * NDC

NO. 58160-837-32 * NDC NO. 58160-837-34 * NDC

NO. 58160-837-35 * NDC NO. 58160-837-37 * NDC

NO. 58160-837-41 * NDC NO. 58160-837-42 * NDC

NO. 58160-837-46 * NDC NO. 58160-837-50 * NDC

NO. 58160-837-56 * NDC NO. 58160-837-58

Company Name GlaxoSmithKline, Corporate Environment, Health & Safety
980 Great West Road
Brentford, Middlesex TW8 9GS UK
UK General Information: +44-20-8047-5000
Transport Emergency +44-1865-407333 (EU)
Medical Emergency +1-612-221-3999, Ext. 221
Information and Advice: US number, available 24 hours
Multi-language response

GlaxoSmithKline, Corporate Environment, Health & Safety
2200 Renaissance Blvd, Suite 105
King of Prussia, PA 19406 US
Transport Emergency (non EU) +1-703-527-3887
US number, available 24 hours
Multi-language response

SECTION 2 COMPOSITION / INFORMATION ON INGREDIENTS

Ingredients	CAS RN	Percentage
HAVRIX	Not applicable	
NON-HAZARDOUS INGREDIENTS	Unassigned	>99

SECTION 3 HAZARDS IDENTIFICATION

Fire and Explosion This product is classified as non-flammable.

Health Handling this product in its final form presents minimal risk from occupational exposure. May produce allergic skin reactions. Possible effects of overexposure in the workplace include: symptoms of hypersensitivity (such as skin rash, hives, itching, and difficulty breathing). Health effects information is based on hazards of components.

Environment No information is available about the potential of this product to produce adverse environmental effects.

SECTION 4 FIRST-AID MEASURES

Ingestion Never attempt to induce vomiting. Do not attempt to give any solid or liquid by mouth if the exposed subject is unconscious or semi-conscious. Wash out the mouth with water. If the exposed subject is fully conscious, give plenty of water to drink. Obtain medical attention.

Inhalation Physical form suggests that risk of inhalation exposure is negligible.

Skin Contact Using appropriate personal protective equipment, remove contaminated clothing and flush exposed area with large amounts of water. Obtain medical attention if skin reaction occurs, which may be immediate or delayed.

Eye Contact Wash immediately with clean and gently flowing water. Continue for at least 15 minutes. Obtain medical attention.

NOTES TO HEALTH PROFESSIONALS

Medical Treatment None.

Medical Conditions Caused or Aggravated by Exposure — The components contained in this vaccine are generally not considered to cause disease in humans.

Antidotes — No specific antidotes are recommended.

SECTION 5 FIRE-FIGHTING MEASURES

Fire and Explosion Hazards — Not expected for the product, although the packaging is combustible.

Extinguishing Media — Water is recommended for fires involving packaging.

Special Firefighting Procedures — For single units (packages): No special requirements needed. For larger amounts (multiple packages/pallets) of product: Since toxic, corrosive or flammable vapours might be evolved from fires involving this product and associated packaging, self contained breathing apparatus and full protective equipment are recommended for firefighters. If possible, contain and collect firefighting water for later disposal.

Hazardous Combustion Products — Toxic, corrosive or flammable thermal decomposition products are expected when the product is exposed to fire.

SECTION 6 ACCIDENTAL RELEASE MEASURES

Personal Precautions — Wear protective clothing and equipment consistent with the degree of hazard.

Environmental Precautions — For large spills, take precautions to prevent entry into waterways, sewers, or surface drainage systems.

Clean-up Methods — Collect and place it in a suitable, properly labelled container for recovery or disposal.

Decontamination Procedures — Water can be used for clean-up and decontamination operations. Sodium hypochlorite (bleach) or other strong oxidizers can be used in clean-up decontamination operations. Contaminated surfaces should be washed with water, then bleach or other oxidizing solution, followed by another water wash.

SECTION 7 HANDLING AND STORAGE

HANDLING

General Requirements — No special control measures required for the normal handling of this product. Normal room ventilation is expected to be adequate for routine handling of this product.

STORAGE — No storage requirements necessary for occupational hazards. DO NOT FREEZE. Dispose of properly if frozen.

SECTION 8 EXPOSURE CONTROLS/PERSONAL PROTECTION

Other Equipment or Procedures — None required for normal handling. Wash hands and arms thoroughly after handling.

SECTION 9 PHYSICAL AND CHEMICAL PROPERTIES

Appearance

Clarity — Turbid liquid after shaking.

Colour — Colourless supernatant and white deposit after sedimentation.

Physical Form — Suspension.

SECTION 10 STABILITY AND REACTIVITY

Stability — DO NOT FREEZE - dispose of properly if frozen.

Conditions to Avoid — None for normal handling of this product.

SECTION 11 TOXICOLOGICAL INFORMATION

Oral Toxicity — Not expected to be toxic following ingestion.

Inhalation Toxicity — No studies have been conducted.

Skin Effects — Irritation is not expected following direct contact.

Eye Effects — Irritation is not expected following direct contact with eyes.

Sensitisation — Allergic skin reactions might occur following dermal exposure. Assessment based upon effects of structurally similar substances. Respiratory sensitisation (allergic) reactions might occur following exposure.

Genetic Toxicity — Not expected to be genotoxic under occupational exposure conditions.

Carcinogenicity — Not expected to produce cancer in humans under occupational exposure conditions. No components are listed as carcinogens by GSK, IARC, NTP or US OSHA.

Reproductive Effects — Not expected to produce adverse effects on fertility or development under occupational exposure conditions.

SECTION 12 ECOLOGICAL INFORMATION

Summary — No information is available about the potential of this product to produce adverse environmental effects. Local regulations and procedures should be consulted prior to environmental release.

SECTION 13 DISPOSAL CONSIDERATIONS

Disposal Recommendations — The disposal method for rejected products/returned goods must ensure that they cannot be re-sold or re-used.

Regulatory Requirements — Observe all local and national regulations when disposing of this product.

SECTION 14 TRANSPORT INFORMATION

The SDS should accompany all shipments for reference in the event of spillage or accidental release. Only authorised persons trained and competent in accordance with appropriate national and international regulatory requirements may prepare dangerous goods for transport.

UN Classification and Labelling

Transport Information — Transportation and shipping of this product is not restricted. It has no known, significant hazards requiring special packaging or labelling for air, maritime, US or European ground transport purposes.

SECTION 15 REGULATORY INFORMATION

The information included below is an overview of the major regulatory requirements. It should not be considered to be an exhaustive summary. Local regulations should be consulted for additional requirements.

EU Classification and Labelling

Exempt from requirements of EU Dangerous Preparations directive - product regulated as a medicinal product, cosmetic product or medical device.

US OSHA Standard (29 CFR Part 1910.1200)

Classification — This product is exempt from the requirements of the OSHA Hazard Communication Standard.

Other US Regulations

TSCA Status Exempt

SECTION 16 OTHER INFORMATION

References GSK Hazard Determination

Date Approved/Revised 1/6/04 SDS Version Number 12 04:45:31

SDS Sections Updated

Sections	Subsections
PHYSICAL AND CHEMICAL PROPERTIES	
REGULATORY INFORMATION	European Union Classification and Labelling Requirements

The information and recommendations in this safety data sheet are, to the best of our knowledge, accurate as of the date of issue. Nothing herein shall be deemed to create any warranty, express or implied. It is the responsibility of the user to determine the applicability of this information and the suitability of the material or product for any particular purpose.

Appendix F PROTOCOL AMENDMENTS AND MODIFICATIONS

Amendment 1: 29 March 2005

GlaxoSmithKline Biologicals<br>Clinical Research & Development<br>**Amendment Approval Form**	
**CPMS number**	NCI Protocol: 04-C-N191<br>GSK Biologicals Protocol: 580299/009 (HPV-009)
**Protocol title:**	A double blind, controlled, randomized, phase III study of the efficacy of an HPV16/18 VLP vaccine in the prevention of advanced cervical intraepithelial neoplasia (CIN2, CIN3, adenocarcinoma *in situ* [AIS] and invasive cervical cancer) associated with HPV16 or HPV18 cervical infection in healthy young adult women in Costa Rica
**Amendment no.**	1
**Amendment date:**	1 March 2005
**Coordinating author:**	Diane C Sullivan, RN (Manager, Pediatric Vaccines)

Rationale/background for changes: The primary reasons for this amendment are:

- to clarify the referral of women with evidence of ASC-H and AGC for colposcopic evaluation in Section 3.1,
- to correct the instructions with regard to the reporting of concomitant medications for all AEs, not just those considered as serious in Section 3.9,
- to further clarify the period of observation for the primary analysis of efficacy in Section 4.2,
- to replace all references to the Spontaneous Report (SR) form with the Adverse Event Report (AER) form in Section 3.14 and in multiple sections of Appendix D,
- to further clarify the timing of AE reporting, the SAE contact information and replacement of the Expedited Investigator Safety Report (EISR) with IND Safety Report (INDSR) in Appendix D,
- to expand the pregnancy reporting period in Section 11 of Appendix D, and
- to reformat the protocol to ensure it is compliant with electronic publishing guidelines.

Other minor revisions have been made and are noted throughout the protocol in ***bold, italic*** text.

Details of the revision and identification of the affected protocol sections are provided below. **Amended text has been included in *bold, italic* text and deleted text is shown with ~~strikethrough~~.**

Sponsor Signatory page:
Gary Dubin, MD, Vice President, ~~Worldwide STD Vaccines Clinical Development Programs and US Adult Vaccines Franchise~~ ***Worldwide Clinical Development, HPV Vaccines (Amended: 29 Mar 2005)***

Investigator Agreement

I agree:

- Not to implement any changes to the protocol without agreement from the sponsors ***(NCI and GSK Biologicals) (Amended: 29 Mar 2005)*** and prior review and written approval from the Institutional Review Board (IRB) or Independent Ethics Committee (IEC), …
- That I have been informed that certain regulatory authorities require the sponsor ***(GSK Biologicals) (Amended: 29 Mar 2005)*** to obtain and supply, as necessary, details about the investigator's ownership interest in the sponsor or the investigational product, and more generally about his/her financial ties with the sponsor ***(GSK Biologicals) (Amended: 29 Mar 2005)***. GSK Biologicals will use and disclose the information solely for the purpose of complying with regulatory requirements.

List of Abbreviations

INDSR:	***Investigational New Drug Safety Report (Amended: 29 Mar 2005)***
MSDS:	***Material Safety Data Sheet (Amended: 29 Mar 2005)***
~~SR:~~	***~~Spontaneous Report form~~ (Deleted: 29 Mar 2005)***

SYNOPSIS

**Rationale**	The trial will enroll approximately 15,000 healthy young women… or with 720 enzyme-linked immunoabsorbent assay (ELISA) units of GSK ***Biologicals' (Amended: 29 Mar 2005)*** hepatitis A vaccine, inactivated (Havrix®) prepared as an investigational formulation.
**Study Design (continued)**	• **Duration of the study:**<br>• At least 30 confirmed cases of CIN2+ associated with HPV16 or HPV18 cervical infection are observed and a minimum ***(Amended: 29 Mar 2005)*** follow-up period of two years is achieved, pending review and approval of the final statistical analysis plan by the DSMB.

1.1 Background

Results from a recently completed Phase IIb trial conducted by GSK Biologicals confirm the finding and extend it to HPV18. ~~Unpublished results (manuscript in preparation) from a recently completed phase IIb trial conducted by GSK Biologicals confirm the finding and extend it to HPV18.~~ ***(Amended: 29 Mar 2005)***

3.1 Overview of the Trial

Women with evidence of HSIL, ***ASC-H, AGC (Amended: 29 Mar 2005)*** or persistent ASC-US+/LSIL at any time during the study will be referred for colposcopic evaluation and subsequent excisional treatment, if needed.

3.9 Concomitant Medications/Vaccination

…Also, concomitant medications administered during this study will be collected in the context of assessing ~~S~~AEs. In specific, we will collect information on medications/vaccinations that are taken in the month preceding an ~~S~~AE and those that are taken to treat an ~~S~~AE if it is felt that the medication used to treat the ~~S~~AE can help clarify the diagnosis of the ~~S~~AE or if the medication is an immunosuppressant. ***(Amended: 29 Mar 2005; SAE revised to AE throughout paragraph)***

3.12 Active Follow-up of Participants (Months 12-48)

- An interview to obtain information on ~~potential vaccine~~ ***(Amended: 29 Mar 2005)*** AEs, including hospitalizations, incapacitation lasting more than one day, pregnancies, and other events reported by participants. AEs and SAEs are further defined in APPENDIX IV.

3.14 Adverse Events Monitoring

- **Long-term passive monitoring.** The ~~Spontaneous Report and~~ Adverse Event Report ***(Amended: 29 Mar 2005)*** form will be used to document all AEs reported through this mechanism. ***This*** ~~These~~ form~~s~~ ***is*** ~~are~~ provided separately from this protocol. ***(Amended: 29 Mar 2005)***

3.19.5 HPV DNA Testing by Hybrid Capture® 2 (HCII)

(for baseline/exit assessment and triage of ASC cytology)

An ASC-***US*** cytologic interpretation will lead to "reflex" HCII testing and for the purposes of clinical management subjects who have HCII positive (or HCII-inadequate) ASC-US will be treated the same as an LSIL ~~and subjects who have a HCII positive (or HCII-inadequate) ASC-H will be treated the same as an HSIL~~ ***(Amended: 29 Mar 2005)***.

4.2 Primary Analysis for Efficacy

- At least 30 confirmed cases of CIN2+ associated with HPV16 or HPV18 cervical infection that are eligible for the ATP analysis are observed and ***the last woman recruited into the study has completed*** a minimum ~~median~~ follow-up period of two years ~~is achieved~~, pending review and approval of the final statistical analysis plan by the DSMB. ***(Amended: 29 Mar 2005)***

Assuming 15,000 women enroll, … The requirement of a minimum ~~median~~ ***(Amended: 29 Mar 2005)*** follow-up time of two years was chosen to guard against analyses that are biased by early events only, which in the case of CIN2+ among young women might not be representative of true cancer precursors.

6 Human Subjects Protection

The HPV16/18 VLP Vaccine Trial will be conducted in compliance with regulations for human subject research in effect in Costa Rica and the United States [The Belmont Report & Title 45 Code of Federal Regulations, Part 46, Protection of Human Subjects, and Declaration of Helsinki 1996, ***including its 2000 amendment (Amended: 29 Mar 2005)].***

9 PROJECTED Timetable

Review of Protocol by External Advisory Group	Mar. '03
Submission of Concept Protocol to the FDA	June '03
NCI Human Subjects Annual Review	Jul. '03
Costa Rican Human Subjects Annual Review	Aug. '03
Submission of CMC & Full Study Protocol to FDA	Jan. '04
First Batch of Vaccine Doses for Study Delivered to Costa Rica	Apr. '04
Training	May '04
Initiation of Main Trial ***(Amended: 29 Mar 2005)***	June ***28***, '04
Enrollment of Participants ***(Amended: 29 Mar 2005)***	June '04 – ~~Sep~~ ***Oct.*** '05
Vaccination of Participants	June '04 – Apr. '06
Active Follow-up of Participants ***(Amended: 29 Mar 2005)***	June '04 – ~~Sep~~ ***Oct.*** '09
Study Close-out ***(Amended: 29 Mar 2005)***	~~Oct~~ ***Nov.*** '09 – ~~Jan~~ ***Feb.*** '10

APPENDIX D: CRITERIA FOR DEFINING ADVERSE EVENTS (AEs), SERIOUS AEs (SAEs), AND PROCEDURES FOR AND TIMING OF AE REPORTING

5. Time period, frequency, and method of detecting adverse events and serious adverse events

The standard time period for collecting and recording SAEs will begin ***after receipt of the participant's informed consent signature*** and will end ***at the concluding visit, which will occur at approximately*** 48 months following administration of the first dose of study vaccine for each subject. ***(Amended: 29 Mar 2005)***…

Additionally, in order to fulfill international reporting obligations, SAEs that are related to study participation (e.g., procedures, invasive tests, a change from existing therapy) or are related to a concurrent medication will be collected and recorded from the time of ~~enrolment vaccination (Month 0)~~ ***consent*** until the subject completes ~~48 months of follow-up from the vaccination~~ ***her concluding visit*** or withdraws from the study (e.g., early withdrawal from the study, loss to follow-up). ***(Amended: 29 Mar 2005)***

The investigator will record all AEs during the entire active phase (including the ***approximately*** 48-month follow-up after ~~administration of the first dose of vaccine at Month 0~~ ***consent (Amended: 29 Mar 2005),*** …

The AER and any other relevant forms [e.g., VAR, HV2, ~~SR~~ ***(Amended: 29 Mar 2005)***] will be completed for every report that meets the definition of an adverse event.

…Spontaneous reports of solicited adverse events that are reported by phone to the physician on-call or in-person during a vaccination visit at the clinic will be documented on the ~~SpontaneousReport/Directed Visit , SR~~ ***Adverse Event Report (AER)*** form ***(Amended: 29 Mar 2005)***.

5.1 Solicited adverse events

…With the exception of this sample of 10% of participants who are scheduled to receive a home visit, the ~~SR~~ ***AER (Amended: 29 Mar 2005)*** form will be used to follow up on all other AEs that occur in days 0-6 after vaccination. For example, the ~~SR~~ ***AER (Amended: 29 Mar 2005)*** form will be used to follow up on spontaneously reported AEs as well as AEs that are identified during physician directed home visits.

5.1.1 Solicited local (injection site) AEs

Standardized data will be collected for local injection site AEs on the VAR ***and***, HV2 ~~and SR~~ ***(Amended: 29 Mar 2005)*** for pain, redness, swelling, and temperature at the injection site.

5.1.2.3 Spontaneous reports of solicited AEs

Spontaneous reports of AEs will be captured ***directly*** on the ***AER*** ~~SR~~ form. ~~For each solicited general AE that is reported, the same format that is used on the HV2 form will be used to record AE information on the SR form (i.e., does the woman still have the condition, what was the maximum intensity of the condition, and how long did she have the condition). If a solicited general AE meets the criteria of a SAE, this is indicated on the SR form and an AER form is completed.~~ ***(Amended: 29 Mar 2005)***

5.2 Unsolicited AEs

The VAR~~, SR~~ ***and*** HV2 forms have fields for the collection of information on unsolicited conditions that are observed or reported after vaccination (VAR form) or ~~since the last study visit~~ ***at the 3-6 day home visit following vaccination for 10% of the women*** (HV2, ~~SR~~ forms). When ***completing these forms, if*** any unsolicited AEs are reported, the person completing the form is instructed to complete an AER. ***Also, unsolicited AEs reported outside the context of a clinic visit or a home visit on Days 3-6 after vaccination for 10% of women will be documented directly on the AER form. (Amended: 29 Mar 2005)***

6. Evaluating adverse events and serious adverse events

6.1.2 Other signs and symptoms

The area of local injection site redness/swelling will each be measured in millimeters and recorded on the relevant forms (i.e., the VAR~~, SR~~ ***or*** HV2 form). ***(Amended: 29 Mar 2005)***

6.1.3 Fever

Fever will be recorded on the VAR~~, SR~~ ***or*** HV2 ***(Amended: 29 Mar 2005)*** form, as appropriate, and will be categorized into severity/intensity categories for analysis purposes according to the following oral temperature cut-offs:

8. Prompt reporting of serious adverse events

8.2 Completion and transmission of serious adverse event reports to Westat

**Westat Regulatory Associate for Reporting SAEs**<br>***Mujadala Abdul-Majid, Regulatory Affairs Assistant (Amended: 29 Mar 2005)***<br>***Health Studies Area,*** Westat, Inc.<br>***1441 West Montgomery Ave., Westbrook*** WB 3***35***<br>Rockville, MD 20850<br>Tel: ***301-738-3642*** (direct)<br>Urgent phone: 240-453-5661<br>Fax: 240-314-2547<br>Email: ***mujadala@westat.com***<br><br>~~Zari Anthony, MS, Regulatory Associate~~<br>~~1650 Research Blvd., WB 329~~<br>~~Tel: 240-453-5643~~<br>~~Email: zarianthony@westat.com~~
**GSK Global Clinical Safety and Pharmacovigilance (GCSP) Email and Fax Addresses for Reporting SAEs and Pregnancy Information**<br>**For general safety inquiries:**<br>***Desma Altobelli, PharmD (Amended: 29 Mar 2005)***<br>***Principal*** Clinical Safety Scientist<br>Global Clinical Safety and Pharmacovigilance<br>Tel: 919-483-5***176***<br>Fax: 919-483-5404<br>E-Mail: ***desma.j.altobelli@gsk.com***<br><br>~~Amy W. Mertrud, RPh~~<br>~~Senior Clinical Safety Scientist~~<br>~~Amy.e.mertrud@gsk.com~~<br>~~Tel: 919-483-5417~~

9. Regulatory reporting requirements for serious adverse events

…A given SAE may qualify as an IND Safety Report ***(INDSR)*** if the SAE is both attributable to the investigational product and unexpected. In this case, all investigators conducting studies under the IND (and associated INDs for the same compound) will receive an ***INDSR (previously referred to as*** Expedited Investigator Safety Report ***or*** EISR) ~~identical in content to the IND Safety Report submitted to the FDA.~~ ***(Amended: 29 Mar 2005)***

INDSRs ~~EISR~~ are prepared according to the IND holder's policy and are forwarded by the IND holder to the Westat Regulatory Associate for distribution to NCI and Costa Rica investigators. The purpose of the ***INDSR*** ~~EISR~~ is to fulfil specific regulatory and Good Clinical Practice (GCP) requirements regarding the product under investigation. When the NCI and Costa Rica investigators receive an initial or follow-up ***INDSR*** ~~EISR~~ or

other safety information (e.g., revised Investigator Brochure) from the Westat Regulatory Associate, it will be their responsibility to notify the DSMB and the local and NCI IRBs. ***(Amended: 29 Mar 2005)***

11. Pregnancy

The PES, ***PED,*** ~~SR~~ and CIF forms contain questions about pregnancies and pregnancy outcomes that have occurred since the last study visit. ***(Amended: 29 Mar 2005)***…
All new pregnancy and pregnancy outcome information for study participants, whether or not an SAE is involved, will be transmitted to the Westat Regulatory Associate via a read-only electronic mail report within ~~72 hours~~ ***3-5 days (i.e., no longer than 120 hours) (Amended: 29 Mar 2005)*** of receipt by the principal investigator or designee.

Amendment 2: 31 October 2005

GlaxoSmithKline Biologicals<br>Clinical Research & Development<br>**Amendment Approval Form**	
**CPMS number**	NCI Protocol: 04-C-N191<br>GSK Biologicals Protocol: 580299/009 (HPV-009)
**Protocol title:**	A double blind, controlled, randomized, phase III study of the efficacy of an HPV16/18 VLP vaccine in the prevention of advanced cervical intraepithelial neoplasia (CIN2, CIN3, adenocarcinoma *in situ* [AIS] and invasive cervical cancer) associated with HPV16 or HPV18 cervical infection in healthy young adult women in Costa Rica
**Amendment no.**	2
**Amendment date:**	31 October 2005
**Coordinating author:**	Diane C Sullivan, RN (Manager, Pediatric Vaccines)

Rationale/background for changes: The primary reasons for this amendment are:

- Increasing the post-vaccination observation period from 30 to 60 minutes at the request of the local IRB. Many of the study centers are located in remote areas of Costa Rica where there is limited access to medical care, so to assure that there is no immediate severe reaction to vaccination, the observation period has been increased.
- Expanded explanation for the deferment of enrollment or vaccination visit for menstrual delay or pregnancy.
- A change to the “Back-up Westat Regulatory Associate for Reporting SAEs”.
- Expanded period of recruitment (16 to 18 months).

Other minor revisions have been made and are noted throughout the protocol in ***bold, italic*** text.

Details of the revision and identification of the affected protocol sections are provided below. **Amended text has been included in *bold, italic* text and deleted text is shown with ~~strikethrough~~.**

**Study Design**	**Enrollment:** Population-based, ages 18-25, N= ~15,000 (of ~20,000 who will be invited, based on 2000 census data). The expected enrollment period is ~~16~~ ***18 (Amended: 31 Oct 2005)*** months.<br><br>**Safety assessments:**<br>In all subjects during the ~~30~~ ***60***-minute ***(Amended: 31 Oct 2005)*** post-vaccination period, solicited local AEs (pain, redness, swelling and temperature at the injection site) and solicited general AEs with new onset or aggravation since vaccination (fever, fatigue, myalgia, arthralgia, gastrointestinal symptoms, headache, rash [other than urticaria] and urticaria) will be assessed.
**Endpoints**	**Secondary**<br>1. Safety<br>• Occurrence and intensity of solicited local AEs and occurrence (either onset or aggravation) of solicited general AEs including urticaria within ~~30~~ ***60 (Amended: 31 Oct 2005)*** minutes after vaccination.

3. Methods

3.1 Overview of the Trial

…The initial vaccination will be performed at entry, and additional vaccine doses will be given at one and six months following the initial vaccination. Women will be monitored at the clinic for a minimum of ~~30~~ ***60 (Amended: 31 Oct 2005)*** minutes following each vaccination, during which study personnel will collect information on reactogenicity of the vaccine. …

3.3.3 Deferral Criteria

Women will have their enrollment into the trial ***and/or vaccination visits (Amended: 31 Oct 2005)*** deferred if they:

- Received immunoglobulins within 90 days preceding enrollment***/vaccination visit (Amended: 31 Oct 2005).***
- Received a registered vaccine within 30 days of enrollment***/vaccination visit.*** Administration of routine Meningococcal, Hepatitis B, Influenza, and Diphtheria/Tetanus vaccine***s*** up to 8 days before ***any*** ~~the first~~ dose of study vaccine is allowed. ***(Amended: 31 Oct 2005)***
- Are suffering from an acute disease or have an oral temperature ≥37.5°C at enrollment***/vaccination visit (Amended: 31 Oct 2005).*** Acute disease is defined as the presence of a moderate or severe illness with or without fever. Vaccines can be administered to women with a minor illness such as diarrhea and mild upper respiratory infection.
- Are pregnant, less than three months post-partum, or breastfeeding at the time of enrollment***/vaccination visit.*** If the participant reports a ***menstrual delay and her pregnancy test is negative,*** ~~missed period~~ she will be deferred ***for a minimum of seven days*** ~~independently of the results of her pregnancy test.~~ ***Women with a second negative pregnancy test after the minimal deferral period would be eligible for enrollment/vaccination. A menstrual delay is defined as a delay of more than five***

days for women with regular menses and more than 35 days for women with irregular menses. (Amended: 31 Oct 2005)

- Are sexually experienced and using an effective method of birth control (including abstinence) for less than 30 days at the time of enrollment***/vaccination visit (Amended: 31 Oct 2005).***

3.7 Selection of an Investigational Formulation of *Havrix* as the Control Vaccine

Second, *Havrix* 720 EL.U./0.25 mg Alum in 0.5 mL (pediatric formulation) is currently licensed in the US for use in children aged ***12 months (Amended: 31 Oct 2005)*** ~~2~~ to 18 years on a 0, 6-12 month schedule. The 720 EL.U. dose has been demonstrated to be safe for use in children in numerous clinical trials and through post-marketing surveillance. This suggests that the formulation can be safely administered to adults.

3.11. Clinic Visits During Vaccination Phase (0, 1, 6, & 7 months)

Three clinic visits are planned for vaccination at 0, 1, and 6 months. At the first clinic visit, we will collect information for determining eligibility, ensure that written informed consent has been obtained prior to any study procedure, perform urine pregnancy testing, assign treatment arm by randomization, give the initial vaccination, and monitor women for at least ~~30~~ ***60 (Amended: 31 Oct 2005)*** minutes following vaccination with appropriate medical treatment readily available in case of a rare anaphylactic reaction. At the two subsequent visits at 1 and 6 months, information on AEs will be collected, subsequent vaccinations will be administered, and women will be monitored for a minimum of ~~30~~ ***60 (Amended: 31 Oct 2005)*** minutes following vaccination with appropriate medical treatment readily available in case of a rare anaphylactic reaction. After each vaccination visit, women will be provided with two 500 mg acetaminophen pills with an indication to take the medication in case of a mild fever or pain. The use of acetaminophen during the trial will be followed in the 10% home visit subset. A summary of the procedures performed at each of these visits is presented in Table 2. Intervals between study visits during the vaccination phase of the trial are provided in Table 4.

Table 4 Intervals Between Study Visits

A. Yearly Visit Schedule

Interval	Length of interval (days)
1 (Month 0→Month 1)	21 to 62
2 (Month 0→Month 6)	161 to 216

B. Six-Monthly Visit Schedule

Interval***	Length of interval (days)
1 (Month 0→Month 1)	21 to 62
2 (Month 0→Month 6)	161 to 216

* This interval applies only to the first 600 women enrolled, i.e., the women in the immunogenicity subset.
**Date of the Month 6 visit serves as the reference date.
***This schedule is to be followed by women detected as having evidence of LSIL (regardless of HPV testing results) or ASC-US concomitant with detection of an oncogenic HPV type.
N.B. Except for Month 7 where the date of the Month 6 visit serves as the reference date, the date of the first vaccination serves as the reference date for intervals between study visits. *Also, for Intervals 1 and 2, the length of the interval listed represents the desired interval. Broader, allowable intervals are defined in Table 2. (Amended: 31 Oct 2005)*

3.13.2. Secondary Endpoints

In addition to histopathologically confirmed CIN2+ and HPV16 and HPV18 infection, which are described above, other endpoints required to achieve the six secondary objectives of our trial are discussed below.

To evaluate the safety of our vaccine (Secondary Objective #1), we plan to examine:

1. Occurrence and intensity of solicited local adverse events and occurrence (either onset or aggravation) of solicited general adverse events including urticaria within ~~30~~ ***60 (Amended: 31 Oct 2005)*** minutes after each vaccination and over all vaccinations combined.

3.14. Adverse Events Monitoring

- **AEs occurring in the week following vaccination. …**The information collected during this visit is summarized on the Home Visit Reactogenicity Monitoring and A***ER*** ~~dverse Event Report~~ ***(Amended: 31 Oct 2005)*** forms that are provided separately from this protocol.
- **AEs occurring immediately following vaccination.** We will observe all vaccinees for at least ~~30~~ ***60 (Amended: 31 Oct 2005)*** minutes at the clinic following each vaccination with appropriate medical treatment readily available in case of a rare anaphylactic reaction. This will be performed under the supervision of trained medical staff. During this ~~30~~ ***60 (Amended: 31 Oct 2005)*** minute observation period, we will collect information on vaccine reactogenicity, including both solicited and unsolicited events, as summarized on the Vaccine Administration and Reactogenicity Monitoring (VAR) and ~~Adverse Event Report (~~***AER***~~)~~ ***(Amended: 31 Oct 2005)*** forms that are provided separately from this protocol.

9. PROJECTED Timetable

Review of Protocol by External Advisory Group	Mar '03
Submission of Concept Protocol to the FDA	June '03
NCI Human Subjects Annual Review	Jul '03
Costa Rican Human Subjects Annual Review	Aug '03
Submission of CMC & Full Study Protocol to FDA	Jan '04
First Batch of Vaccine Doses for Study Delivered to Costa Rica	Apr '04
Training	May '04
Initiation of Main Trial *(Amended: 29 Mar 2005)*	June *28*, '04
Enrollment of Participants *(Amended: 29 Mar 2005; **31 Oct 2005**)*	June '04 – ~~Oct~~ ***Dec***'05
Vaccination of Participants ***(Amended: 31 Oct 2005)***	June '04 – ~~Apr~~ ***June*** '06
Active Follow-up of Participants *(Amended: 29 Mar 2005; **31 Oct 2005**)*	June '04 – ~~Oct~~ ***Dec*** '09
Study Close-out *(Amended: 29 Mar 2005; **31 Oct 2005**)*	*Nov* '09 – ~~Feb~~ ***Apr*** '10

Appendix D CRITERIA FOR DEFINING ADVERSE EVENTS (AEs), SERIOUS AEs (SAEs), AND PROCEDURES FOR AND TIMING OF AE REPORTING

5. Time period, frequency, and method of detecting adverse events and serious adverse events

The AER and any other relevant forms (e.g., VAR, HV2) *(Amended: 29 Mar 2005)* will be completed for every report that meets the definition of an adverse event. The investigator will attempt detection of AEs and SAEs in the following ways: For all subjects the investigator will monitor and record the occurrence of reactions in the first ~~30~~ ***60 (Amended: 31 Oct 2005)*** minutes following administration of the vaccine at every vaccination visit (baseline [0 month], 1 month and 6 months) using the Vaccine Administration and Reactogenicity Monitoring (VAR) form. …

When an AE/SAE occurs, it is the responsibility of the investigator to review all documentation (e.g., hospital progress notes, laboratory, and diagnostics reports) relative to the event. The investigator will then record all relevant information regarding an AE/SAE on the AER form. There may be instances when the NCI Medical Monitor will request additional abstraction of information from the participant's medical records. GlaxoSmithKline (GSK) Biologicals, hereafter referred to as the IND holder, may also request additional information, but will do so via the ***NCI Medical Monitor or NCI Project Officer (Amended: 31 Oct 2005)*** ~~Westat Regulatory Associate~~. In these instances, all subject identifiers will be removed by the investigator prior to document transmission. …

5.1 Solicited adverse events

For all subjects the investigator will monitor and record the occurrence of reactions in the first ~~30~~ ***60 (Amended: 31 Oct 2005)*** minutes following administration of the vaccine at every vaccination visit (baseline [0 month], 1 month and 6 months) using the VAR form.

5.1.2.1 Thirty Sixty-minute (Amended: 31 Oct 2005) post-vaccination monitoring

Pre-vaccination oral temperature and ~~30~~ ***60***-minute ***(Amended: 31 Oct 2005)*** post-vaccination oral temperature are recorded on the VAR form. …

7. Follow-up of adverse events and serious adverse events and assessment of outcome

Clinically significant laboratory abnormalities will be followed up until they have returned to baseline, or a satisfactory explanation has been provided. Reports relative to the subsequent course of an SAE noted for any subject must be submitted to the Westat Regulatory Associate.

The NCI Medical Monitor or IND holder, via the ***NCI Medical Monitor or NCI Project Officer (Amended: 31 Oct 2005)*** ~~Westat Regulatory Associate~~, may request that the investigator obtain additional information from the participant's clinical records to elucidate as fully as possible the nature and/or causality of the AE or SAE. The investigator is obliged to assist. If a subject dies during participation in the study or during a recognized follow-up period, the investigator will obtain copies of any available post-mortem findings, including histopathology, and will forward them to Westat's Regulatory Associate for appropriate distribution.

Updated information will be recorded on new AER forms linked to the original form with an AE number provided by the study data management system. The updated SAE report form should be resent to the Westat Regulatory Associate within 24 hours of receipt of the follow-up information as outlined in Section 8.

Back-up Westat Regulatory Associate for Reporting SAEs
Meredith Melick (Amended: 31 Oct 2005) Leslie Dondey-Nouvel, MD, Safety Manager<br>Westat, Inc.<br>1650 Research Blvd., WB 352<br>Rockville, MD 20850<br>Tel: 240-453-2688 (Amended: 31 Oct 2005) 301-294-4445 (direct)<br>Urgent Phone: 240-453-5661<br>Fax: 240-314-2547<br>Email: meredithmelick (Amended: 31 Oct 2005) lesliedondey-nouvel@westat.com

Amendment 3: 19 May 2006

GlaxoSmithKline Biologicals<br>Clinical Research & Development<br>**Amendment Approval Form**	
**CPMS number**	NCI Protocol: 04-C-N191<br>GSK Biologicals Protocol: 580299/009 (HPV-009)
**Protocol title:**	A double blind, controlled, randomized, phase III study of the efficacy of an HPV16/18 VLP vaccine in the prevention of advanced cervical intraepithelial neoplasia (CIN2, CIN3, adenocarcinoma *in situ* [AIS] and invasive cervical cancer) associated with HPV16 or HPV18 cervical infection in healthy young adult women in Costa Rica
**Amendment no.**	3
**Amendment date:**	19 May 2006
**Coordinating author:**	Diane C Sullivan, RN (Manager, Pediatric Vaccines)
**Rationale/background for changes:** The primary reasons for this amendment is:<br>• To clarify the subset of 600 study subjects in this trial who will be included in the immunogenicity subcohort.<br>Details of the revision and identification of the affected protocol sections are provided below. **Amended text has been included in *bold, italic* text and deleted text is shown with ~~strikethrough~~.**	

Synopsis

**Objectives (continued)**	6. To evaluate the vaccine immunogenicity (by ELISA and V5/J4 monoclonal antibody inhibition enzyme immunoassay [EIA]) in ~~the first~~ ***a subset of (Amended: 19 May 2006)*** 600 subjects enrolled.
**Study Design (continued)**	**Immunogenicity subcohort:** ~~The first~~ ***A subcohort of (Amended: 19 May 2006)*** 600 subjects enrolled, regardless of the study site at which they are enrolled, will have blood samples taken at Months 0, 6, 7, 12, 24, 36 and 48 for immunogenicity assessments. The immunogenicity subcohort will be comprised of these 600 subjects.<br>Note: All ***other (Amended: 19 May 2006)*** subjects enrolled ~~after the first 600 subjects~~ will have blood drawn at Months 0, 6, 12, 24, 36, and 48 (and at Months 18, 30, and 42 if seen at those time intervals) to allow exploratory analyses and ancillary studies by NCI.
**Endpoints**	HPV16 and HPV18 ELISA and V5/J4 monoclonal antibody inhibition EIA titers in the ~~first~~ 600 subjects enrolled ***into the immunogenicity subcohort. (Amended: 19 May 2006)***

2.2 Secondary Objectives

6. Evaluate the vaccine immunogenicity (by ELISA and V5/J4 monoclonal antibody inhibition EIA) in ~~the first~~ ***a subset of (Amended: 19 May 2006)*** 600 subjects enrolled (immunogenicity subcohort).

Table 7 Timeline of Costa Rican HPV16/18 VLP Vaccine Trial Activities *(Amended: 19 May 2006)*

	Invitation	Vaccination and AE monitoring			Follow-up							
Timing of Home Visit/Office Visit/Contact	-1 month	0 month	1 month	6 months	7 months*	12 months	18 months**	24 months	30 months**	36 months	42 months**	48 months
**For cryopreservation (40 mL)**		●***		●***	●***	●***	●***	●***	●***	●***	●***	●***
Monitoring for solicited & unsolicited symptoms 60 min post-vaccination		●	●	●								

*7 month visit applies only to the ~~first~~ 600 women enrolled *in the immunogenicity subcohort. (Amended: 19 May 2006)*
**Visit is only required if indicated by the case management algorithm. The protocol-specified clinical management algorithm is summarized in Appendix A.
Fup = follow-up; Pre = pre-vaccination; PV = post-vaccination. ● is used to indicate a study procedure that requires documentation in the individual CRF.
Note: The double-line borders following Months 0, 1 and 6 indicate that a home visit by a specially-trained outreach worker to monitor AEs will occur during the week following vaccination (any day from Day 3 to Day 6) in a random subset of 10% of the women.
***This blood draw will be performed on a subset of women, and at selected study visits, as follows: A) A random sample of women at 0, 12, and 36 months; B) All women referred to accelerated screening visits per management algorithm, at yearly intervals; C) All women referred for colposcopic evaluation who require a biopsy or LEEP, at the time of the colposcopy visit; *D) women enrolled in the immunogenicity subcohort after March 1, 2006. (Amended: 19 May 2006)*
†Procedure will not be performed on virginal women.
††Pelvic examination will be performed if indicated by the case management algorithm. The protocol-specified clinical management algorithm is summarized in Appendix A.
¶Reflex HCII HPV testing will be performed automatically for all subjects with results of ASC. HCII HPV testing will also be performed on all women who have a pelvic examination at the 0 month visit.
§HCII testing for *Chlamydia trachomatis* and *Neisseria gonorrhoeae* will be performed on samples obtained at the 0 month visit (or the first visit with a pelvic examination for women who are virginal at entry) only.
^ HPV testing by HCII will be performed on all specimens obtained at the 48 month visit.

3.11. Clinic Visits During Vaccination Phase (0, 1, 6, & 7 months)

To permit characterization of maximal antibody titers achieved after all three doses of vaccine are administered, a blood draw is also planned a minimum of 30 days after the third vaccination (7 month visit) on a subset of ~~the initial~~ ***(Amended: 19 May 2006)*** 600 women randomly assigned to one of the two arms of our trial.

Table 4 Intervals Between Study Visits

C. Yearly Visit Schedule

Interval	Length of interval (days)
1 (Month 0→Month 1)	21 to 62
2 (Month 0→Month 6)	161 to 216
3 (Month 6→Month 7)*	30 to 60**
4 (Month 0→Month 12)	301 to 660
5 (Month 0→Month 24)	661 to 1020
6 (Month 0→Month 36)	1021 to 1380
7 (Month 0→Month 48)	1381 to 1530

D. Six-Monthly Visit Schedule

Interval***	Length of interval (days)
1 (Month 0→Month 1)	21 to 62
2 (Month 0→Month 6)	161 to 216
3 (Month 6→Month 7)*	30 to 60**
4 (Month 0→Month 12)	301 to 480
5 (Month 0→Month 18)	481 to 660
6 (Month 0→Month 24)	661 to 840
7 (Month 0→Month 30)	841 to 1020
8 (Month 0→Month 36)	1021 to 1200
9 (Month 0→Month 42)	1201 to 1380
10 (Month 0→Month 48)	1381 to 1530

* This interval applies only to the 600 women enrolled *in the immunogenicity subcohort (Amended: 19 May 2006)*, i.e., the women in the immunogenicity subset.

3.12. Active Follow-up of Participants (Months 12-48)

- A blood specimen will be collected for HPV antibody and other testing. On a***n approximately (Amended: 19 May 2006)*** 10% subset of women at enrollment and at the ***7 (Amended: 19 May 2006)***, 12 and 36 month visits, additional blood for ancillary studies will be collected to permit cryopreservation of lymphocytes for immunological assessment.

3.1.3.2 Secondary Endpoints

Finally, to confirm that immunogenicity in our population is comparable to that seen in previous Phase I/II trials and to define long-term immunogenicity of the candidate vaccine (Secondary Objective #6), we plan to measure HPV16 and HPV18 antibody levels by ELISA and V5/J4 monoclonal antibody inhibition EIA on blood specimens

collected from the 600 women recruited into ***the immunogenicity subcohort of (Amended: 19 May 2006)*** our trial. Details of the serology testing plan are provided in Section 3.19 (Laboratory Tests)

3.15.1 Overview

All participants are expected to have at least seven visits over the course of our trial (see also Table 2). This will include:

- A clinic visit will be conducted at seven months on a subset of ~~the initial~~ ***(Amended: 19 May 2006)*** 600 women enrolled to allow for immunogenicity assessment a minimum of 30 days following the last vaccination.

3.17 Blood Collection

Three types of blood draws are planned… This third blood draw will be collected for ancillary research purposes on a subset of participants, including a random sample of 10% of participants at enrollment and at their 12 and 36 month visits (to document the distribution of the immune responses in our population before and after vaccination), women referred to accelerated screening visits at six-month intervals (this group would have the additional blood sample collected annually), women who are referred for colposcopic evaluation and require a biopsy or LEEP, ***and women enrolled in the immunogenicity subcohort of our trial after March 1, 2006. (Amended: 19 May 2006)*** While the primary focus of this larger blood draw will be on the cryopreservation of PBMCs for immunological monitoring, residual plasma will also be saved for use in possible future assays.

3.19.7 Serology Testing by ELISA

Serological assays for HPV16/18 by ELISA will be performed at GSK Biologicals laboratories, Rixensart, Belgium or in a laboratory sponsored by the NCI in Costa Rica, pending validation. Baseline ELISA anti-HPV16 and anti-HPV18 testing will be performed on all women enrolled. In addition, anti-HPV16 and anti-HPV18 ELISA will be performed on all blood samples (Month 0 through Month 48) collected from the 600 women enrolled into the ***immunogenicity subcohort within the (Amended: 19 May 2006)*** trial. Women in this immunogenicity subcohort will have an extra clinic visit performed a minimum of 30 days following the last vaccination (Month 7 visit) to permit immunological assessment after all three vaccine doses are administered.

Amendment 4: 13 November 2008

GlaxoSmithKline Biologicals<br>Clinical Research & Development<br>**Amendment Approval Form**	
**CPMS number**	NCI Protocol: 04-C-N191<br>GSK Biologicals Protocol: 580299/009 (HPV-009)
**Protocol title:**	A double blind, controlled, randomized, phase III study of the efficacy of an HPV16/18 VLP vaccine in the prevention of advanced cervical intraepithelial neoplasia (CIN2, CIN3, adenocarcinoma *in situ* [AIS] and invasive cervical cancer) associated with HPV16 or HPV18 cervical infection in healthy young adult women in Costa Rica
**Amendment no.**	4
**Amendment date:**	13 November 2008
**Coordinating author:**	Shamita Gupta

Rationale/background for changes: The primary reasons for this amendment are:

To update the Protocol to reflect the actual enrollment figure of 7,466 subjects as well as revision of statistical assumptions.

To clarify procedures at the final visit (Month 48 visit). Given the rapid changes occurring in clinical management of cervical abnormalities, we stated in the original protocol for our trial that the colposcopy referral algorithm used for the final screening visit under this protocol (Month 48) would be reviewed and updated, as needed, prior to implementation of the 48-month visit. We have updated the protocol to reflect that the same criteria used to define women who require colposcopy evaluation used throughout the trial will be applied at the Month 48 visit. Any additional care deemed necessary by the local clinicians will be conducted after participant close-out under a separate protocol.

To include the collection of extra-cervical specimens for HPV testing (oral, anal, vulvar) at the Month 48 visit, and to include its evaluation as a tertiary objective.

To revise the cytology QC process at the Month 48 visit. We propose to add slides interpreted as negative in Costa Rica with evidence of reactive changes and that are concurrently HPV positive by HCII to the set of slides selected for 100% review in the United States. This increase in the proportion selected for review is being proposed because in an evaluation of the enrollment (pre-vaccination) cytology QC effort we observed that up to 3% (3 out of 114) of slides with reactive changes that are HPV positive might have histologically evident CIN2+. Conversely, we propose to continue to review at the previous 10% level (i.e., not to increase to 100% review) those slides that are classified in Costa Rica as either negative or negative with evidence of reactive changes without concurrent evidence of HPV positivity by HCII because our

evaluation of the enrollment (pre-vaccination) cytology QC effort revealed that 0% (0 of 397) of slides in these two categories were found to have histologically evident CIN2+.

To modify the date range for the Month 48 visit window. The Month 48 window has been modified to be consistent with the length of the other routine visit windows, 360 days, from day 1381-1740. Since the 48-month visit is the last routine study visit, allowances are also made to allow women who cannot otherwise be seen within the pre-defined window.

To clarify the procedures for reporting adverse events in offspring of participants (Section 3.14- Adverse Event Monitoring).

To implement administrative changes including (1) update of sponsor signatory and laboratory name (2) clarification stating that background information in the protocol is reflective of knowledge at study start (3) removing mention of specific names of NCI pathologists responsible for histology review (4) updates to Appendix D including correction of typographical errors in the pregnancy and adverse event reporting sections.

Other minor revisions and corrections have been made and are noted throughout the protocol in ***bold, italic*** text.

Details of the revision and identification of the affected protocol sections are provided below. **Amended text has been included in *bold, italic* text and deleted text is shown with ~~strikethrough~~.**

GSK Sponsor Signatory: (Amended: 13 Nov 2008)
Dominique Descamps
Director, Clinical Development
HPV vaccines, GlaxoSmithKline Biologicals

~~Gary Dubin, MD, Vice President, Worldwide Clinical Development, HPV Vaccines (Amended: 29 Mar 2005)~~
~~2301 Renaissance Blvd~~
~~King of Prussia, PA 19406, USA~~
~~Tel: 610-787-3104~~
~~Fax: 610-787-7057~~
~~Email: Gary.O.Dubin@gsk.com~~

Synopsis

Study Design

Enrollment: ***Randomization of 7,500 women will provide a sufficient study size to evaluate the main trial objectives.*** **(Amended: 13 Nov 2008)**

Management of women with abnormal cytology: Women detected as having evidence of a low-grade squamous intraepithelial lesion (LSIL) (regardless of HPV testing results) or atypical squamous cells of undetermined significance (ASC-US) concomitant with detection of an oncogenic HPV type by Hybrid

Capture II (HCII) will have cytology performed at 6 monthly intervals (rather than yearly intervals). Those whose ASC-US/oncogenic HPV positive lesion or LSIL persists for 2 evaluations (whether consecutive or intermittent) will be referred for colposcopy for evaluation and excisional treatment, if warranted. Those with 3 consecutive normal cytology evaluations will return to an annual cytology evaluation schedule. For purposes of this management strategy, women with ASC-US whose concomitant specimen tests negative for HPV will be considered normal and followed at annual intervals.

Women detected as having evidence of a high-grade squamous intraepithelial lesion (HSIL), atypical glandular cells (endocervical, endometrial, or glandular; favor neoplastic or not otherwise specified) (AGC), atypical squamous cells, cannot exclude HSIL (ASC-H), or cancer will be immediately referred for colposcopic evaluation and excisional treatment, if warranted.

At the Month 48 visit we will apply the same criteria applied throughout the trial to define women who require colposcopic evaluation. Specifically, all women with evidence of cancer, HSIL, glandular lesions, ASC-H, or persistent ASC-US+/LSIL will be referred to colposcopy. Close-out of individual study participants will only occur after colposcopy referral, for women who require such referral.

It should be noted, that after participant close-out, participants will be offered additional clinical care if needed. Case management after study close-out will be defined under a separate protocol. **(Amended: 13 Nov 2008)**

~~In addition to the management described above, exit colposcopy will be performed following the Month 48 visit in all women who, at that time are found to have atypical squamous cells (includes ASC-H and ASC-US) (ASC), LSIL, or more severe disease; women with normal cytology at the Month 48 visit who have cytologically evident abnormalities (ASC or LSIL) detected in the 12 months preceding the Month 48 visit; and a random subset of 400 women selected from among the remaining women with normal cytology at Month 48.~~

~~Note: Given that clinical management options are rapidly evolving, this preliminary exit strategy will be reviewed with the Data and Safety Monitoring Board (DSMB) prior to its implementation. Should the final exit strategy differ from that described here, a protocol amendment will be implemented detailing the exit visit strategy and its rationale.~~

Duration of the study*: Total enrollment was 7,466, which is lower than initial estimates. The final accrual of 7,466 provides 91% power to detect a vaccine efficacy of 80% for the outcome of CIN2+.* (Amended: 13 Nov 2008)

~~Assuming 15,000 women enroll, it is expected that a total of approximately 50 HPV16/18+ CIN2+ events will occur across both trial arms over the course of the trial under the null hypothesis and that 30 events will occur across both trial arms over the course of the trial in the event that the vaccine is 80% effective.~~

1.1 Background

~~This experience is summarized in the GSK Investigator Brochure.~~ ***The information in this section reflects knowledge that was current at the time of study start. Please refer to the most recent edition of the Investigator Brochure for a review of clinical and pre-clinical studies*** **(Amended: 13 Nov 2008).**

2.3 Tertiary Objectives

5. *An evaluation of vaccine efficacy of the candidate vaccine against HPV infections that occur at extra-cervical sites.* (Amended: 13 Nov 2008)

3.1. Overview of the Trial

Approximately 20,000 women will be invited to participate in a double blinded, randomized clinical trial to evaluate whether vaccination with the bivalent HPV16/18 VLP-based vaccine manufactured by GSK Biologicals protects against the development

of histopathologically confirmed, incident CIN2+. Women between 18-25 years of age at entry who are residents of Guanacaste Province or surrounding areas will be eligible. Participants will be required to not be pregnant at the time of vaccination, to agree to use an effective birth control method 30 days before vaccination until 60 days after the last vaccination (approximately 9 months total), and to be in good health as determined by an overall physical examination administered by a study physician at entry into the trial. Women will be identified through a census of the region performed in February/July 2000, and will be invited to participate in the trial by visiting one of the study clinics set up specifically for this trial. ***While randomization (into HPV or HAV vaccination) of 15,000 women was initially anticipated, randomization of 7,500 women will provide a sufficient study size to evaluate the main trial objectives based on revised statistical assumptions, see Section 4.7.*** **(Amended: 13 Nov 2008)** ~~12,000-15,000 women are expected to be eligible and agree to be randomly assigned to one of two study arms: VLP vaccination or vaccination with a control vaccine (HAV).~~

Table 1 Expected Trial Sample Size (Amended: 13 Nov 2008)

Number of women (from census) invited to participate	19,000 – 21,000
Number of eligible women who agree to be randomly assigned to one of two study arms	***7,500***<br>~~12,000 – 15,000~~
Number of women who will be randomly assigned to one of two study arms, will receive 3 doses of vaccine and will be evaluable in ATP analysis	***5,300***<br>~~10,400 – 13,000~~
Estimated ~~N~~number of women who complete four years of follow-up	***6,375***<br>~~9,400 – 11,800~~

Table 2 Timeline of Costa Rican HPV16/18 VLP Vaccine Trial Activities (Amended: 13 Nov 2008)

	Invitation	Vaccination and AE monitoring			Follow-up							
Timing of Home Visit/Office Visit/Contact	-1 month	0 month	1 month	6 months	7 months*	12 months	18 months**	24 months	30 months**	36 months	42 months**	48 months
Desired & [Maximum Allowable] Range in Days relative to Month 0 Visit	-30 to –3	0	21 to 62 [21 to 120]	161 to 216 [121 to 300]	30 to 60 after 6 month visit	301 to 480	481 to 660	661 to 840	841 to 1020	1021 to 1200	1201 to 1380	1381to ~~1530~~ 1740#
Type of Visit/Contact	Invitation	Enrolment & Vaccine #1	One Month & Vaccine #2	Six Month & Vaccine #3	Immuno-genicity Subset	1st Fup	Interim Fup	2nd Fup	Interim Fup	3rd Fup	Interim Fup	4th Fup
Sampling timepoint		Pre	PV I	PV II	PV III	PV III	PV III	PV III	PV III	PV III	PV III	PV III
Contact potential subjects by mail	•											
Visit potential subjects at their homes and provide them with a copy of the informed consent form	•											
Informed consent		•										
Check eligibility/inclusion criteria		•										
Check exclusion criteria		•										
Check vaccination deferral criteria		•	•	•								
Check elimination criteria		•	•	•	•	•	•	•	•	•	•	•
Check contraindications		•	•	•								
Interview (Questionnaire)		•		•		•	•	•	•	•	•	•
Complete medical history		•										
Interim medical history			•	•	•	•	•	•	•	•	•	•
Physical examination		•										
Pre-vaccination body temperature		•	•	•								
Urine sampling for urine pregnancy test (50 mL)		•	•	•								
Randomization		•										

	Invitation	Vaccination and AE monitoring			Follow-up							
Timing of Home Visit/Office Visit/Contact	-1 month	0 month	1 month	6 months	7 months*	12 months	18 months**	24 months	30 months**	36 months	42 months**	48 months
Desired & [Maximum Allowable] Range in Days relative to Month 0 Visit	-30 to –3	0	21 to 62 [21 to 120]	161 to 216 [121 to 300]	30 to 60 after 6 month visit	301 to 480	481 to 660	661 to 840	841 to 1020	1021 to 1200	1201 to 1380	1381to ~~1530~~ 1740#
Type of Visit/Contact	Invitation	Enrolment & Vaccine #1	One Month & Vaccine #2	Six Month & Vaccine #3	Immuno-genicity Subset	1st Fup	Interim Fup	2nd Fup	Interim Fup	3rd Fup	Interim Fup	4th Fup
Sampling timepoint		Pre	PV I	PV II	PV III	PV III	PV III	PV III	PV III	PV III	PV III	PV III
Blood sampling												
For antibody test/research (10 mL)		●	●	●	●	●	●	●	●	●	●	●
For hematocrit (3 mL)		●										
For research (10 mL)		●										●
For cryopreservation (40 mL)		●***		●***	●***	●***	●***	●***	●***	●***	●***	●***
Pelvic examination†		●		●††		●	●	●	●	●	●	●
Collection of cervical secretions for research†		●		●††		●	●	●	●	●	●	●
Collection of cervical sample for:<br>-cervical cytology,<br>-HPV PCR testing, and<br>-HCII testing†¶		●§		●††		●	●††	●	●††	●	●††	●^
Collection of subject-obtained cervical sample for HPV PCR testing†				●								
Collection of cervical cells for research†		●		●††		●	●	●	●	●	●	●
Vaccination		●	●	●								
Monitoring for solicited & unsolicited symptoms 60 min post-vaccination		●	●	●								

	Invitation	Vaccination and AE monitoring			Follow-up							
Timing of Home Visit/Office Visit/Contact	-1 month	0 month	1 month	6 months	7 months*	12 months	18 months**	24 months	30 months**	36 months	42 months**	48 months
Desired & [Maximum Allowable] Range in Days relative to Month 0 Visit	-30 to –3	0	21 to 62 [21 to 120]	161 to 216 [121 to 300]	30 to 60 after 6 month visit	301 to 480	481 to 660	661 to 840	841 to 1020	1021 to 1200	1201 to 1380	1381to ~~1530~~ 1740#
Type of Visit/Contact	Invitation	Enrolment & Vaccine #1	One Month & Vaccine #2	Six Month & Vaccine #3	Immuno-genicity Subset	1st Fup	Interim Fup	2nd Fup	Interim Fup	3rd Fup	Interim Fup	4th Fup
Sampling timepoint		Pre	PV I	PV II	PV III	PV III	PV III	PV III	PV III	PV III	PV III	PV III
On one day from Day 3-6 after vaccination, a specially-trained outreach worker will interview a random subset of 10% of the 15,000 subjects for follow-up regarding post-vaccination solicited & unsolicited symptoms		•	•	•								
Recording of AEs (nonserious and serious) occurring during the period since the last visit			•	•	•	•	•	•	•	•	•	•
Recording of any concomitant medication/vaccination taken in the 30 days prior to SAE onset that could have contributed to the SAE		•	•	•	•	•	•	•	•	•	•	•
Recording of any concomitant medication/vaccination used to treat an SAE, that clarifies the diagnosis of the SAE or is an immunosuppressant		•	•	•	•	•	•	•	•	•	•	•
Reporting of all pregnancies and all pregnancy outcome information		•	•	•	•	•	•	•	•	•	•	•
Study Conclusion												•
Collection of Oral HPV Samples												•
Collection of Anal HPV Samples												•
Collection of Vulvar HPV Samples												•

*7 month visit applies only to the 600 women enrolled *in the immunogenicity subcohort. (Amended: 19 May 2006)*
**Visit is only required if indicated by the case management algorithm. The protocol-specified clinical management algorithm is summarized in Appendix A.
Fup = follow-up; Pre = pre-vaccination; PV = post-vaccination. ● is used to indicate a study procedure that requires documentation in the individual CRF.
Note: The double-line borders following Months 0, 1 and 6 indicate that a home visit by a specially-trained outreach worker to monitor AEs will occur during the week following vaccination (any day from Day 3 to Day 6) in a random subset of 10% of the women.
***This blood draw will be performed on a subset of women, and at selected study visits, as follows: A) A random sample of women at 0, 12, and 36 months; B) All women referred to accelerated screening visits per management algorithm, at yearly intervals; C) All women referred for colposcopic evaluation who require a biopsy or LEEP, at the time of the colposcopy visit; *D) women enrolled in the immunogenicity subcohort after March 1, 2006. (Amended: 19 May 2006).*
†Procedure will not be performed on virginal women.
††Pelvic examination will be performed if indicated by the case management algorithm. The protocol-specified clinical management algorithm is summarized in Appendix A.
¶Reflex HCII HPV testing will be performed automatically for all subjects with results of ASC. HCII HPV testing will also be performed on all women who have a pelvic examination at the 0 month visit.
§HCII testing for *Chlamydia trachomatis* and *Neisseria gonorrhoeae* will be performed on samples obtained at the 0 month visit (or the first visit with a pelvic examination for women who are virginal at entry) only.
^ HPV testing by HCII will be performed on all specimens obtained at the 48 month visit.
#*Every effort will be made to schedule study participants within the preferred Month 48 study visit window (1381 to 1740 days post Month 0). However, the window for the last visit might be extended until October 3, 2010, which is the scheduled date of last subject last visit (i.e, 1740 days after the 4 year anniversary date for the last subject first visit).*

3.6 Study Vaccines

~~To date~~ ***At the time of study start***, five Phase I-IIb clinical trials ~~have been~~ ***were*** conducted ***that*** ~~and have~~ evaluated vaccine safety. (**Amended: 13 Nov 2008**)

Please refer to the Investigator Brochure (IB) ~~(3rd edition, March 2004)~~ for a review of the HPV16/18 VLP vaccine pre-clinical and clinical studies. (**Amended: 13 Nov 2008**)

3.11. Clinic Visits During Vaccination Phase (0, 1, 6, & 7 months)

Table 4 Intervals Between Study Visits (Amended: 13 Nov 2008)

A. Yearly Visit Schedule

Interval	Length of interval (days)
1 (Month 0→Month 1)	21 to 62
2 (Month 0→Month 6)	161 to 216
3 (Month 6→Month 7)*	30 to 60**
4 (Month 0→Month 12)	301 to 660
5 (Month 0→Month 24)	661 to 1020
6 (Month 0→Month 36)	1021 to 1380
7 (Month 0→Month 48)	1381 to ~~1530~~ *1740#*

B. Six-Monthly Visit Schedule

Interval***	Length of interval (days)
1 (Month 0→Month 1)	21 to 62
2 (Month 0→Month 6)	161 to 216
3 (Month 6→Month 7)*	30 to 60**
4 (Month 0→Month 12)	301 to 480
5 (Month 0→Month 18)	481 to 660
6 (Month 0→Month 24)	661 to 840
7 (Month 0→Month 30)	841 to 1020
8 (Month 0→Month 36)	1021 to 1200
9 (Month 0→Month 42)	1201 to 1380
10 (Month 0→Month 48)	1381 to ~~1530~~ *1740#*

* This interval applies only to the 600 women enrolled in the immunogenicity subcohort (Amended: 19 May 2006), i.e., the women in the immunogenicity subset.
**Date of the Month 6 visit serves as the reference date.
***This schedule is to be followed by women detected as having evidence of LSIL (regardless of HPV testing results) or ASC-US concomitant with detection of an oncogenic HPV type.
N.B. Except for Month 7 where the date of the Month 6 visit serves as the reference date, the date of the first vaccination serves as the reference date for intervals between study visits. Also, for Intervals 1 and 2, the length of interval listed represents the desired interval. Broader, allowable intervals are defined in Table 2. (Amended: 31 Oct 2005)
# Every effort will be made to schedule study participants within the preferred Month 48 study visit window (1381 to 1740 days post Month 0). However, the window for the last visit might be extended until October 3, 2010, which is the scheduled date of last subject last visit (i.e, 1740 days after the 4 year anniversary date for the last subject first visit).

3.12. Active Follow-up of Participants (Months 12-48)

Women who at any of the yearly screening visits are found to have evidence of ASC-US+/LSIL will be seen at six-month intervals (rather than yearly intervals), and will continue to be seen every six months until there is evidence of disease

persistence/progression (in which case they will be referred for colposcopic evaluation) or three consecutive normal cytologies are observed (in which case they will return to a yearly screening interval). Women who at any of the clinic visits are found to have evidence or suspicion of ***AGC/ASC-H***/HSIL/cancer will be immediately referred for colposcopic evaluation and excisional treatment, if needed. **(Amended: 13 Nov 2008)**

3.13.3. Tertiary and *Exploratory* Endpoints (Amended: 13 Nov 2008)

3.14 Adverse Events Monitoring

One of the major objectives of the present trial is to obtain additional information on the AEs associated with HPV16/18 VLP vaccination. While safety and immunogenicity trials in the United States and elsewhere have suggested the overall safety of the HPV16/18 VLP-based vaccine, rare AEs cannot be ruled out in these early phase studies given the relatively limited number of volunteers involved (approximately 690 individuals vaccinated ~~to date~~ ***at the time of study start*** with the GSK Biologicals candidate HPV16/18 VLP-based vaccine and approximately 2,200 individuals ~~have been~~ vaccinated ~~to date~~ ***at the time of study start*** with other VLP-based vaccines). **(Amended: 13 Nov 2008)**

- ***Monitoring specific AEs of interest in offspring:***

At the request of the Costa Rican IRB specific adverse events occurring in subject's offspring who were conceived within one month prior to first vaccination to one year after last vaccination will be reported at the final visit (Month 48). This includes the following medical event categories in the offspring:

- ***Endocrinologic and metabolic conditions***
- ***Autoimmune diseases***
- Sensory impairment
- Intellectual impairment
- ***Death***

The occurrence of these specific events in offspring will be included in the clinical database. A child case will be created and linked to the mother case. Medical judgment will be exercised in deciding whether any disorders/diseases reported in offspring are included in these specific categories and should be reported as appropriate. Of note congenital malformations in offspring, as per original protocol, are to be documented and reported as SAEs and included in the pregnancy outcome analysis. **(Amended: 13 Nov 2008)**

All AEs reported on the AER form (Amended: 31 Oct 2005) will be followed through resolution by a staff physician. ***Non-serious AEs will be reviewed by a staff clinician before participant close-out; participants with ongoing conditions will be referred to the appropriate referral center if needed***. **(Amended: 13 Nov 2008)**

3.16.3 Exiting Women from the Trial (Amended 13 Nov 2008)

At the Month 48 visit we will apply the same criteria applied throughout the trial to define women who require colposcopic evaluation. Specifically, all women with

evidence of cancer, HSIL, glandular lesions, AGC, ASC-H, or persistent ASC-US+/LSIL will be referred to colposcopy. Close-out of individual study participants will only occur after colposcopy referral, for women who require such referral. See Appendix A for more details.

It should be noted, that after participant close-out, participants will be offered additional clinical care if needed. Case management after study close-out will be defined under a separate protocol.

~~Before the trial is terminated, it is important that we assure all women in the trial that they are free of disease. Therefore, at the last routine screening visit proposed as part of our trial (at 48 months), the criteria used to determine whether a woman should be referred for colposcopic evaluation will differ from that implemented during the initial years of the trial.~~

~~More specifically, in addition to referring all women with evidence of cancer, HSIL, glandular lesions, ASC-H, or persistent ASC-US+/LSIL to colposcopy, we propose to refer to colposcopy women with evidence of newly diagnosed ASC/LSIL (irrespective of HPV status) and women with normal cytology at the Month 48 visit who have cytologically evident abnormalities (ASC or LSIL) detected in the 12 months preceding the Month 48 visit. A random subset of 400 women will also be referred for colposcopic evaluation to confirm the sensitivity of our clinical management algorithm for the detection of CIN2+. Given that clinical management options are rapidly changing, we further propose that this preliminary exit strategy be reviewed and discussed by the IRBs and DSMB prior to its implementation. The revised strategy will be submitted for review before the first woman recruited reaches her 48 month visit. Should the final exit strategy differ from that summarized here, an amendment will also be filed with the US FDA detailing the exit visit strategy and its rationale. This will assure that state-of-the-art knowledge is incorporated into our exit visit strategy.~~

3.17.1 Overview

- ***Oral, anal and vulvar specimen collection at the 48-month visit is being added to allow for the evaluation of the effect of vaccination on oral, anal, and vulvar HPV infection rates.*** **(Amended: 13 Nov 2008)**.

3.17.4 Extra-Cervical Collection

Oral, anal and vulvar specimen collection at the 48-month visit is being added to allow for the evaluation of the effect of vaccination on oral,anal, and vulvar HPV infection rates. Prior to the pelvic exam, an oral specimen will be collected. During the pelvic examination, a vulvar and an anal specimen will be collected prior to cervical sampling. Each specimen will be collected according to a standard procedure and will be stored in separate tubes. **(Amended: 13 Nov 2008*)***

3.18. Biospecimen Processing, Storage, & Shipment

The individual types of specimens we propose to collect and the expected number of aliquots resultant from this collection and requiring storage/shipment are summarized in

Table 5 to allow NCI to plan for adequate repository space at its main repository in Maryland, USA. In brief, we propose to collect cervical cells for liquid-based cytology, additional cervical cells for HPV DNA, RNA and other testing, cervical secretions for immunological assessment, cytological material (slides) for evaluation, histopathological material (blocks and slides) for review and testing, blood for various immunological and other research-based tests, ***and oral, anal and vulvar samples*** **(Amended: 13 Nov 2008*)***. We have had experience collecting all of these biological specimens in Costa Rica on several thousand women over more than seven years as part of our natural history study.

Several biological specimens we propose to collect require little or no processing and can be stored at air-conditioned room temperature until shipment to the USA. These include the cells for liquid-based cytology and cytopathological materials. Other specimens require little or no processing but need to be frozen on the day of collection. These include the cervical cells for HPV DNA and RNA testing, cervical secretions, ***and the oral, anal and vulvar samples (*****Amended: 13 Nov 2008*****)*** .

Table 5 Types of Biological Specimens Proposed and Expected Storage Requirements (Amended: 13 Nov 2008*)*

Specimen Type	# per Woman	Size	Total #*
Cervical Cells (Liquid Cytology/HPV)	6	25 mL vial	~~90,000~~ *45,000*
Cervical Cells (Research)	6	10 mL vial	~~90,000~~ *45,000*
Cervical Secretions	5	10 mL vial	~~75,000~~ *37,500*
Blood	70	1-2 mL vials	~~1,050,000~~ *525,000*
Tissue Slides (Cytology/Pathology)	6.25	Slides	~~93,750~~ *46,875*
Tissue Blocks	.25	Blocks	~~3,750~~ *1,875*
Oral Samples	*1-3*	*20 mL vial*	*7,500*
Anal Samples	*1*	*1 mL vial*	*7,500*
Vulvar Samples	*1*	*1 mL vial*	*7,500*

* Assuming ~~15,000~~ *7,500* participants (repository estimation purposes; does not take drop-outs into account)

3.19.1 Cytology

The clinical management of the study participants will rely on the Costa Rican cytopathology interpretation, with the exception of HSIL noted below. As part of NCI's ongoing quality control efforts (led by cytotechnologist Claire Eklund and pathologist Martha Hutchinson who are affiliated with the Women's and Infants' Hospital in Providence, RI), all slides that read as abnormal in Costa Rica and a 10% sample of the slides read as negative in Costa Rica will be re-screened and re-interpreted in the United States. ~~In addition, all slides from the study exit visit (Month 48) will be re-screened and re-interpreted in the United States~~. ***At the study exit (Month 48), in addition to 100% of slides read as abnormal in Costa Rica and 10% of the slides read as negative in Costa Rica mentioned above, 100% of slides that are read in Costa Rica as negative with evidence of reactive changes and that are concurrently HPV positive by the HCII will be re-interpreted in the United States (*****Amended: 13 Nov 2008*****).*** The 10% set of negatives selected for evaluation in the United States will be randomly selected, without replacement. This scheme will result in approximately 50% of subjects with a negative cytology having an expert review over the course of the 4-year study follow-up period. The purpose of this review is to maximize the accuracy of the Costa Rican interpretations

and the comparability of the Costa Rican readings to a US standard, realizing nonetheless that cytopathology is inherently and unavoidably subjective to some extent and that there is no absolute reference standard of cytopathology, particularly for equivocal interpretations such as ASC-US.

3.19.2. Histology

In addition to the Costa Rican diagnosis, **an** NCI pathologist ~~(Mark Sherman and/or Diane Solomon)~~ **(Amended: 13 Nov 2008)** with expertise in cervical pathology will periodically diagnose all histological specimens, blinded to the initial Costa Rican diagnosis. If the diagnoses are concordant (concordance will be required at the level of negative or atypical metaplasia/CIN1/CIN2/CIN3 or *in situ*/cancer), that will establish the NCI provisional study diagnosis. Discrepant findings between the Costa Rican and NCI pathologist will lead to review by the second NCI pathologist. Agreement between two of the three pathologists at the <CIN2/CIN2+ level will establish the NCI study diagnosis. These diagnoses will not be available within a timeframe that would allow their use for clinical management purposes. Therefore, NCI pathology review will be performed to define histological diagnoses for analysis purposes. This review process and its results will be performed and documented in the US. A copy of the results of the US pathology review will be sent to the clinical staff in Costa Rica. Periodic review of proficiency slide sets will be performed throughout the trial to assure comparability of the Costa Rican/NCI panel consensus readings against other expert pathologists involved in HPV vaccine studies.

3.19.3. PCR typing of HPV DNA from *PreservCyt* specimens

(to define trial endpoints)

Two 0.5 mL aliquots of *PreservCyt* specimens will be withdrawn *prior* to *ThinPrep* preparation to avoid possible specimen-to-specimen contamination. The removal of the two small aliquots is not expected to impact on the cellular adequacy of the *PreservCyt* specimen, which typically contains many more cells than required for a *ThinPrep* and subsequent Hybrid Capture® 2 (a signal amplification assay; manufactured by Digene Corporation, Gaithersburg, MD, USA) (HCII HPV DNA test) testing.

The PCR testing used to establish the presence of HPV16 and/or HPV18 for the efficacy endpoints will be performed by GSK Biologicals or ~~Delft~~ ***DDL*** **(Amended: 13 Nov 2008)** Diagnostic Laboratories on one *PreservCyt* aliquot, as follows (see also Appendix C).

To test for HPV DNA positivity, GSK Biologicals or ~~Delft~~ ***DDL*** **(Amended: 13 Nov 2008)** Diagnostic Laboratories will use SPF_{10} primers that amplify a 65 nucleotide region of the HPV L1 gene for most of the known HPV isolates.

3.20. Efforts to Maximize Participation/Retention

- We will invite 20,000 women to participate, but our power estimates (see Table 6 Section 4.7) indicate the need for ***7,500*** ~~12,000 – 15,000~~ women (**Amended: 13 Nov**

2008). Only women who, in principle, agree to comply with the full protocol will be enrolled.

4.7 Power to Determine Vaccine Efficacy

Total accrual into the Costa Rica Trial (HPV-009) was lower than initial estimates. For the outcome of histological CIN2+ (expected # events = 24), the final accrual of 7,466 provides 91% power to detect a vaccine efficacy of 80% under the following assumptions:
1) cumulative incidence of histopathologically confirmed CIN2+ associated with HPV-16 or HPV-18 of 0.79% (Woodman***, 2001)***
2) percent of non-virginal women with histologically confirmed CIN2+ at enrolment of 1.5%
3) proportion of women who are sexually inexperienced at entry of 23%
4) Proportion of virginal women who initiate sexual activity during follow-up of 43% (constant rate of initiation assumed during the 4-year follow-up period)
5) proportion of sexually experienced women who are both HPV-16 and HPV-18 DNA positive at entry of 0.6%
6) proportion of sexually experienced women who acquire HPV-16/18 during the vaccination phase of 1.5% (includes women who develop incident CIN2+ associated with HPV-16/18 infection during this period)
7) proportion of women who receive 1+ vaccine doses outside the prescribed protocol window of 20%
8) proportion of women who drop out during the study period of 10% (constant drop-out rate assumed during the 4-year follow-up period)
9) a two-sided alpha of 0.05. **(Amended: 13 Nov 2008)**

Appendix A

- Added Colposcopy Referral Algorithm - Month 48 Visit

Amended: 13 Nov 2008

Colposcopy Referral Algorithm- Month 48 Visit

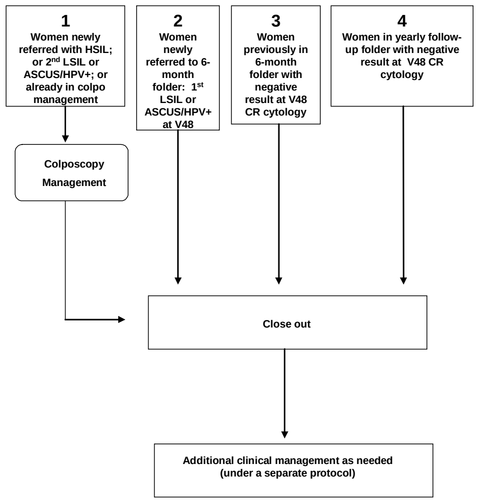

Appendix D

7. Follow-up of adverse events and serious adverse events and assessment of outcome

The updated SAE report form should be resent to the Westat Regulatory Associate within 24 hours of receipt of the follow-up information as outlined in Section ~~0~~*8*. **(Amended: 13 Nov 2008)**

8.2 Completion and transmission of serious adverse event reports to Westat

Mujadala Abdul-Majid, Regulatory Affairs Assistant (Amended: 13 Nov 2008)
Health Studies Area, Westat, Inc.
1441 West Montgomery Ave., Westbrook, WB 335
Rockville, MD 20850
Tel: 301-738-3642 (direct)
Urgent phone: 240-453-5661
Fax: 240-314-2547
Email: mujadala@westat.com

10. Post study adverse events and serious adverse events

A post-study AE/SAE is defined as any event that occurs outside of the AE/SAE detection period defined in Section 5. Investigators are not obligated to actively seek AEs or SAEs in former study participants. If the investigator learns of any SAE, including a

death, at any time after a subject has been discharged from the study, and considers the event reasonably related to the investigational product, the investigator will ***notify the sponsor.*** ~~promptly notify Westat's Regulatory Associate~~. ***(*Amended: 13 Nov 2008*)***

11. Pregnancy

The PES, ***PO*** (**Amended: 13 Nov 2008*)***, PED (Amended: 29 Mar 2005) and CIF forms contain questions about pregnancies and pregnancy outcomes that have occurred since the last study visit. The principal investigator can also receive information about new pregnancies and pregnancy outcomes through other direct or indirect contacts with participants in the study (e.g., at home reminder visits). All new pregnancy and pregnancy outcome information for study participants, whether or not an SAE is involved, will be transmitted to the Westat Regulatory Associate via a read-only electronic mail report within ~~3~~ 5 ***business*** days (i.e., no longer than 120 ***business*** hours) **(Amended: 13 Nov 2008)** of receipt by the principal investigator or designee. The Westat Regulatory Associate will, in turn, transmit the electronic mail to the NCI Medical Monitor and GSK Global Clinical Safety and Pharmacovigilance within one business day of receipt of the electronic mail from Costa Rica, documenting the receipt date, file name and transmittal date. While pregnancy itself is not considered an AE or SAE, all pregnancies will be followed to term.

A complication of pregnancy or delivery will be recorded as an AE or an SAE, as described in Section 1 and Section 2, and will be followed as described in Section ~~0~~ ***8***. **(Amended: 13 Nov 2008)** A miscarriage is always considered an SAE and will be reported as outlined in Section ~~0~~ ***8***. **(Amended: 13 Nov 2008)** Furthermore, SAEs occurring as a result of a post-study pregnancy AND which the investigator considers reasonably related in time to receipt of the investigational product, will be reported to the Westat Regulatory Associate as in Section ~~0~~ ***8.*** **(Amended: 13 Nov 2008)** While the investigator is not obligated to actively seek this information from former study participants, he/she may learn of a pregnancy through spontaneous reporting. Information on pregnancies identified during the screening phase/prior to first vaccine administration does not need to be communicated as a safety issue.

Figure 3 Expedited Reporting – Personnel Communication Flow Chart (Amended: 13 Nov 2008)

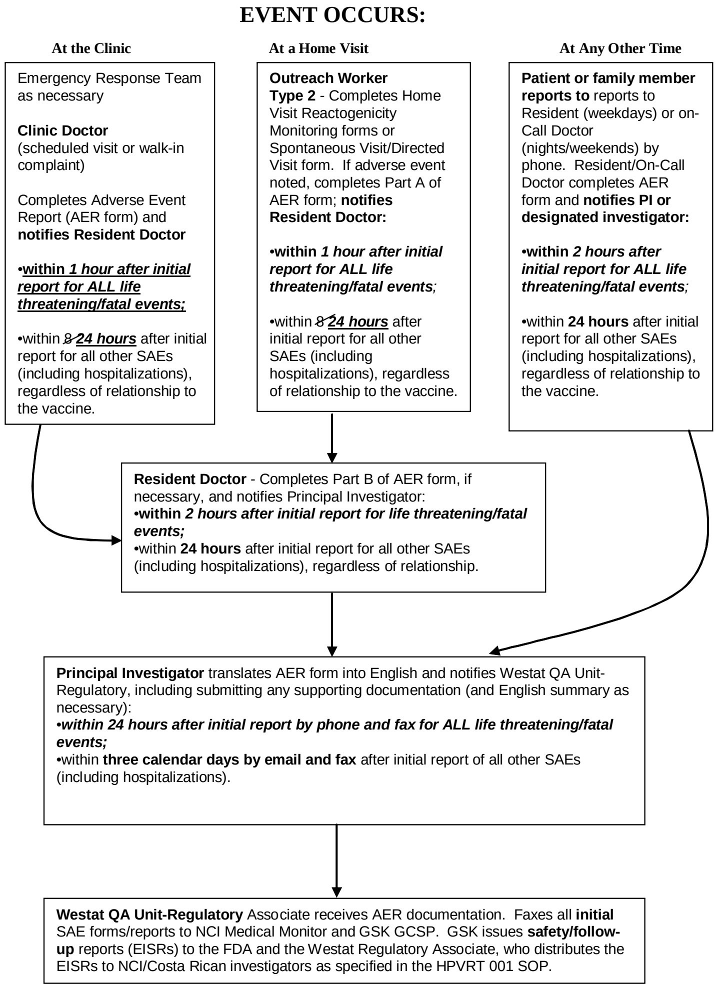

Amendment 5: 10 September 2009

GlaxoSmithKline Biologicals<br>Clinical Research & Development<br>**Amendment Approval Form**	
**CPMS number**	NCI Protocol: 04-C-N191<br>GSK Biologicals Protocol: 580299/009 (HPV-009)
**Protocol title:**	A double blind, controlled, randomized, phase III study of the efficacy of an HPV16/18 VLP vaccine in the prevention of advanced cervical intraepithelial neoplasia (CIN2, CIN3, adenocarcinoma *in situ* [AIS] and invasive cervical cancer) associated with HPV16 or HPV18 cervical infection in healthy young adult women in Costa Rica
**Amendment no.**	5
**Amendment date:**	10 September 2009
**Coordinating author:**	Ruchita Nadkarni

Rationale/background for changes: The primary reasons for this amendment are:

- To update the criteria for collection of heparinized blood (green cap tubes) from women who are being followed every six month due to a low-grade cytological finding.

  Starting on March 31, 2009, we have stopped the collection of additional herparinized blood from women who are being followed at 6-month intervals. Given that more than 3000 green cap samples from the second group of women listed above (annually for women in the six month folder) has been collected, sufficient numbers from which we can sample for nested studies has been reached. Additionally, the processing of these samples is very expensive, and with the study exit visit, the colopscopy effort has intensified. We will continue collecting green caps on the following groups of women:
    - PID 5 during V36
    - Immunogenicity sub-cohort during V36
    - Tissue collection at colposcopy (one collection per colposcopy cycle, if possible at the first tissue collection)

- To clarify the methods for using self-collected samples. To clarify that women who test positive for the corresponding HPV type at the 6-month visit by either the clinician or self-administered collection methods used will be excluded from the ATP analysis.
- To define the alpha-spending plan for the 5th secondary objective to account for the interim analysis proposed in the Statistical Analysis Plan (SAP) for Initial Analyses that was previously submitted to the FDA.

A SAP for initial analyses that focuses on virological endpoints has been developed and submitted to the FDA. To account for this SAP and for the fact that one of its objectives overlaps with the 5th secondary objective listed in the protocol, an alpha spending plan has been incorporated into the protocol.

- To include mention that pathologists responsible for histology review are NCI-***designates***, and not exclusively NCI pathologists in section 3.19.2.

  With the replacement of Dr. Diane Solomon with Dr. Mary Sidawy in protocol amendment #4, our pathology team is composed of both NCI pathologists and NCI designated pathologists. We have added wording to clarify this point in the protocol.

- To include the use of Twinrix® as a crossover vaccination vaccine.

  In order to reduce clinic visits, women who are eligible to recieve both the hepatitis A vaccine and hepatitis B vaccine during the crossover vaccination will be offered vaccination with Twinrix® [Hepatitis A Inactivated & Hepatitis B (Recombinant) Vaccine].

- To clarify close-out activities at study completion.

  To avoid unnecessary delays in offering crossover vaccination to women who have completed their 4 years of participation in the study, we will conduct data clean-up and data freeze after completion of study activities in a staggered/batched fashion to enable a more timely unblinding for crossover.

- Other minor revisions have been made and are noted throughout the protocol in ***bold, italic*** text.

Details of the revision and identification of the affected protocol sections are provided below. **Amended text has been included in *bold, italic* text and deleted text is shown with ~~strikethrough~~.**

Footnote for Table 2 Timeline of Costa Rican HPV16/18 VLP Vaccine Trial Activities

This blood draw will be performed on a subset of women, and at selected study visits, as follows: A) A random sample of women at 0, 12, and 36 months; B) All women referred to accelerated screening visits per management algorithm, at yearly intervals ***before March 31, 2009 (Amended: 10 September 2009); C) All women referred for colposcopic evaluation who require a biopsy or LEEP, at the time of the colposcopy visit; D) women enrolled in the immunogenicity subcohort after March 1, 2006.

3.3.4. Elimination criteria from the ATP analyses during the study

For the primary according to protocol (ATP) analysis planned for our study, the following elimination criteria may apply. These are also discussed in the statistical analysis section of this protocol (Section 4.2).

- Women who do not receive all three doses of the vaccine within the protocol-specified intervals (see Table 4).
- Women who are found to be HPV16 and HPV18 DNA positive ***(for the corresponding type considered in the analysis)*** during the vaccination phase***, by clinician and/or self-administered collection*** (months 0 or 6) ***(Amended: 10 September 2009)***.

3.5 Trial Arms and Randomization

Since knowledge of vaccine arm assignment would not affect treatment of participants who have an SAE, no provision is made to allow for unblinding on site in Costa Rica. Unblinding of individual participants, at the request of the DSMB, the IRBs, the GSK Global Clinical Safety Department or the NCI Medical Monitor as part of reporting requirements to the FDA (e.g., for rapid reporting of an unexpected SAE associated with vaccination) will be allowed. This individual unblinding will be performed in a manner that assures that the overall study blinding is maintained, that staff involved in the conduct, analysis, or reporting of the study remain blinded except where absolutely impractical, and that any unblinding be appropriately documented ***(see also Section 3.16.3, Exiting Women from the Trial) (Amended: 10 September 2009)***. Treatment codes will be kept at NCI and GSK under controlled/secured access.

3.6 Study Vaccines

It should be noted that after completion of this trial and under a separate protocol, participants will be offered cross-over vaccination. This will include offering the licensed formulation of *Havrix* (1440 ELISA units/0.5 mL) hepatitis A vaccine to women who received the HPV16/18 VLP vaccine and the ***licensed*** HPV16/18 VLP vaccine to women who received the investigational hepatitis A vaccine ~~(if the HPV16/18 VLP vaccine is found to be effective and the DSMB and Costa Rica IRB approve)~~ ***(Amended: 10 September 2009)***. In addition, all women will be offered vaccination with Engerix-B® (Hepatitis B Vaccine [Recombinant]; Manufactured by GlaxoSmithKline Biologicals, Rixensart, Belgium). ***In order to reduce clinic visits, women who are eligible to recieve both the hepatitis A vaccine and hepatitis B vaccine will be offered vaccination with Twinrix® [Hepatitis A Inactivated & Hepatitis B (Recombinant) Vaccine] (Amended: 10 September 2009).***

3.16.3. Exiting Women from the Trial (Amended: 13 Nov 2008)

Activities at study completion (Amended: 10 September 2009)

The Data and Safety Monitoring Board (DSMB) for our trial has recommended cross-over immunization of both treatment and control recipients. The participants who received the HPV vaccine will be offered vaccination against hepatitis A and hepatitis B and participants who received the control vaccine will be offered vaccination against HPV-16/18 and hepatitis B, as appropriate. Vaccines to be used during cross-over immunization are the formulations licensed for use in Costa Rica. Cross-over immunization will be implemented by NCI and Costa Rican investigators under a separate protocol they have developed.

Implementation of cross-over immunization will require unblinding. Unblinding information and cross-over will not be offered to participants until they have been exited (closed out) from the present study and their data for this study have been frozen in the study databases. Exiting of participants in the trial will occur in an ongoing/batched manner as participants complete their 4-year participation in our study and after ongoing SAEs or pregnancies are resolved, or on October 3, 2010 (10 months after the 4-year anniversary for the last woman enrolled), whichever comes first. This will ensure that study blind is maintained for individual participants until their data for the final analysis is frozen.

If cross-over immunization cannot be offered to a subject (e.g., because of medical reasons) or the subject refuses cross-over immunization, unblinding information will be provided after the participant has exited the current study and her data has been frozen. This will occur at the time of the first cross-over implementation visit, at which time participation/eligibility for cross-over immunization will be determined.

3.17.3 Blood Collection

…This third blood draw will be collected for ancillary research purposes on a subset of participants, including a random sample of 10% of participants at enrollment and at their 12 and 36 month visits (to document the distribution of the immune responses in our population before and after vaccination), women referred to accelerated screening visits at six-month intervals through ***March 31, 2009 (Amended: 10 September 2009)*** (this group would have the additional blood sample collected annually), women who are referred for colposcopic evaluation and require a biopsy or LEEP, and women enrolled in the immunogenicity subcohort of our trial after March 1, 2006.

3.19.2 Histology

In addition to the Costa Rican diagnosis, ***an*** NCI ***designated (Amended: 10 September 2009)*** pathologist *(*Amended: 13 Nov 2008***)*** with expertise in cervical pathology will periodically diagnose all histological specimens, blinded to the initial Costa Rican diagnosis.

4.1 Overview

Additional exploratory tertiary and ancillary analyses will be conducted by the NCI and Costa Rican investigators and will evaluate numerous issues, as described in the Tertiary Objectives and Ancillary Analyses & Studies sections of this protocol (Section 2.3 and Section 2.4).

No interim statistical analyses for ***the primary*** efficacy ***endpoint*** are planned at this time ***(Amended: 10 September 2009)***.

Adverse events (AEs) observed in the course of the trial will be carefully monitored. Both expected and unexpected AEs will be examined. Rates of specific solicited, unsolicited, and serious AEs (SAEs) will be compared by treatment arm and evaluated.

These analyses will be performed throughout the trial by the DSMB (study personnel will remain blinded) and will not affect the overall alpha level of the study.

4.6 Interim Analysis

No interim statistical analyses for efficacy ~~are~~ ***were*** planned ***at the beginning of the trial (Amended: 10 September 2009)***. A need for an interim analysis for efficacy may arise during the trial. Should this occur, the study protocol will be amended, and the amendment submitted to the US FDA for review. To assist the DSMB in making decisions regarding interim analyses in the future, a set of guidelines are provided separately from this protocol in the DSMB Charter.

A Statistical Analysis Plan (SAP) for initial analyses that focuses on virological endpoints has been submitted to the FDA. The analyses proposed within are largely exploratory in nature, but there is one overlap between an objective in the SAP for initial analysis and secondary objective #5 listed in this protocol (i.e., for secondary objective 5 listed in this protocol, we are proposing to conduct an interim analysis). Given this overlap, for secondary objective #5 listed in this protocol, the following alpha adjustment plan will be implemented: The overall alpha of 0.025 (one-sided) will be split into 0.001 for the interim analysis proposed in the SAP for initial analyses and 0.024 for the final analysis. As specified in more detail in the SAP for initial analysis (Table 3 of that document), power (assuming 80% vaccine efficacy and 20 or more total events) should remain above 85-90% for the final analysis of secondary objective #5 (Amended: 10 September 2009).

No stopping rule will be applied at this stage; blinding will be maintained and follow-up will continue for all subjects until Month 48 (Amended: 10 September 2009).

Appendix D

11. Pregnancy

…All new pregnancy and pregnancy outcome information for study participants, whether or not an SAE is involved, will be transmitted to the Westat Regulatory Associate via a read-only electronic mail report within 5 business days (i.e., no longer than ~~**120**~~ ***40*** business hours) ***(Amended: 10 September 2009).***
